# Supplementary material for: Effector-dependent structural transformation of a crystalline framework with allosteric effects on molecular recognition ability
Source: Nat Commun. 2023 Aug 10;14:4490. doi: 10.1038/s41467-023-40091-6 (PMC10415384; doi:10.1038/s41467-023-40091-6)
Supplement: Supplementary file 1 — Supplementary Information [file 41467_2023_40091_MOESM1_ESM.pdf]

Effector-dependent structural transformation of a crystalline framework with allosteric effects on molecular recognition ability

Ryunosuke Hayashi, Shohei Tashiro*,
Masahiro Asakura, Shinya Mitsui & Mitsuhiko Shionoya*

Department of Chemistry, Graduate School of Science, The University of Tokyo
7-3-1 Hongo, Bunkyo-ku, Tokyo 113-0033, Japan

1. Materials and methods
2. Single-crystal X-ray diffraction analysis of MMF in small ethers
3. Single-crystal X-ray diffraction analysis of effector-dependent MMF crystal transformation
4. Principal component analysis of MMF crystal structures
5. Powder X-ray diffraction analysis of MMF crystal transformation
6. Allosteric regulation of molecular recognition ability
7. Organic synthesis
8. References

1. Materials and Methods

The synthetic procedure of the macrocyclic hexamine ligand **L** is shown in our previous reports[1]. The crystallisation methods of MMF are described as well. For successive effector exchange or guest uptake experiments, MMF crystals were washed with pure acetonitrile in advance. The resulting MMF crystals dispersed in pure acetonitrile are referred to as as-crystalised MMF in this paper. All effector exchange or guest uptake experiments are performed under ambient atmosphere. Pentaethylene glycol dimethyl ether (pentaglyme) was synthesised by following the literature. Any other reagents were commercially available and used without further purification.

Single-crystal X-ray diffraction (SCXRD) analysis was conducted using an XtaLAB P200 system diffractometer with CuKα radiation, and the obtained data were analysed using an Olex2 software package[2] except for refinement, which was performed using SHELXL-2018/3 program suite[3]. In the crystal structures, several restraints and constraints of the geometrical and the atom displacement parameters were applied to MMF and the introduced molecules. Hydrogen atoms were placed at the calculated positions and refined using a riding model. The occupancies of guest molecules were refined based on electron densities using free variables. The relatively high *R*-factors of MMF crystals can be attributed to the large intrinsic voids filled with disordered solvent as well as the moderate crystal quality through the lattice expansion. The MMF unit-space is defined as the space of one repeating unit in one dimensional channel. A Mercury program was used for displaying analysed crystal structures and for calculating the void space. The void space in the unit cell represents a cavity large enough to accommodate a spherical probe of radius 1.2 Å. For calculation of the void space, the contact surface of the MMF structures omitting adsorbed guest molecules except those in the allosteric site was used with the grid spacing of 0.7 Å. A ShelXle program[4] was used for obtaining the electron density distribution maps. Powder X-ray diffraction measurement was conducted using a Rigaku SmartLab diffractometer with CuKα radiation. ^{1}H NMR measurements were carried out with Bruker AVANCE 500 spectrometer (500 MHz). The resulting ^{1}H NMR spectra were calibrated based on the signal of CD_3SOCHD_2 = 2.50 ppm in DMSO-d_6, and $Si(CH_3)_4$ = 0 ppm in $CDCl_3$.

2. Single-crystal X-ray diffraction analysis of MMF in small ethers

•1,2-Dimethoxyethane (DME)

Soaking procedure: As-crystalised MMF crystals were soaked in 1,2-dimethoxyethane (DME) for 4 h at 20 °C, and one of the crystals was picked up and immediately mixed with paratone oil to measure single-crystal XRD.

Crystal data for $(Pd_3\mathbf{L}Cl_6)_2\cdot(\mathbf{DME})_{2.5}\cdot(H_2O)_4$: $C_{94}H_{109}Cl_{12}N_{12}O_9Pd_6$, $F_w = 2614.73$, crystal dimensions 0.17 × 0.12 × 0.07 mm^3, monoclinic, space group $P2_1/c$, $a = 22.0352(2)$, $b = 51.6545(7)$, $c = 14.57130(10)$ Å, $\beta = 97.3010(10)°$, $V = 16450.8(3)$ Å^3, $Z = 4$, $\rho_{calcd} = 1.056$ g cm^{-3}, $\mu = 7.280$ mm^{-1}, $T = 93$ K, λ(CuKα) = 1.54187 Å, $2\theta_{max} = 136.5°$, 204113/ 32319 reflections collected/unique ($R_{int} = 0.0686$), $R_1 = 0.1351$ ($I > 2\sigma(I)$), $wR_2 = 0.4241$ (for all data), GOF = 1.712, largest diff. peak and hole 4.516/–3.408 eÅ^{-3}. CCDC deposit number 2223883

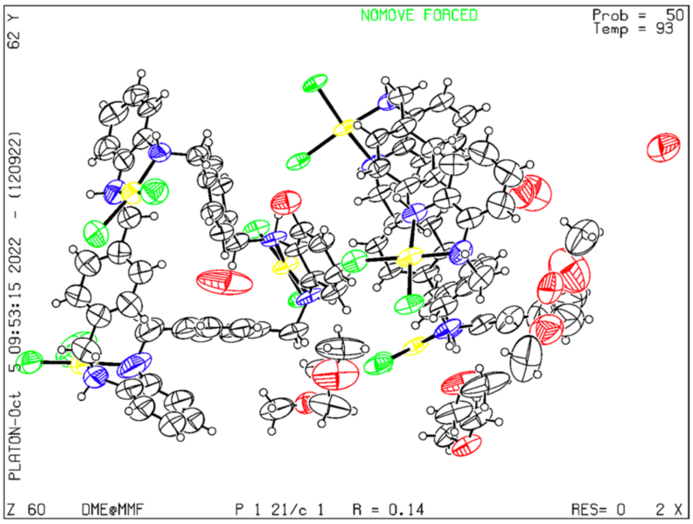

Supplementary Fig. 1 ORTEP drawing of the structurally extended MMF soaked in **DME** at the 50% probability level. Color: C black, N blue, O red, Cl green and Pd yellow. This figure was produced by the checkCIF report of the International Union of Crystallography.

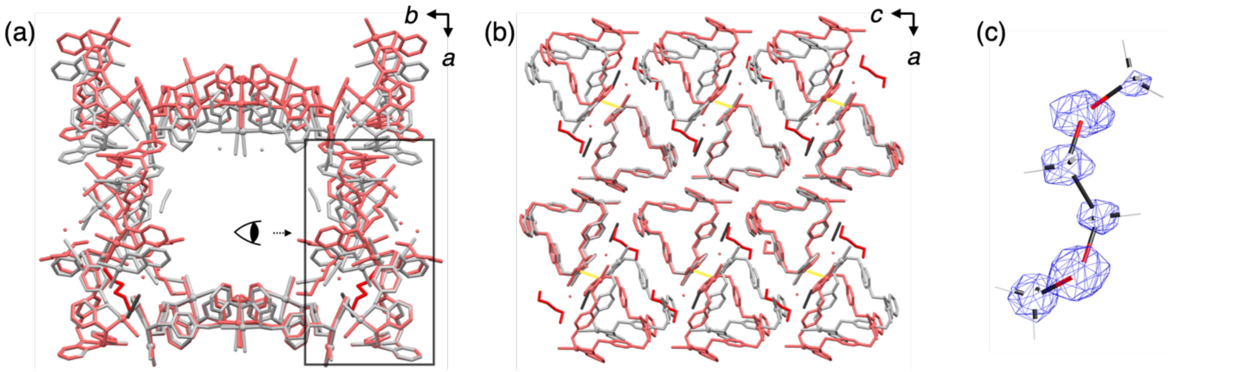

Supplementary Fig. 2 (a), (b) Structural overlap of MMF before and after the transformation with DME. (a) Unit-space structure and (b) two-dimensional network of *syn*-Pd-macrocycles viewed from the position of the

eye in (a), Grey line indicates as-crystalised MMF in acetonitrile and red line indicates structurally-extended MMF in DME. (c) The electron density map of DME (100% occupancy) adsorbed in the allosteric binding site (contour level; 2.0 eÅ^{-3}).

•1,4-Dioxane

Soaking procedure: As-crystalised MMF crystals were soaked in 1,4-dioxane at 20 °C for 2 days, and one of the crystals was picked up and immediately mixed with paratone oil to measure single-crystal XRD.

Crystal data for $(Pd_3\mathbf{L}Cl_6)_4\cdot(\mathbf{1,4\text{-}dioxane})_{4.23}\cdot(H_2O)_8$: $C_{184.99}H_{201.98}Cl_{24}N_{24}O_{16.50}Pd_{12}$, F_w = 5153.07, crystal dimensions 0.25 × 0.12 × 0.11 mm^3, monoclinic, space group $P2_1$, a = 14.36740(10), b = 53.9518(12), c = 19.9769(3) Å, β = 90.1670(10)°, V = 15485.0(4) Å^3, Z = 2, ρ_{calcd} = 1.105 g cm^{-3}, μ = 7.725 mm^{-1}, T = 93 K, λ(CuKα) = 1.54187 Å, $2\theta_{max}$ = 136.5°, 189853/ 55862 reflections collected/unique (R_{int} = 0.0538), R_1 = 0.1309 ($I > 2\sigma(I)$), wR_2 = 0.3683 (for all data), GOF = 1.340, largest diff. peak and hole 4.264/–1.741 eÅ^{-3}, Flack parameter = 0.423(19). CCDC deposit number 2223884

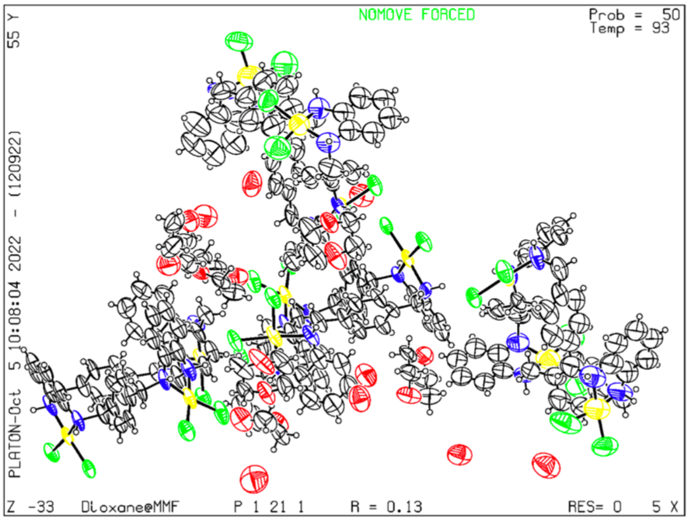

Supplementary Fig. 3 ORTEP drawing of the structurally extended MMF soaked in **1,4-dioxane** at the 50% probability level. Color: C black, N blue, O red, Cl green and Pd yellow. This figure was produced by the checkCIF report of the International Union of Crystallography.

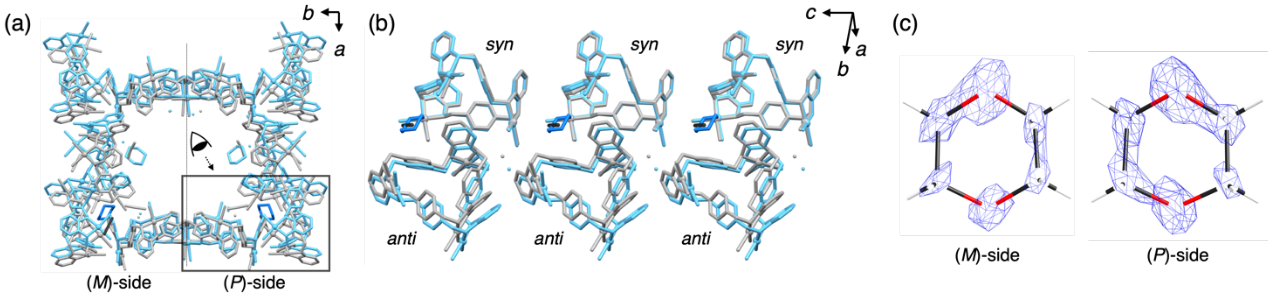

Supplementary Fig. 4 (a), (b) Structural overlap of MMF before and after the transformation with 1,4-dioxane. (a) The unit-space structure and (b) *syn*-Pd-macrocycles and *anti*-Pd-macrocycles around the allosteric binding site on the (*P*)-side viewed from the position of the eye in (a) ((*P*/*M*)-side is defined as the half of the unit-space separated by the glide plane, containing the (*P*/*M*)-*anti*-Pd-macrocycles). Grey line indicates as-crystalised MMF in acetonitrile and blue line indicates structurally-extended MMF in 1,4-dioxane. (c) The electron density maps of 1,4-dioxane (100% occupancy each) adsorbed in the allosteric binding sites (contour level; 2.0 eÅ^{-3}).

•Diethyleneglycol dimethyl ether (Diglyme)

Soaking procedure: As-crystalised MMF crystals were soaked in diethyleneglycol dimethyl ether (diglyme) for 1 day at 20 °C, and one of the crystals was picked up and immediately mixed with paratone oil to measure single-crystal XRD.

Crystal data for $(Pd_3\mathbf{L}Cl_6)_2\cdot(\mathbf{diglyme})_{0.56}\cdot(H_2O)_{4.25}$: $C_{87.35}H_{91.83}Cl_{12}N_{12}O_{5.93}Pd_6$, F_w = 2468.45, crystal dimensions 0.41 × 0.16 × 0.15 mm^3, monoclinic, space group $P2_1/c$, a = 19.3908(2), b = 52.1158(5), c = 14.3652(1) Å, β = 91.507(1)°, V = 14512.0(2) Å^3, Z = 4, ρ_{calcd} = 1.130 g cm^{-3}, μ = 8.210 mm^{-1}, T = 93 K, λ(CuKα) = 1.54187 Å, $2\theta_{max}$ = 136.5°, 178797/ 28543 reflections collected/unique (R_{int} = 0.0698), R_1 = 0.1173 ($I > 2\sigma(I)$), wR_2 = 0.3359 (for all data), GOF = 1.202, largest diff. peak and hole 2.259/–2.104 eÅ^{-3}. CCDC deposit number 2223885

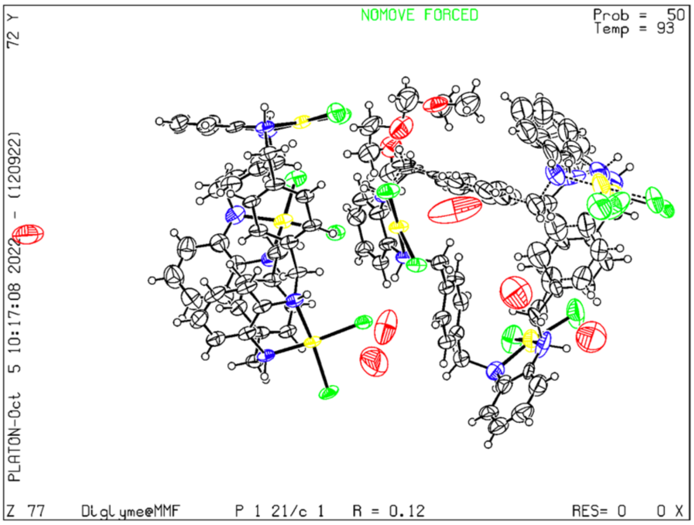

Supplementary Fig. 5 ORTEP drawing of MMF soaked in **diglyme** at the 50% probability level. Color: C black, N blue, O red, Cl green and Pd yellow. This figure was produced by the checkCIF report of the International Union of Crystallography.

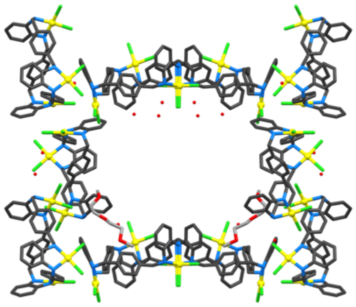

Supplementary Fig. 6 The unit-space structure of not-extended MMF soaked in diglyme. Adsorption of diglyme was observed on the void space of MMF channel, while water molecules were accommodated in the allosteric site.

•(*rac*)-1,2-Dimethoxypropane (DMP)

Soaking procedure: As-crystalised MMF crystals were soaked in (*rac*)-1,2-dimethoxypropane (DMP) at 20 °C for 12 h, and one of the crystals was picked up and immediately mixed with paratone oil to measure single-crystal XRD.

Crystal data for $(Pd_3\mathbf{L}Cl_6)_2\cdot(H_2O)_{3.5}$: $C_{84}H_{84}Cl_{12}N_{12}O_{3.50}Pd_6$, F_w = 2381.43, crystal dimensions 0.22 × 0.15 × 0.10 mm^3, monoclinic, space group $P2_1/c$, a = 19.4044(2), b = 51.8683(8), c = 14.2285(1) Å, β = 91.972(1)°, V = 14312.1(3) $Å^3$, Z = 4, ρ_{calcd} = 1.105 g cm^{-3}, μ = 8.296 mm^{-1}, T = 93 K, λ(CuKα) = 1.54187 Å, $2\theta_{max}$ = 136.5°, 143395/ 26957 reflections collected/unique (R_{int} = 0.0602), R_1 = 0.1304 ($I > 2\sigma(I)$), wR_2 = 0.3883 (for all data), GOF = 1.109, largest diff. peak and hole 2.041/–1.838 $eÅ^{-3}$. CCDC deposit number 2223886

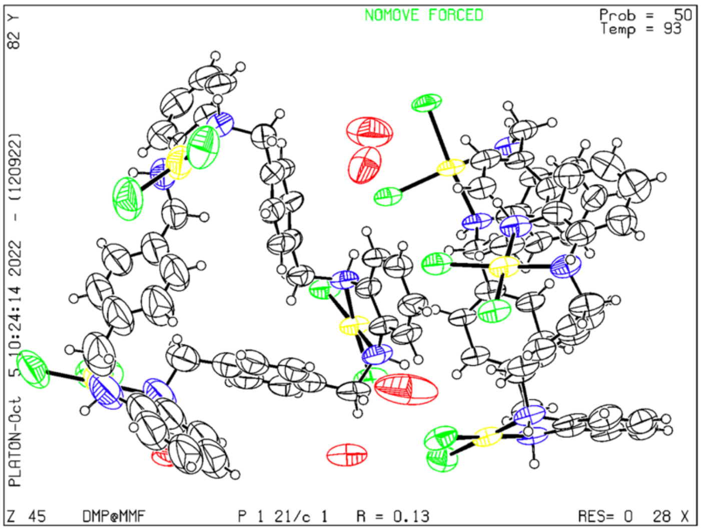

Supplementary Fig. 7 ORTEP drawing of MMF soaked in **DMP** at the 50% probability level. Color: C black, N blue, O red, Cl green and Pd yellow. This figure was produced by the checkCIF report of the International Union of Crystallography.

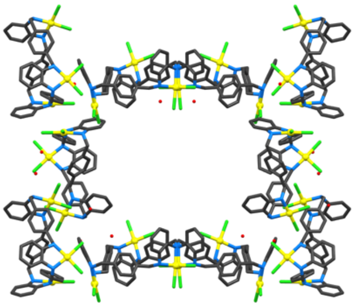

Supplementary Fig. 8 The unit-space structure of not-extended MMF soaked in DMP. Adsorption of DMP was not observed, while water molecules were accommodated in the allosteric site.

•1,2-Diethoxylethane (DEE)

Soaking procedure: As-crystalised MMF crystals were soaked in 1,2-diethoxyethane (DEE) at 20 °C for 1 day, and one of the crystals was picked up and immediately mixed with paratone oil to measure single-crystal XRD.

Crystal data for $(Pd_3\mathbf{L}Cl_6)_2\cdot(\mathbf{DEE})_{0.5}\cdot(H_2O)_{7.25}$: $C_{87.01}H_{91.03}Cl_{12}N_{12}O_{8.26}Pd_6$, F_w = 2500.77, crystal dimensions 0.43 × 0.20 × 0.10 mm^3, monoclinic, space group $P2_1/c$, a = 19.4746(3), b = 52.2739(8), c = 14.2951(1) Å, β = 91.235(1)°, V = 14549.2(3) Å^3, Z = 4, ρ_{calcd} = 1.142 g cm^{-3}, μ = 8.208 mm^{-1}, T = 93 K, λ(CuKα) = 1.54187 Å, $2\theta_{max}$ = 136.5°, 94767/ 27538 reflections collected/unique (R_{int} = 0.0804), R_1 = 0.1379 ($I > 2\sigma(I)$), wR_2 = 0.3681 (for all data), GOF = 1.168, largest diff. peak and hole 3.255/–2.157 eÅ^{-3}. CCDC deposit number 2223887

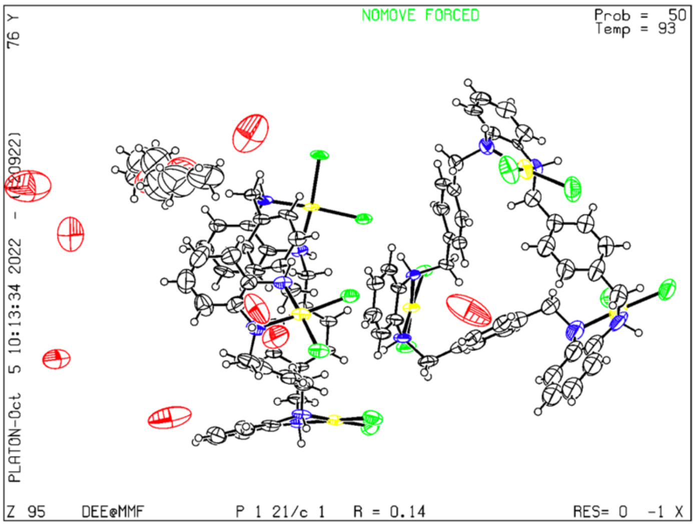

Supplementary Fig. 9 ORTEP drawing of MMF soaked in **DEE** at the 50% probability level. Color: C black, N blue, O red, Cl green and Pd yellow. This figure was produced by the checkCIF report of the International Union of Crystallography.

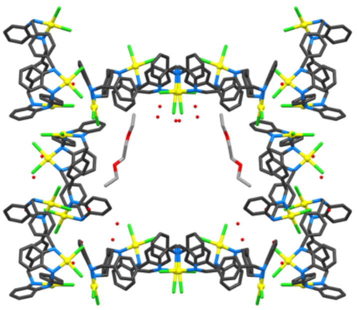

Supplementary Fig. 10 The unit-space structure of not-extended MMF soaked in DEE. Adsorption of DEE was observed on the void space of MMF channel, while water molecules were accommodated in the allosteric site.

•Methyl *n*-butyl ether (MBE)

Soaking procedure: As-crystalised MMF crystals were soaked in methyl *n*-butyl ether (MBE) at 20 °C for 1 h, and one of the crystals was picked up and immediately mixed with paratone oil to measure single-crystal XRD. The resulting low-quality crystals exhibited the collapse of the porous structures possibly due to the high volatility of MBE.

•1,3-Dioxolane

Soaking procedure: As-crystalised MMF crystals were soaked in 1,3-dioxolane at 20 °C for 9 h, and one of the crystals was picked up and immediately mixed with paratone oil to measure single-crystal XRD.

Crystal data for $(Pd_3\mathbf{L}Cl_6)_4$·(**1,3-dioxolane**)$_2$·$(H_2O)_2$: $C_{174}H_{180}Cl_{24}N_{24}O_6Pd_{12}$, F_w = 4831.01, crystal dimensions 0.29 × 0.22 × 0.12 mm^3, monoclinic, space group $P2_1$, a = 14.4204(1), b = 52.2274(11), c = 19.6721(2) Å, β = 90.323(1)°, V = 14815.6(4) Å^3, Z = 2, ρ_{calcd} = 1.083 g cm^{-3}, μ = 8.017 mm^{-1}, T = 93 K, λ(CuKα) = 1.54187 Å, $2\theta_{max}$ = 136.5°, 399456/ 57986 reflections collected/unique (R_{int} = 0.1122), R_1 = 0.1551 ($I > 2\sigma(I)$), wR_2 = 0.4242 (for all data), GOF = 1.703, largest diff. peak and hole 3.676/–3.874 eÅ^{-3}, Flack parameter = 0.44(2). CCDC deposit number 2223888

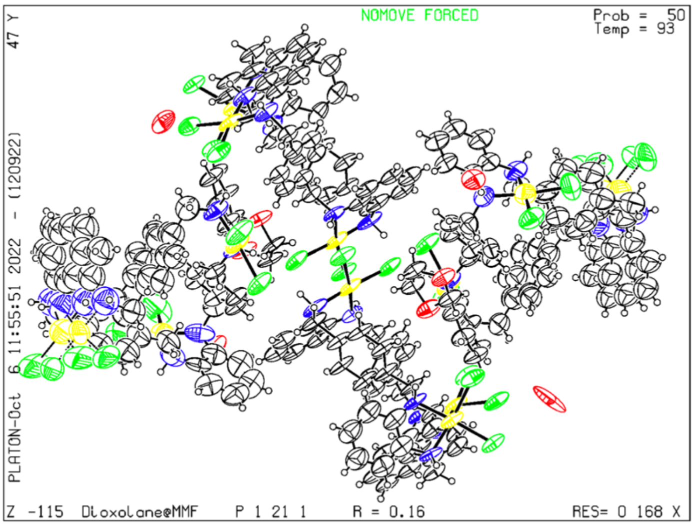

Supplementary Fig. 11 ORTEP drawing of the structurally extended MMF soaked in **1,3-dioxolane** at the 50% probability level. Color: C black, N blue, O red, Cl green and Pd yellow. This figure was produced by the checkCIF report of the International Union of Crystallography.

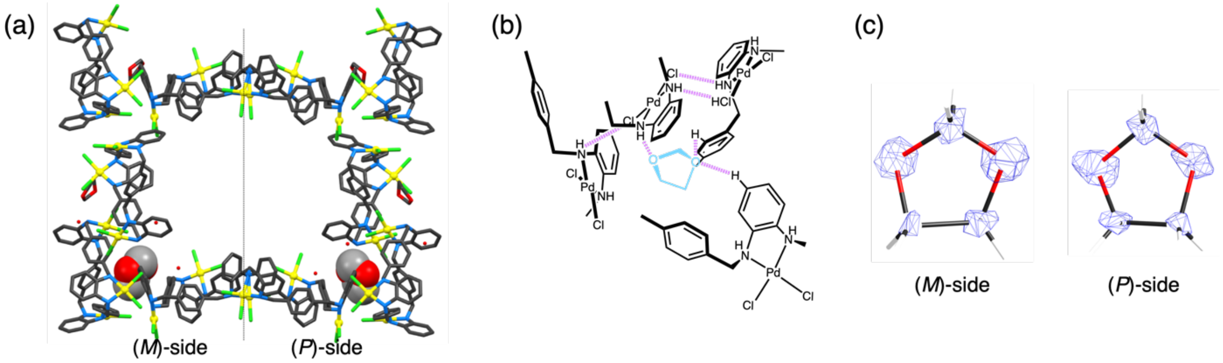

Supplementary Fig. 12 (a) The unit-space structure of the MMF that accommodates 1,3-dioxolane shown in the CPK model as an effector. (b) Interaction patterns around the allosteric site of MMF and 1,3-dioxolane. (c) The electron density maps of 1,3-dioxolane (100% occupancy each) adsorbed in the allosteric binding sites (contour level; 3.0 eÅ^{-3}).

•Tetrahydropyran (THP)

Soaking procedure: As-crystalised MMF crystals were soaked in tetrahydropyran (THP) at 20 °C for 1 h, and one of the crystals was picked up and immediately mixed with paratone oil to measure single-crystal XRD.

Crystal data for $(Pd_3\mathbf{L}Cl_6)_2\cdot(\mathbf{THP})_{0.83}\cdot(H_2O)_4$: $C_{88.14}H_{92.29}Cl_{12}N_{12}O_{4.83}Pd_6$, F_w = 2460.84, crystal dimensions 0.53 × 0.25 × 0.10 mm^3, monoclinic, space group $P2_1/c$, a = 19.5140(3), b = 51.539(2), c = 14.2668(2) Å, β = 91.6733(13)°, V = 14342.5(6) Å^3, Z = 4, ρ_{calcd} = 1.140 g cm^{-3}, μ = 8.300 mm^{-1}, T = 93 K, λ(CuKα) = 1.54187 Å, $2\theta_{max}$ = 136.5°, 83448/ 26027 reflections collected/unique (R_{int} = 0.0479), R_1 = 0.1769 ($I > 2\sigma(I)$), wR_2 = 0.4374 (for all data), GOF = 1.110, largest diff. peak and hole 2.429/–2.754 eÅ^{-3}. CCDC deposit number

2223889

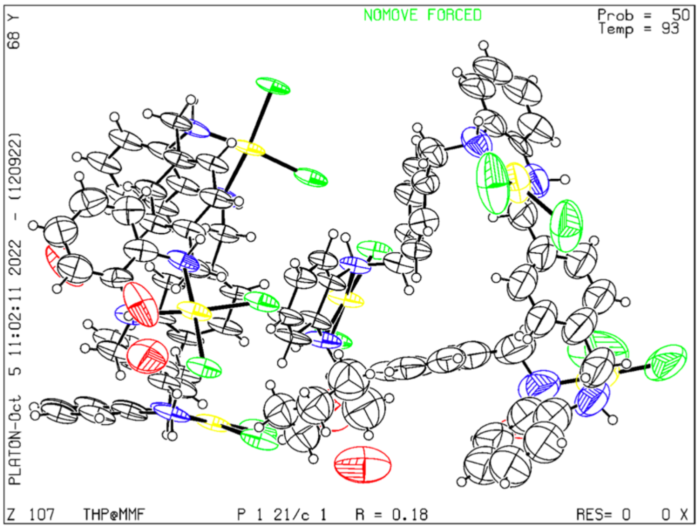

Supplementary Fig. 13 ORTEP drawing of MMF soaked in **THP** at the 50% probability level. Color: C black, N blue, O red, Cl green and Pd yellow. This figure was produced by the checkCIF report of the International Union of Crystallography.

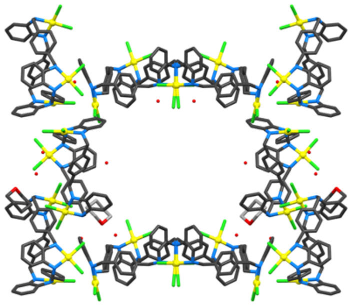

Supplementary Fig. 14 The unit-space structure of not-extended MMF soaked in THP. Adsorption of THP was observed on the void space of MMF channel, while water molecules were accommodated in the allosteric site.

3. Single-crystal X-ray diffraction analysis of effector-dependent MMF crystal transformation

•Triethyleneglycol dimethyl ether (triglyme)

Soaking procedure: As-crystalised MMF crystals were soaked in triethyleneglycol dimethyl ether (triglyme) at 20 °C for 6 h, and one of the crystals was picked up and immediately mixed with paratone oil to measure single-crystal XRD.

Crystal data for $(Pd_3\mathbf{L}Cl_6)_2\cdot(\mathbf{triglyme})_{0.5}\cdot(H_2O)_1$: $C_{88}H_{93}Cl_{12}N_{12}O_3Pd_6$, F_w = 2430.54, crystal dimensions 0.13 × 0.07 × 0.06 mm^3, monoclinic, space group $P2_1/c$, a = 22.4948(2), b = 52.6766(12), c = 14.42440(13) Å, β = 91.6733(13)°, V = 16956.0(4) Å^3, Z = 4, ρ_{calcd} = 0.952 g cm^{-3}, μ = 7.007 mm^{-1}, T = 93 K, λ(CuKα) = 1.54187

Å, $2\theta_{max}$ = 136.5°, 86956/ 30904 reflections collected/unique (R_{int} = 0.0529), R_1 = 0.1224 ($I > 2\sigma(I)$), wR_2 = 0.3851 (for all data), GOF = 1.334, largest diff. peak and hole 2.294/–1.185 eÅ^{-3}. CCDC deposit number 2223897

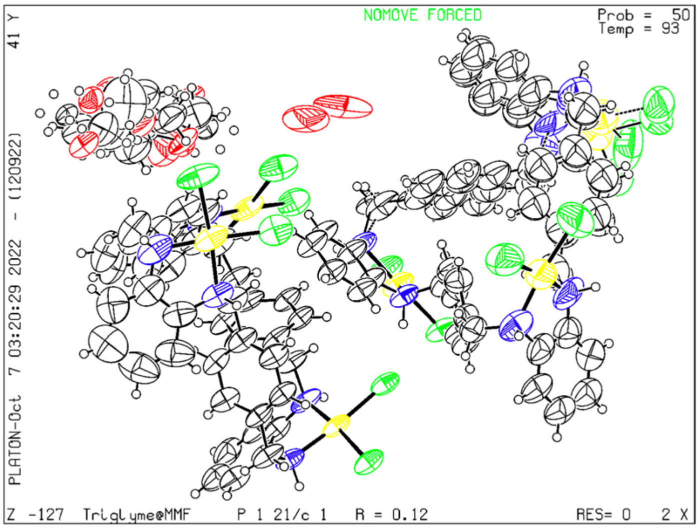

Supplementary Fig. 15 ORTEP drawing of the structurally extended MMF soaked in **triglyme** at the 50% probability level. Color: C black, N blue, O red, Cl green and Pd yellow. This figure was produced by the checkCIF report of the International Union of Crystallography.

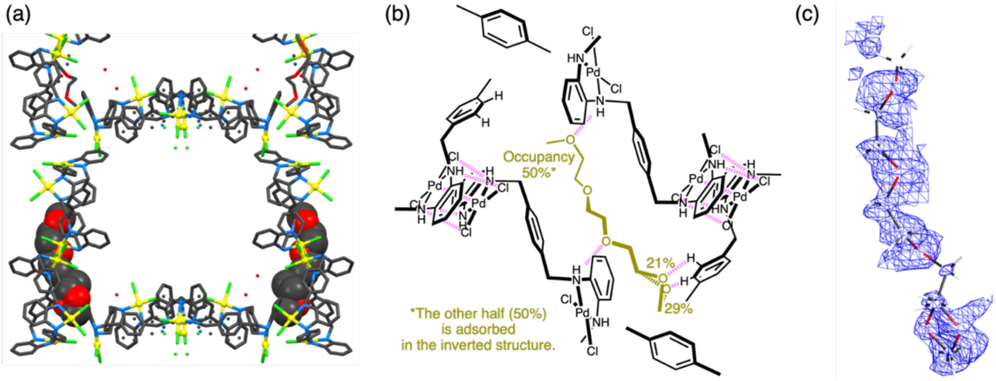

Supplementary Fig. 16 (a) The unit-space structure of the MMF that accommodates triglyme as an effector. The triglyme shown in the CPK model was adsorbed across two allosteric sites in adjacent channels. (b) Interaction patterns around the allosteric site of MMF and triglyme. The occupancy of triglyme is set to be 0.5, and the other half exist in the inverted structure. (c) The electron density map of triglyme (50% occupancy for one molecule) adsorbed in the allosteric binding site (contour level; 0.7 eÅ^{-3}).

•Tetraethyleneglycol dimethyl ether (tetraglyme)

Soaking procedure: As-crystalised MMF crystals were firstly soaked in dichloromethane in order to remove acetonitrile from the supernatant. Afterwards, the crystals were transferred into tetraethyleneglycol dimethyl ether (tetraglyme) to be soaked at 20 °C for 6 h, and one of the crystals was picked up and immediately mixed

with paratone oil to measure single-crystal XRD.

Crystal data for $(Pd_3\mathbf{L}Cl_6)_2\cdot(\mathbf{tetraglyme})_{0.89}\cdot(H_2O)_{1.5}$: $C_{90.56}H_{97.72}Cl_{12}N_{12}O_{4.78}Pd_6$, F_w = 2494.46, crystal dimensions 0.18 × 0.08 × 0.06 mm^3, monoclinic, space group $P2_1/c$, a = 22.5704(5), b = 52.5407(15), c = 14.42350(18) Å, β = 97.019(2)°, V = 16976.1(7) Å^3, Z = 4, ρ_{calcd} = 0.976 g cm^{-3}, μ = 7.017 mm^{-1}, T = 93 K, λ(CuKα) = 1.54187 Å, $2\theta_{max}$ = 136.5°, 91802/ 30848 reflections collected/unique (R_{int} = 0.0446), R_1 = 0.1107 ($I > 2\sigma(I)$), wR_2 = 0.3630 (for all data), GOF = 1.260, largest diff. peak and hole 2.441/–1.400 eÅ^{-3}. CCDC deposit number 2223898

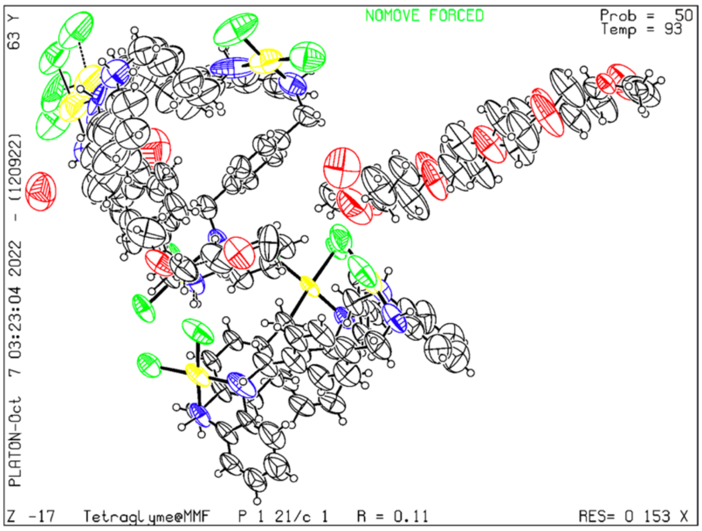

Supplementary Fig. 17 ORTEP drawing of the structurally extended MMF soaked in **tetraglyme** at the 50% probability level. Color: C black, N blue, O red, Cl green and Pd yellow. This figure was produced by the checkCIF report of the International Union of Crystallography.

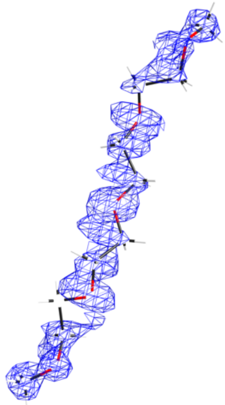

Supplementary Fig. 18 The electron density map of tetraglyme (50% occupancy for one molecule) adsorbed in the allosteric binding site (contour level; 1.2 eÅ^{-3}).

•Pentaethyleneglycol dimethyl ether (pentaglyme)

Soaking procedure: As-crystalised MMF crystals were firstly soaked in dichloromethane in order to remove

acetonitrile from the supernatant. Afterwards, the crystals were transferred into pentaethyleneglycol dimethyl ether (pentaglyme) to be soaked at 20 °C for 5 days, and one of the crystals was picked up and immediately mixed with paratone oil to measure single-crystal XRD.

Crystal data for $(Pd_3\mathbf{L}Cl_6)_2\cdot(\mathbf{pentaglyme})_{1.41}\cdot(H_2O)_{0.5}$: $C_{97.65}H_{112.89}Cl_{12}N_{12}O_{7.33}Pd_6$, F_w = 2635.76, crystal dimensions 0.30 × 0.14 × 0.06 mm^3, monoclinic, space group $P2_1/c$, a = 22.63060(17), b = 52.7556(6), c = 14.43340(11) Å, β = 97.4118(7)°, V = 17087.9(7) Å^3, Z = 4, ρ_{calcd} = 1.025 g cm^{-3}, μ = 7.005 mm^{-1}, T = 93 K, λ(CuKα) = 1.54187 Å, $2\theta_{max}$ = 136.5°, 179174/ 31112 reflections collected/unique (R_{int} = 0.0527), R_1 = 0.1101 ($I > 2\sigma(I)$), wR_2 = 0.3857 (for all data), GOF = 1.653, largest diff. peak and hole 2.475/–2.497 eÅ^{-3}. CCDC deposit number 2223899

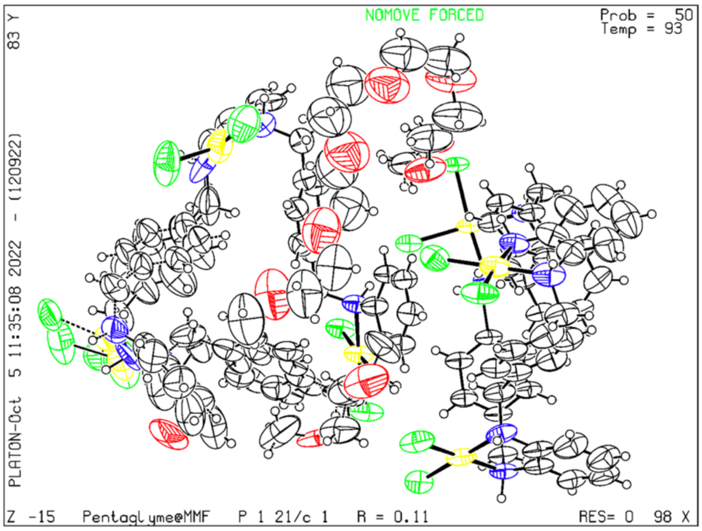

Supplementary Fig. 19 ORTEP drawing of the structurally extended MMF soaked in **pentaglyme** at the 50% probability level. Color: C black, N blue, O red, Cl green and Pd yellow. This figure was produced by the checkCIF report of the International Union of Crystallography.

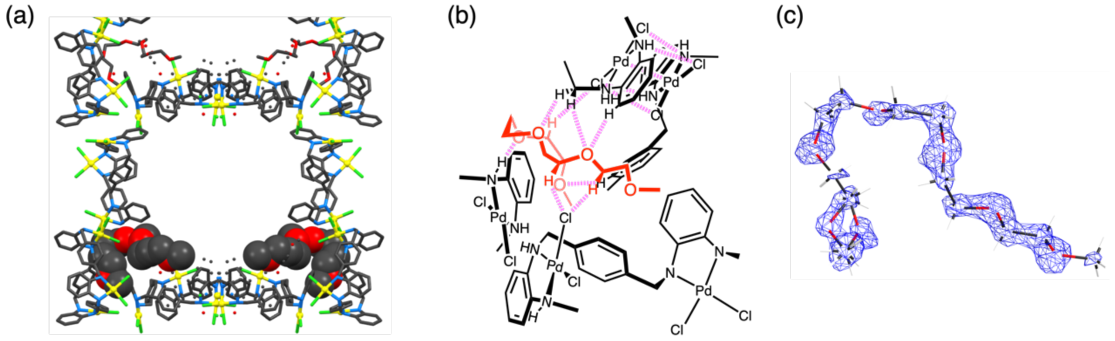

Supplementary Fig. 20 (a) The unit-space structure of the MMF that accommodates the pentaglyme shown in the CPK model as an effector. (b) Interaction patterns around the allosteric site of MMF and pentaglyme. (c) The electron density map of pentaglyme (100% occupancy) adsorbed in the allosteric binding site (contour level; 1.2 eÅ^{-3}).

•Diethyleneglycol (diglycol)

Soaking procedure: As-crystalised MMF crystals were firstly soaked in dichloromethane in order to remove acetonitrile from the supernatant. Afterwards, the crystals were transferred into diethyleneglycol (diglycol) to be soaked at 20 °C for 2 h, and one of the crystals was picked up and immediately mixed with paratone oil to measure single-crystal XRD.

Crystal data for $(Pd_3\mathbf{L}Cl_6)_2\cdot(\mathbf{diglycol})_1\cdot(H_2O)_{1.75}$: $C_{88}H_{92}Cl_{12}N_{12}O_{4.75}Pd_6$, F_w = 2457.53, crystal dimensions 0.08 × 0.06 × 0.04 mm^3, monoclinic, space group $P2_1/c$, a = 22.7088(3), b = 53.5645(9), c = 14.4623(2) Å, β = 97.595(1)°, V = 17437.4(4) Å^3, Z = 4, ρ_{calcd} = 0.936 g cm^{-3}, μ = 6.826 mm^{-1}, T = 93 K, λ(CuKα) = 1.54187 Å, $2\theta_{max}$ = 136.5°, 95678/ 33462 reflections collected/unique (R_{int} = 0.0654), R_1 = 0.1205 ($I > 2\sigma(I)$), wR_2 = 0.3698 (for all data), GOF = 1.292, largest diff. peak and hole 3.334/–1.962 eÅ^{-3}. CCDC deposit number 2223900

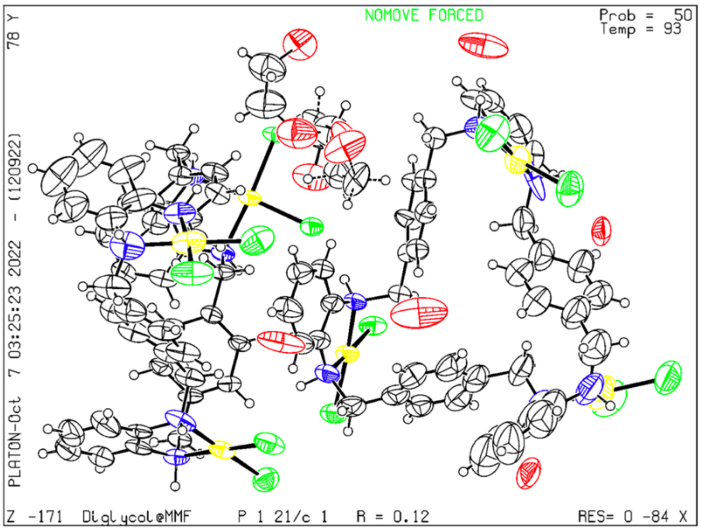

Supplementary Fig. 21 ORTEP drawing of the structurally extended MMF soaked in **diglycol** at the 50% probability level. Color: C black, N blue, O red, Cl green and Pd yellow. This figure was produced by the checkCIF report of the International Union of Crystallography.

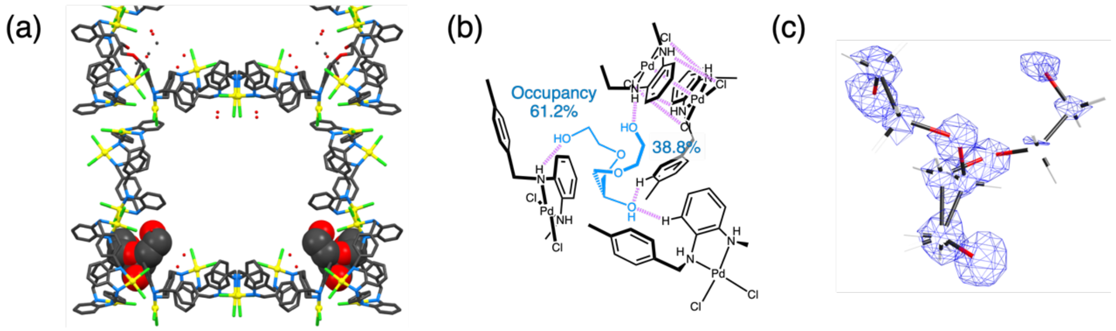

Supplementary Fig. 22 (a) The unit-space structure of the MMF that accommodates diglycol shown in the CPK model as an effector. (b) Interaction patterns around the allosteric site of MMF and diglycol. (c) The electron density map of diglycol (100% occupancy in total) adsorbed in the allosteric binding site (contour level; 1.2 eÅ^{-3}).

•Triethyleneglycol (triglycol)

Soaking procedure: As-crystalised MMF crystals were firstly soaked in dichloromethane in order to remove acetonitrile from the supernatant. Afterwards, the crystals were transferred into triethyleneglycol (triglycol) to be soaked at 20 °C for 1 day, and one of the crystals was picked up and immediately mixed with paratone oil to measure single-crystal XRD.

In the following crystal structure, strong electron density can be observed in the allosteric site. However, it can be assigned well possibly due to severe disorder of triglycol.

Crystal data for $(Pd_3\mathbf{L}Cl_6)_2\cdot$(**triglycol**)$_{0.5}\cdot(H_2O)_{2.37}$: $C_{86}H_{88}Cl_{12}N_{12}O_{3.87}Pd_6$, F_w = 2415.44, crystal dimensions 0.23 × 0.13 × 0.06 mm^3, monoclinic, space group $P2_1/c$, a = 22.7528(5), b = 53.1865(12), c = 14.3217(3) Å, β = 96.6701(19)°, V = 17214.0(7) Å^3, Z = 4, ρ_{calcd} = 0.932 g cm^{-3}, μ = 6.904 mm^{-1}, T = 93 K, λ(CuKα) = 1.54187 Å, $2\theta_{max}$ = 136.5°, 75567/ 30969 reflections collected/unique (R_{int} = 0.0712), R_1 = 0.1254 ($I > 2\sigma(I)$), wR_2 = 0.3932 (for all data), GOF = 1.359, largest diff. peak and hole 2.777/–1.933 eÅ^{-3}. CCDC deposit number 2223901

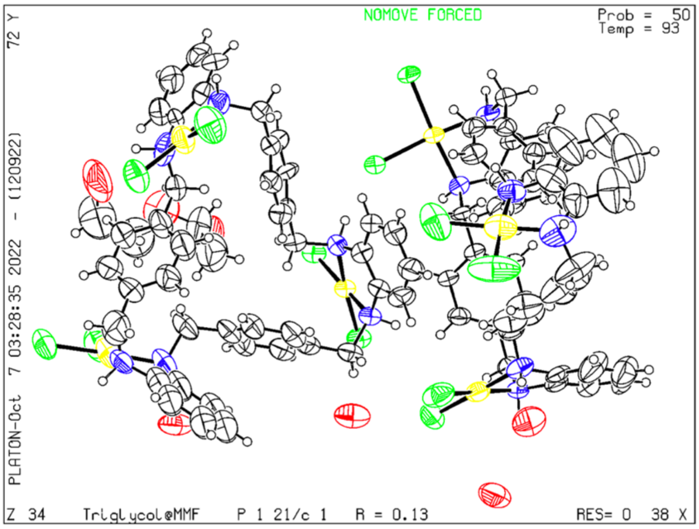

Supplementary Fig. 23 ORTEP drawing of the structurally extended MMF soaked in **triglycol** at the 50% probability level. Color: C black, N blue, O red, Cl green and Pd yellow. This figure was produced by the checkCIF report of the International Union of Crystallography.

•Tetraethyleneglycol (tetraglycol)

Soaking procedure: As-crystalised MMF crystals were firstly soaked in dichloromethane in order to remove acetonitrile from the supernatant. Afterwards, the crystals were transferred into tetraethyleneglycol (tetraglycol) to be soaked for 1 day at 20 °C, and one of the crystals was picked up and immediately mixed with paratone oil to measure single-crystal XRD.

In the following crystal structure, strong electron density can be observed in the allosteric site. However, it can be assigned well possibly due to severe disorder of tetraglycol.

Crystal data for $(Pd_3\mathbf{L}Cl_6)_2\cdot$(**tetraglycol**)$_{0.5}\cdot(H_2O)_{2.47}$: $C_{86}H_{88}Cl_{12}N_{12}O_{3.97}Pd_6$, F_w = 2416.92, crystal dimensions 0.24 × 0.09 × 0.07 mm^3, monoclinic, space group $P2_1/c$, a = 22.7974(3), b = 53.1454(6), c = 14.35080(13) Å, β = 96.7594(9)°, V = 17266.2(3) Å^3, Z = 4, ρ_{calcd} = 0.930 g cm^{-3}, μ = 6.884 mm^{-1}, T = 93 K, λ(CuKα) = 1.54187 Å, 2θ_{max} = 136.5°, 75194/ 31358 reflections collected/unique (R_{int} = 0.0345), R_1 = 0.0894 ($I > 2\sigma(I)$), wR_2 = 0.3012 (for all data), GOF = 1.231, largest diff. peak and hole 4.135/–2.913 eÅ^{-3}. CCDC deposit number 2223902

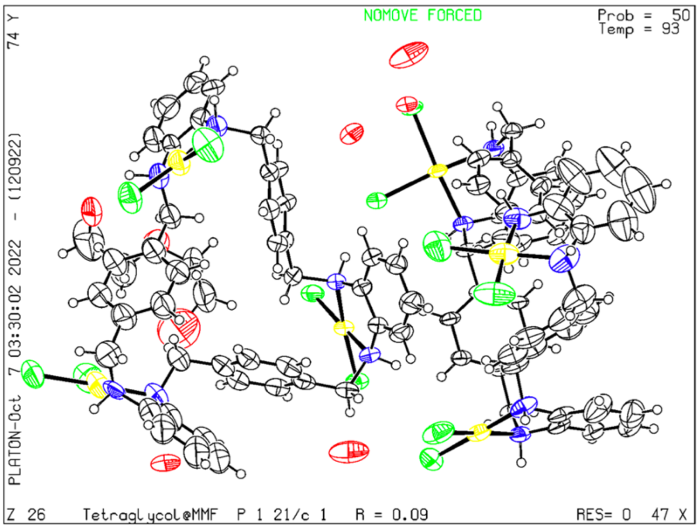

Supplementary Fig. 24 ORTEP drawing of the structurally extended MMF soaked in **tetraglycol** at the 50% probability level. Color: C black, N blue, O red, Cl green and Pd yellow. This figure was produced by the checkCIF report of the International Union of Crystallography.

•Pentaethyleneglycol (pentaglycol)

Soaking procedure: As-crystalised MMF crystals were firstly soaked in dichloromethane in order to remove acetonitrile from the supernatant. Afterwards, the crystals were transferred into pentaethyleneglycol (pentaglycol) to be soaked at 20 °C for 1 day, and one of the crystals was picked up and immediately mixed with paratone oil to measure single-crystal XRD.

In the following crystal structure, strong electron density can be observed in the allosteric site. However, it can be assigned well possibly due to severe disorder of pentaglycol.

Crystal data for $(Pd_3\mathbf{L}Cl_6)_2\cdot(H_2O)_{1.92}$: $C_{84}H_{84}Cl_{12}N_{12}O_{1.92}Pd_6$, F_w = 2356.11, crystal dimensions 0.17 × 0.14 × 0.05 mm^3, monoclinic, space group $P2_1/c$, a = 22.7235(3), b = 53.3038(8), c = 14.34090(16) Å, β = 96.8465(11)°, V = 17246.5(4) Å^3, Z = 4, ρ_{calcd} = 0.907 g cm^{-3}, μ = 6.873 mm^{-1}, T = 93 K, λ(CuKα) = 1.54187 Å, 2θ_{max} = 136.5°, 77005/ 31409 reflections collected/unique (R_{int} = 0.0574), R_1 = 0.1149 ($I > 2\sigma(I)$), wR_2 = 0.3499 (for all data),

GOF = 1.288, largest diff. peak and hole 3.314/–2.027 eÅ^{-3}. CCDC deposit number 2223903

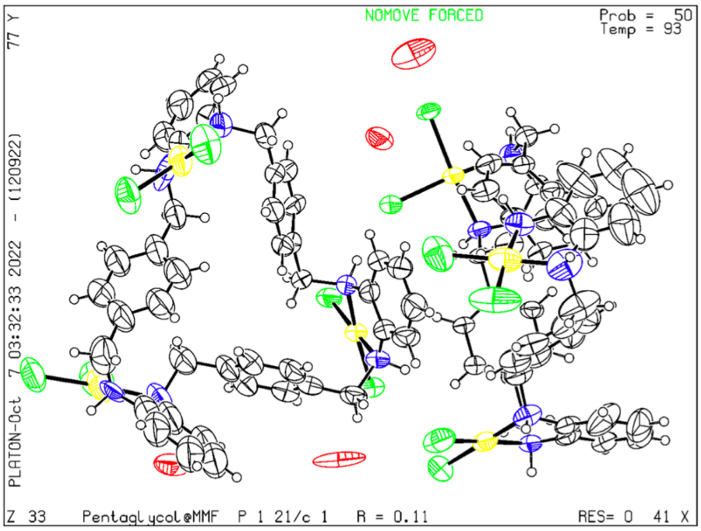

Supplementary Fig. 25 ORTEP drawing of the structurally extended MMF soaked in **pentaglycol** at the 50% probability level. Color: C black, N blue, O red, Cl green and Pd yellow. This figure was produced by the checkCIF report of the International Union of Crystallography.

•Hexaethyleneglycol (hexaglycol)

Soaking procedure: As-crystalised MMF crystals were firstly soaked in dichloromethane in order to remove acetonitrile from the supernatant. Afterwards, the crystals were transferred into hexaethyleneglycol (hexaglycol) to be soaked for 1 day at 20 °C, and one of the crystals was picked up and immediately mixed with paratone oil to measure single-crystal XRD.

In the following crystal structure, strong electron density can be observed in the allosteric site. However, it can be assigned well possibly due to severe disorder of hexaglycol.

Crystal data for $(Pd_3\mathbf{L}Cl_6)_2\cdot(H_2O)_{1.5}$: $C_{86}H_{88}Cl_{12}N_{12}O_{1.50}Pd_6$, F_w = 2349.43, crystal dimensions 0.36 × 0.09 × 0.07 mm^3, monoclinic, space group $P2_1/c$, a = 22.7975(3), b = 53.2185(7), c = 14.36140(14) Å, β = 96.9187(10)°, V = 17297.1(4) Å^3, Z = 4, ρ_{calcd} = 0.902 g cm^{-3}, μ = 6.850 mm^{-1}, T = 93 K, λ(CuKα) = 1.54187 Å, 2θ_{max} = 136.5°, 86574/ 31583 reflections collected/unique (R_{int} = 0.0781), R_1 = 0.1255 ($I > 2\sigma(I)$), wR_2 = 0.3545 (for all data), GOF = 1.282, largest diff. peak and hole 3.214/–1.395 eÅ^{-3}. CCDC deposit number 2223904

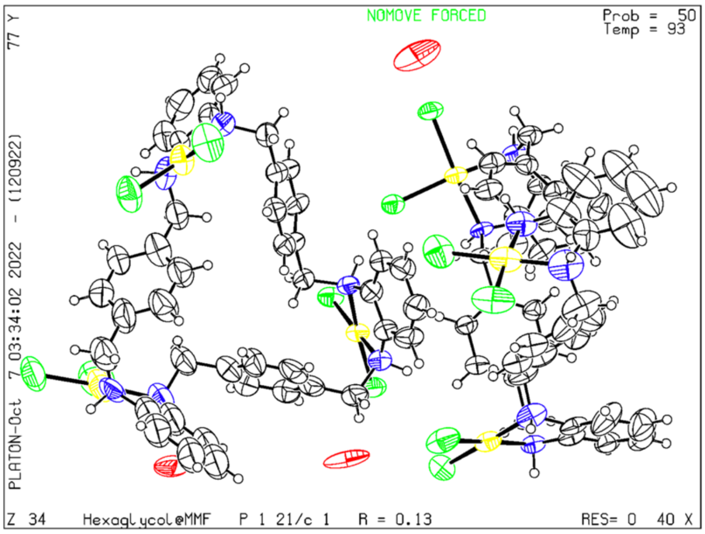

Supplementary Fig. 26 ORTEP drawing of the structurally extended MMF soaked in **hexaglycol** at the 50% probability level. Color: C black, N blue, O red, Cl green and Pd yellow. This figure was produced by the checkCIF report of the International Union of Crystallography.

•Acetylacetone (ACAC)

Soaking procedure: As-crystalised MMF crystals were soaked in acetylacetone (ACAC) at 20 °C for 6 h, and one of the crystals was picked up and immediately mixed with paratone oil to measure single-crystal XRD.

Crystal data for $(Pd_3\mathbf{L}Cl_6)_2\cdot(\mathbf{ACAC})_{1.63}\cdot(H_2O)_2$: $C_{92.26}H_{96.22}Cl_{12}N_{12}O_{5.31}Pd_6$, F_w = 2521.86, crystal dimensions 0.26 × 0.17 × 0.12 mm^3, monoclinic, space group $P2_1/c$, a = 22.7525(4), b = 53.2057(9), c = 14.45570(15) Å, β = 97.245(1)°, V = 17359.8(5) Å^3, Z = 4, ρ_{calcd} = 0.965 g cm^{-3}, μ = 6.869 mm^{-1}, T = 93 K, λ(CuKα) = 1.54187 Å, $2\theta_{max}$ = 136.5°, 86430/ 31645 reflections collected/unique (R_{int} = 0.0473), R_1 = 0.1242 ($I > 2\sigma(I)$), wR_2 = 0.4029 (for all data), GOF = 1.432, largest diff. peak and hole 2.994/–1.649 eÅ^{-3}. CCDC deposit number 2223905

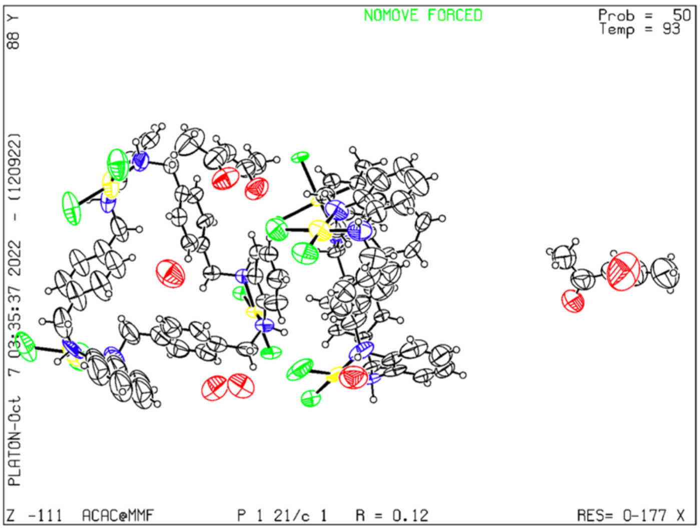

Supplementary Fig. 27 ORTEP drawing of the structurally extended MMF soaked in **ACAC** at the 50% probability level. Color: C black, N blue, O red, Cl green and Pd yellow. This figure was produced by the checkCIF report of the International Union of Crystallography.

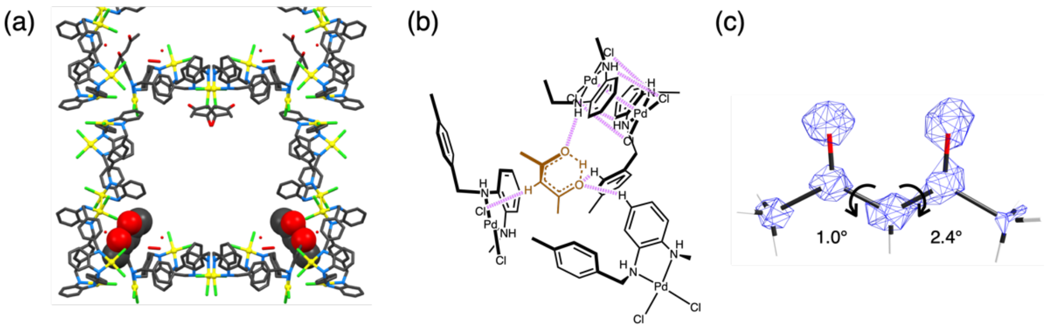

Supplementary Fig. 28 (a) The unit-space structure of the MMF that accommodates ACAC as an effector. ACAC shown in the CPK model was adsorbed in a planar configuration, indicating its enol-form. (b) Interaction patterns around the allosteric site of MMF and ACAC. (c) The electron density map of ACAC (100% occupancy) adsorbed in the allosteric binding site (contour level; 2.4 eÅ^{-3}) with the dihedral angle.

•Cyclohexanone

Soaking procedure: As-crystalised MMF crystals were firstly soaked in dichloromethane in order to remove acetonitrile from the supernatant. Afterwards, the crystals were transferred into cyclohexanone to be soaked for 3 days at 20 °C, and one of the crystals was picked up and immediately mixed with paratone oil to measure single-crystal XRD.

Crystal data for $(Pd_3LCl_6)_2\cdot$(**Cyclohexanone**)$_{2.93}\cdot(H_2O)_{1.5}$: $C_{101.57}H_{113.28}Cl_{12}N_{12}O_{4.43}Pd_6$, F_w = 2636.80, crystal dimensions 0.19 × 0.10 × 0.05 mm^3, monoclinic, space group $P2_1/c$, a = 22.643(2), b = 53.321(8), c = 14.3885(6) Å, β = 98.993(7)°, V = 17158(3) Å^3, Z = 4, ρ_{calcd} = 1.021 g cm^{-3}, μ = 6.964 mm^{-1}, T = 93 K, λ(CuKα) = 1.54187 Å, $2\theta_{max}$ = 136.5°, 72612/ 30897 reflections collected/unique (R_{int} = 0.0802), R_1 = 0.1274 ($I > 2\sigma(I)$), wR_2 = 0.3845 (for all data), GOF = 1.356, largest diff. peak and hole 2.132/–1.517 eÅ^{-3}. CCDC deposit number 2223906

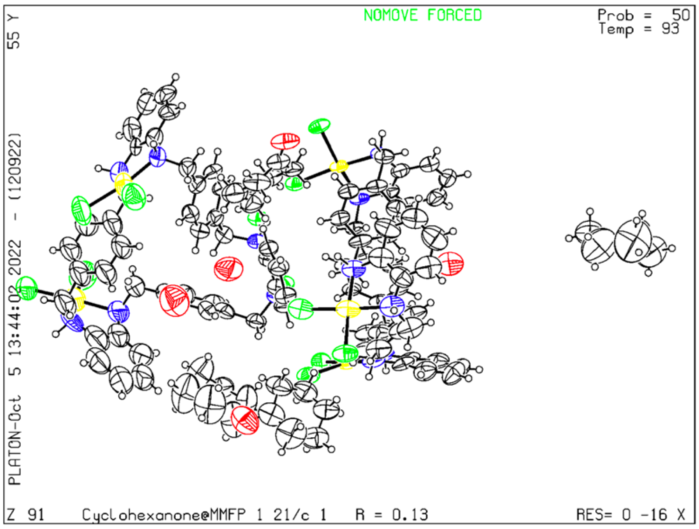

Supplementary Fig. 29 ORTEP drawing of the structurally extended MMF soaked in **cyclohexanone** at the 50% probability level. Color: C black, N blue, O red, Cl green and Pd yellow. This figure was produced by the checkCIF report of the International Union of Crystallography.

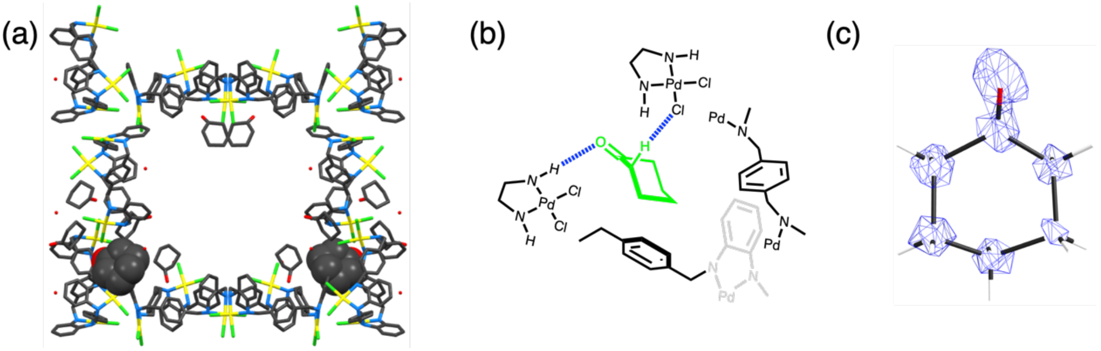

Supplementary Fig. 30 (a) The unit-space structure of the MMF that accommodates cyclohexanone shown in the CPK model as an effector. (b) Interaction patterns around the allosteric site of MMF and cyclohexanone. (c) The electron density map of cyclohexanone (100% occupancy) adsorbed in the allosteric binding site (contour level; 2.5 e$Å^{-3}$).

•Cyclohexanol

Soaking procedure: As-crystalised MMF crystals were firstly soaked in dichloromethane in order to remove acetonitrile from the supernatant. Afterwards, the crystals were transferred into cyclohexanol to be soaked at 30 °C for 1 day, and one of the crystals was picked up and immediately mixed with paratone oil to measure single-crystal XRD.

Crystal data for $(Pd_3\mathbf{L}Cl_6)_2$·(**cyclohexanol**)$_{1.68}$·$(H_2O)_{1.32}$: $C_{94.11}H_{102.53}Cl_{12}N_{12}O_3Pd_6$, F_w = 2513.50, crystal dimensions 0.16 × 0.07 × 0.07 mm^3, monoclinic, space group $P2_1/c$, a = 22.9771(5), b = 53.1007(12), c = 14.4759(2) Å, β = 98.8068(17)°, V = 17453.8(6) $Å^3$, Z = 4, ρ_{calcd} = 0.957 g cm^{-3}, μ = 6.820 mm^{-1}, T = 93 K,

λ(CuKα) = 1.54187 Å, $2\theta_{max}$ = 136.5°, 92577/ 31841 reflections collected/unique (R_{int} = 0.0576), R_1 = 0.1167 ($I > 2\sigma(I)$), wR_2 = 0.3897 (for all data), GOF = 1.364, largest diff. peak and hole 1.810/–1.785 eÅ^{-3}. CCDC deposit number 2223907

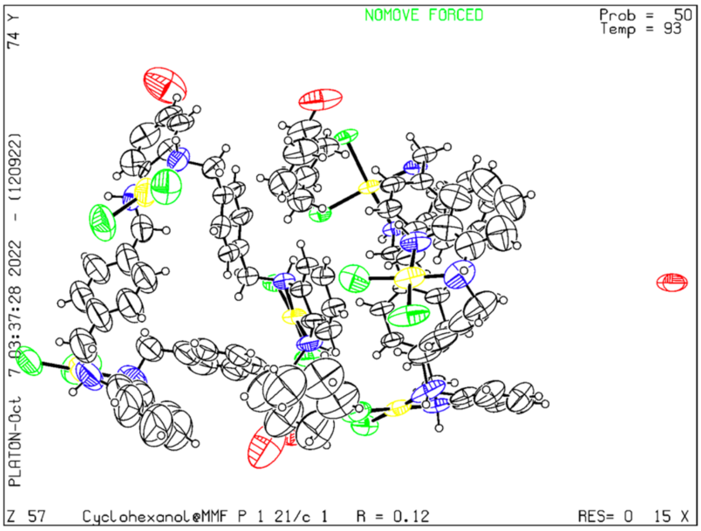

Supplementary Fig. 31 ORTEP drawing of the structurally extended MMF soaked in **cyclohexanol** at the 50% probability level. Color: C black, N blue, O red, Cl green and Pd yellow. This figure was produced by the checkCIF report of the International Union of Crystallography.

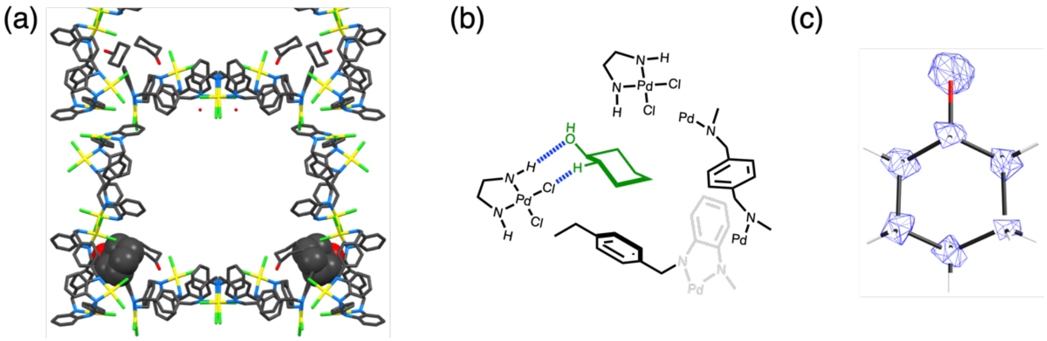

Supplementary Fig. 32 (a) The unit-space structure of the MMF that accommodates cyclohexanol shown in the CPK model as an effector. (b) Interaction patterns around the allosteric site of MMF and cyclohexanol. (c) The electron density map of cyclohexanol (100% occupancy) adsorbed in the allosteric binding site (contour level; 2.2 eÅ^{-3}).

•1,4-Butanediol (BD)

Soaking procedure: As-crystalised MMF crystals were firstly soaked in dichloromethane in order to remove acetonitrile from the supernatant. Afterwards, the crystals were transferred into 1,4-butanediol (BD) to be soaked at 20 °C for 1 day, and one of the crystals was picked up and immediately mixed with paratone oil to measure single-crystal XRD.

Crystal data for $(Pd_3\mathbf{L}Cl_6)_2\cdot(\mathbf{BD})_{2.07}\cdot(H_2O)_{1.5}$: $C_{91.75}H_{99.50}Cl_{12}N_{12}O_{5.38}Pd_6$, F_w = 2520.13, crystal dimensions 0.22 × 0.09 × 0.06 mm^3, monoclinic, space group $P2_1/c$, a = 22.5848(5), b = 53.1102(10), c = 14.41380(17) Å, β = 97.5315(15)°, V = 17140.0(5) Å^3, Z = 4, ρ_{calcd} = 0.977 g cm^{-3}, μ = 6.957 mm^{-1}, T = 93 K, λ(CuKα) = 1.54187 Å, $2\theta_{max}$ = 136.5°, 71818/30870 reflections collected/unique (R_{int} = 0.0434), R_1 = 0.1210 ($I > 2\sigma(I)$), wR_2 = 0.3843 (for all data), GOF = 1.398, largest diff. peak and hole 2.398/–3.173 eÅ^{-3}. CCDC deposit number 2223908

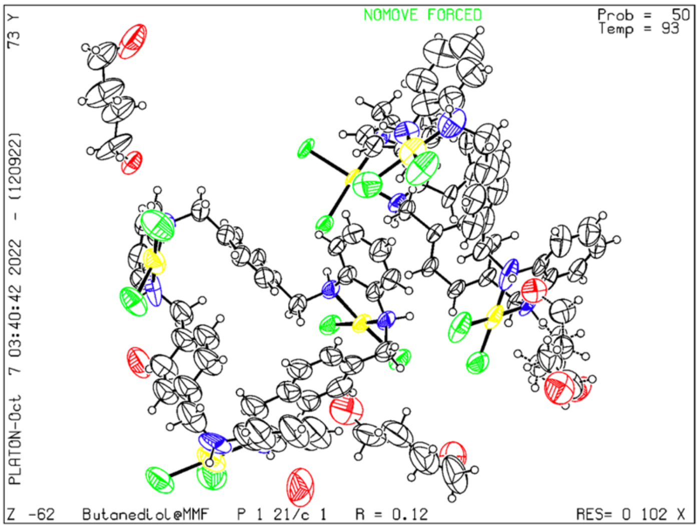

Supplementary Fig. 33 ORTEP drawing of the structurally extended MMF soaked in **BD** at the 50% probability level. Color: C black, N blue, O red, Cl green and Pd yellow. This figure was produced by the checkCIF report of the International Union of Crystallography.

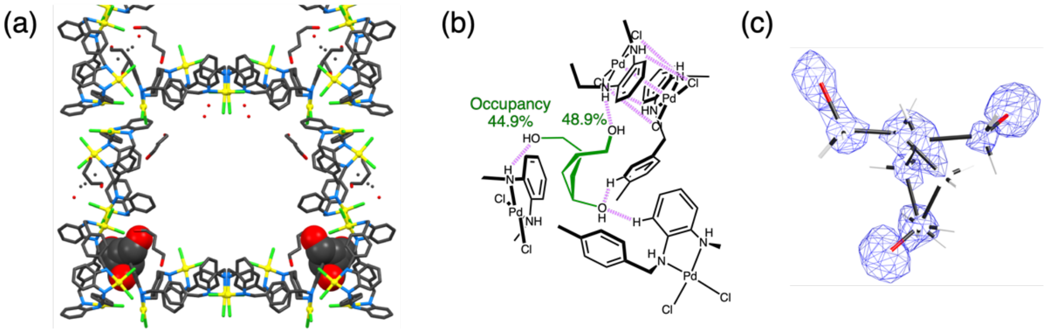

Supplementary Fig. 34 (a) The unit-space structure of the MMF that accommodates BD shown in the CPK model as an effector. (b) Interaction patterns around the allosteric site of MMF and BD, adsorbed in two different conformations. (c) The electron density map of BD (93.8% occupancy in total) adsorbed in the allosteric binding site (contour level; 1.3 eÅ^{-3}).

•Glycerol

Soaking procedure: As-crystalised MMF crystals were firstly soaked in dichloromethane in order to remove acetonitrile from the supernatant. Afterwards, the crystals were transferred into glycerol to be soaked for 1 day at room temperature, and one of the crystals was picked up to measure single-crystal XRD.

Crystal data for $(Pd_3\mathbf{L}Cl_6)_2\cdot$(**Glycerol**)$_{2.38}\cdot(H_2O)_6$: $C_{91.16}H_{95.94}Cl_{12}N_{12}O_{13.16}Pd_6$, F_w = 2634.08, crystal dimensions 0.18 × 0.14 × 0.05 mm^3, monoclinic, space group $P2_1/c$, a = 22.6627(3), b = 53.4270(6), c = 14.19443(11) Å, β = 95.5165(9)°, V = 17107.0(3) Å^3, Z = 4, ρ_{calcd} = 1.023 g cm^{-3}, μ = 7.024 mm^{-1}, T = 103 K, λ(CuKα) = 1.54187 Å, $2\theta_{max}$ = 136.5°, 177737/34111 reflections collected/unique (R_{int} = 0.0653), R_1 = 0.0932 ($I > 2\sigma(I)$), wR_2 = 0.3078 (for all data), GOF = 1.134, largest diff. peak and hole 1.868/–1.395 eÅ^{-3}. CCDC deposit number 2223909

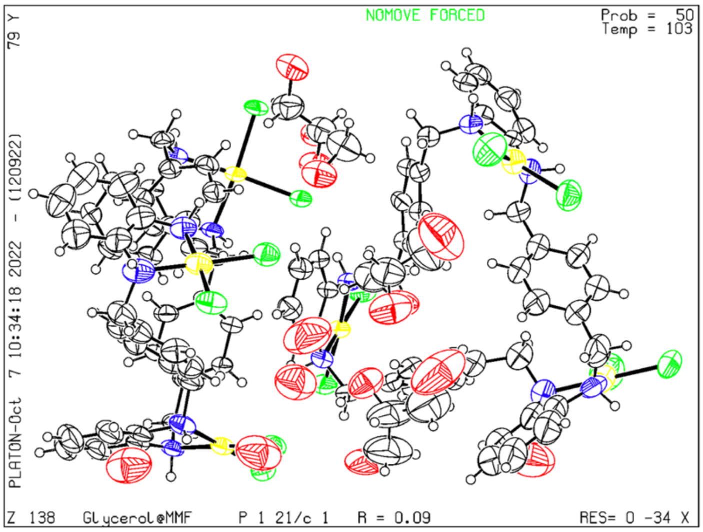

Supplementary Fig. 35 ORTEP drawing of the structurally extended MMF soaked in **glycerol** at the 50% probability level. Color: C black, N blue, O red, Cl green and Pd yellow. This figure was produced by the checkCIF report of the International Union of Crystallography.

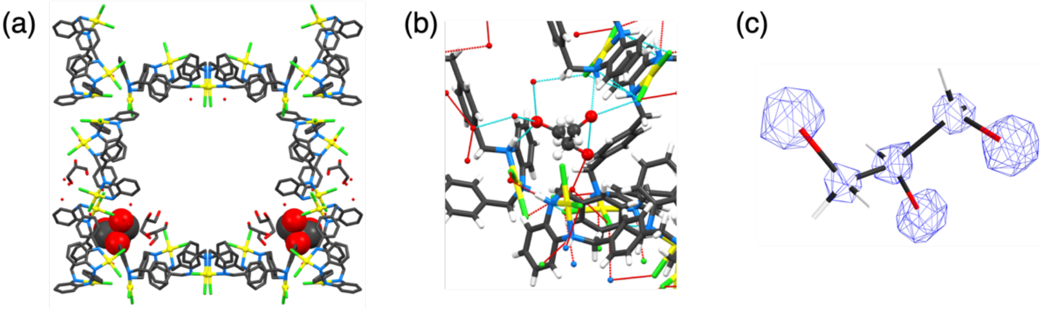

Supplementary Fig. 36 (a) The unit-space structure of the MMF that accommodates glycerol shown in the CPK model, as an effector. (b) H-bond around the allosteric site accommodating glycerol, illustrated by the ball and stick style. (c) The electron density map of glycerol (100% occupancy) adsorbed in the allosteric binding site (contour level; 2.5 eÅ^{-3}).

•2-methyl-1,3-propanediol (MPD)

Soaking procedure: As-crystalised MMF crystals were firstly soaked in dichloromethane in order to remove acetonitrile from the supernatant. Afterwards, the crystals were transferred into 2-methyl-1,3-propanediol

(MPD) to be soaked at room temperature for 1 day, and one of the crystals was picked up to measure single-crystal XRD.

Crystal data for $(Pd_3\mathbf{L}Cl_6)_2\cdot(\mathbf{MPD})_{1.79}\cdot(H_2O)_3$: $C_{90.70}H_{96.41}Cl_{12}N_{12}O_{6.60}Pd_6$, F_w = 2524.10, crystal dimensions 0.19 × 0.10 × 0.05 mm^3, monoclinic, space group $P2_1/c$, a = 22.4806(2), b = 52.6803(8), c = 14.4738(1) Å, β = 97.212(1)°, V = 17005.5(3) $Å^3$, Z = 4, ρ_{calcd} = 0.986 g cm^{-3}, μ = 7.018 mm^{-1}, T = 103 K, λ(CuKα) = 1.54187 Å, $2\theta_{max}$ = 136.5°, 183512/33906 reflections collected/unique (R_{int} = 0.0743), R_1 = 0.1085 ($I > 2\sigma(I)$), wR_2 = 0.3465 (for all data), GOF = 1.056, largest diff. peak and hole 1.441/–1.582 e$Å^{-3}$. CCDC deposit number 2223910

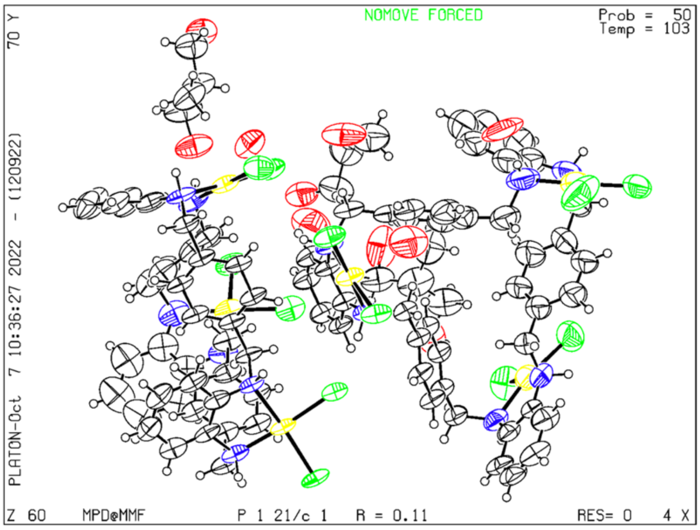

Supplementary Fig. 37 ORTEP drawing of the structurally extended MMF soaked in **MPD** at the 50% probability level. Color: C black, N blue, O red, Cl green and Pd yellow. This figure was produced by the checkCIF report of the International Union of Crystallography.

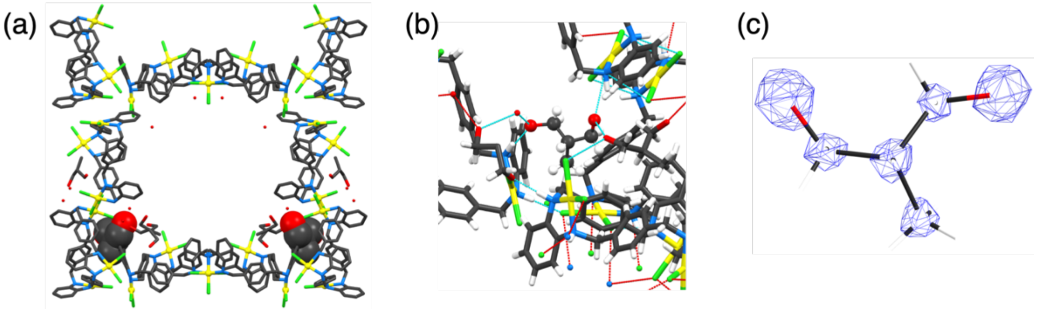

Supplementary Fig. 38 (a) The unit-space structure of the MMF that accommodates MPD shown in the CPK model, as an effector. (b) H-bond around the allosteric site accommodating MPD, illustrated by ball and stick style. (c) The electron density map of MPD (96.1% occupancy) adsorbed in the allosteric binding site (contour level; 2.0 e$Å^{-3}$).

•1,1-Bis(hydroxymethyl)cyclopropane (BHC)

Soaking procedure: As-crystalised MMF crystals were firstly soaked in dichloromethane in order to remove

acetonitrile from the supernatant. Afterwards, the crystals were transferred into 1,1-bis(hydroxymethyl)cyclopropane (BHC) to be soaked for 1 day at room temperature, and one of the crystals was picked up to measure single-crystal XRD.

Crystal data for $(Pd_3\mathbf{L}Cl_6)_2{\cdot}(\mathbf{BHC})_{1.6}{\cdot}(H_2O)_{2.24}$: $C_{91.88}H_{96.60}Cl_{12}N_{12}O_{5.40}Pd_6$, $F_w = 2519.10$, crystal dimensions $0.17 \times 0.09 \times 0.04$ mm^3, monoclinic, space group $P2_1/c$, $a = 23.6608(6)$, $b = 52.6360(13)$, $c = 14.4077(2)$ Å, $\beta = 97.8950(19)°$, $V = 17773.4(7)$ Å^3, $Z = 4$, $\rho_{calcd} = 0.941$ g cm^{-3}, $\mu = 6.709$ mm^{-1}, $T = 103$ K, $\lambda(CuK\alpha) = 1.54187$ Å, $2\theta_{max} = 136.5°$, 95874/34830 reflections collected/unique ($R_{int} = 0.0931$), $R_1 = 0.1249$ ($I > 2\sigma(I)$), $wR_2 = 0.4062$ (for all data), GOF = 1.246, largest diff. peak and hole 1.635/–1.121 eÅ^{-3}. CCDC deposit number 2223911

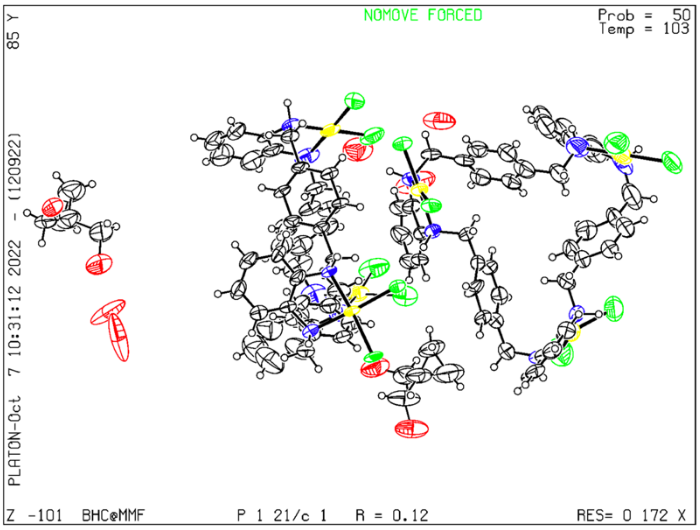

Supplementary Fig. 39 ORTEP drawing of the structurally extended MMF soaked in **BHC** at the 50% probability level. Color: C black, N blue, O red, Cl green and Pd yellow. This figure was produced by the checkCIF report of the International Union of Crystallography.

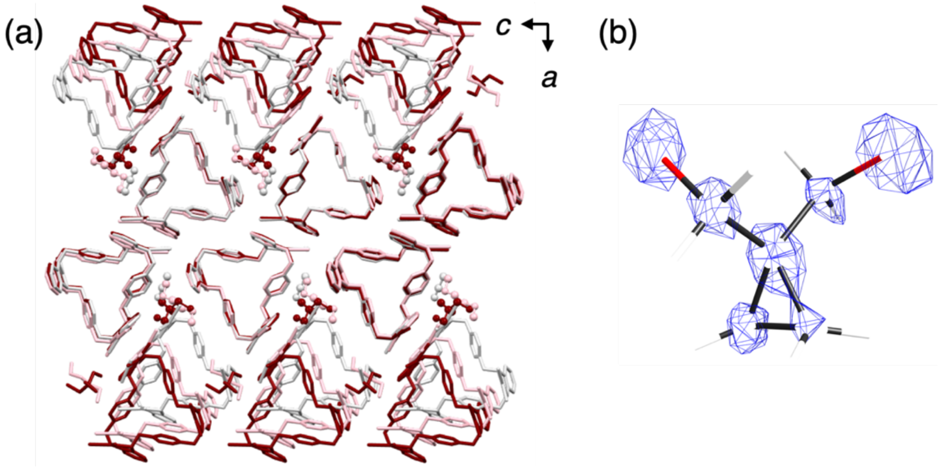

Supplementary Fig. 40 (a) Structural overlap of the two-dimensional network of MMF *syn*-Pd-macrocycles: the as-crystalised MMF in acetonitrile (grey), the extended MMF in DME (light pink), and the extended MMF in BHC (dark red). (b) The electron density map of BHC (83.4% occupancy) adsorbed in the allosteric binding

site (contour level; 1.8 eÅ^{-3}).

•4-Hydroxytetrahydropyran (Pyranol)

Soaking procedure: As-crystalised MMF crystals were firstly soaked in dichloromethane in order to remove acetonitrile from the supernatant. Afterwards, the crystals were transferred into 4-hydroxytetrahydropyran (pyranol) to be soaked at room temperature for 6 h, and one of the crystals was picked up and immediately mixed with fluorolube® to measure single-crystal XRD.

Crystal data for $(Pd_3\mathbf{L}Cl_6)_2\cdot(\mathbf{Pyranol})_{1.85}\cdot(H_2O)_{1.2}$: $C_{93.31}H_{100.75}Cl_{12}N_{12}O_{4.92}Pd_6$, F_w = 2532.74, crystal dimensions 0.13 × 0.07 × 0.03 mm^3, monoclinic, space group $P2_1/c$, a = 22.8699(7), b = 53.1568(15), c = 14.4932(3) Å, β = 98.578(2)°, V = 17422.2(8) Å^3, Z = 4, ρ_{calcd} = 0.966 g cm^{-3}, μ = 6.844 mm^{-1}, T = 103 K, λ(CuKα) = 1.54187 Å, $2\theta_{max}$ = 136.5°, 76641/33671 reflections collected/unique (R_{int} = 0.0864), R_1 = 0.1179 ($I > 2\sigma(I)$), wR_2 = 0.3882 (for all data), GOF = 1.019, largest diff. peak and hole 1.510/–0.743 eÅ^{-3}. CCDC deposit number 2223912

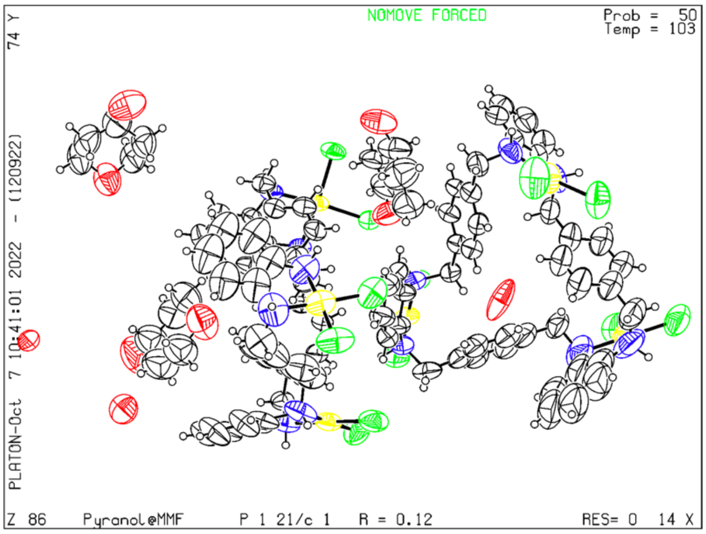

Supplementary Fig. 41 ORTEP drawing of the structurally extended MMF soaked in **pyranol** at the 50% probability level. Color: C black, N blue, O red, Cl green and Pd yellow. This figure was produced by the checkCIF report of the International Union of Crystallography.

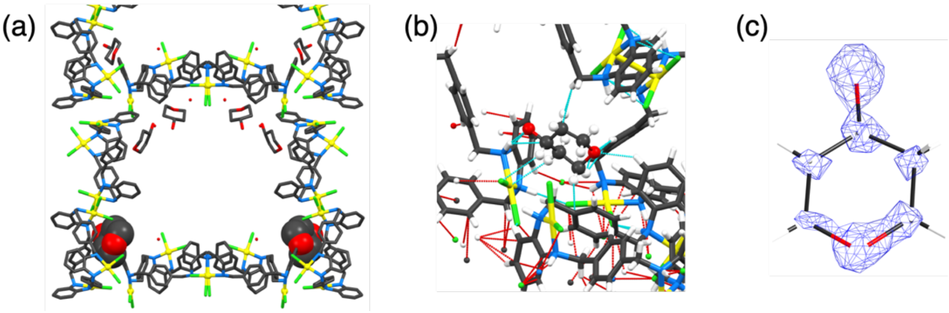

Supplementary Fig. 42 (a) The unit-space structure of the MMF accommodates pyranol shown in the CPK

model, as an effector. (b) Short contact around the allosteric site accommodating pyranol, illustrated by the ball and stick style. (c) The electron density map of pyranol (80.0% occupancy) adsorbed in the allosteric binding site (contour level; 1.6 eÅ^{-3}).

•Acetophenone (ACP)

Soaking procedure: As-crystalised MMF crystals were first soaked in DME in order to induce the lattice expansion at room temperature. Afterwards, the crystals were transferred into acetophenone (ACP) to be soaked at 20 °C for 1 day, and one of the crystals was picked up and immediately mixed with paratone oil to measure single-crystal XRD.

Crystal data for $(Pd_3\mathbf{L}Cl_6)_2\cdot(\mathbf{ACP})_{1.44}\cdot(H_2O)_{1.5}$: $C_{95.53}H_{95.53}Cl_{12}N_{12}O_{2.94}Pd_6$, F_w = 2522.53, crystal dimensions 0.26 × 0.11 × 0.09 mm^3, monoclinic, space group $P2_1/c$, a = 22.6679(2), b = 53.6667(6), c = 14.41850(11) Å, β = 96.8952(7)°, V = 17413.4(3) Å^3, Z = 4, ρ_{calcd} = 0.962 g cm^{-3}, μ = 6.838 mm^{-1}, T = 93 K, λ(CuKα) = 1.54187 Å, $2\theta_{max}$ = 136.5°, 98875/31708 reflections collected/unique (R_{int} = 0.0349), R_1 = 0.1247 ($I > 2\sigma(I)$), wR_2 = 0.4094 (for all data), GOF = 1.717, largest diff. peak and hole 3.347/–2.844 eÅ^{-3}. CCDC deposit number 2223913

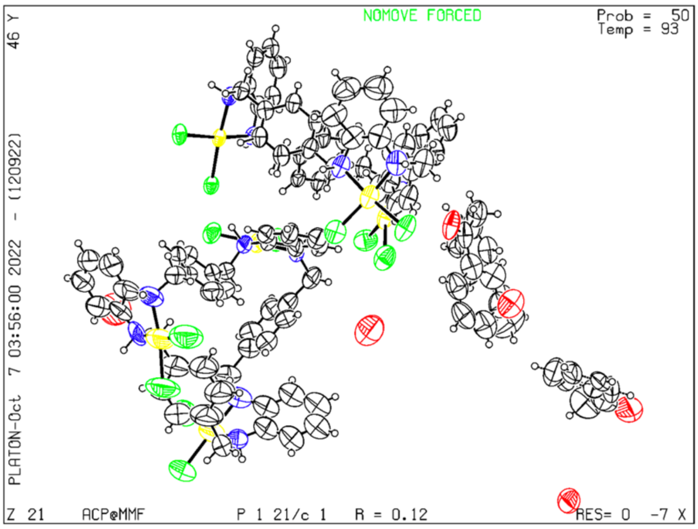

Supplementary Fig. 43 ORTEP drawing of the structurally extended MMF soaked in **ACP** at the 50% probability level. Color: C black, N blue, O red, Cl green and Pd yellow. This figure was produced by the checkCIF report of the International Union of Crystallography.

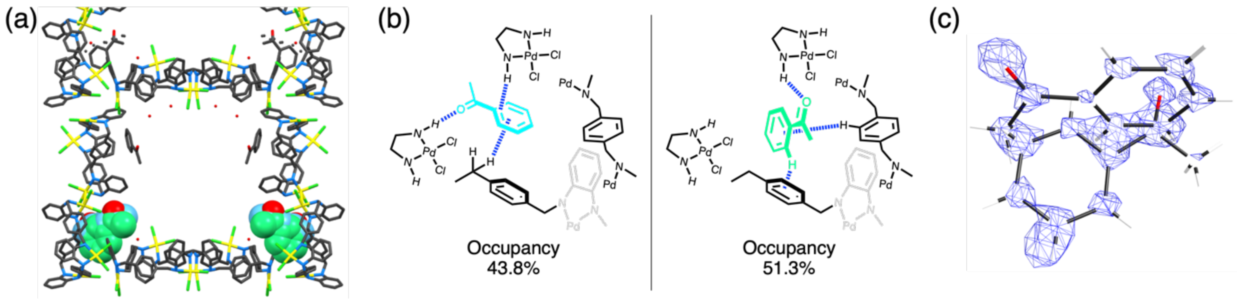

Supplementary Fig. 44 (a) The unit-space structure of the MMF accommodates ACP shown in the CPK model, as an effector. ACP has two different conformations in the allosteric site whose C-atoms are colored by light blue and light green, respectively. (b) Interaction patterns around the allosteric site of MMF and ACP. (c) The electron density map of ACP (95.1% occupancy in total) adsorbed in the allosteric binding site (contour level; 1.2 eÅ^{-3}).

•Benzonitrile (PhCN)

Soaking procedure: As-crystalised MMF crystals were firstly soaked in DME in order to induce the lattice expansion at room temperature. Afterwards, the crystals were transferred into benzonitrile (PhCN) to be soaked at 20 °C for 3 days, and one of the crystals was picked up and immediately mixed with paratone oil to measure single-crystal XRD.

Crystal data for $(Pd_3\mathbf{L}Cl_6)_2\cdot(\mathbf{PhCN})_{3.75}$: $C_{109.75}H_{100.25}Cl_{12}N_{15.25}Pd_6$, F_w = 2696.60, crystal dimensions 0.21 × 0.06 × 0.05 mm^3, monoclinic, space group $P2_1/c$, a = 22.56030(17), b = 51.4719(8), c = 14.52170(11) Å, β = 96.9786(7)°, V = 16738.0(3) Å^3, Z = 4, ρ_{calcd} = 1.070 g cm^{-3}, μ = 7.137 mm^{-1}, T = 93 K, λ(CuKα) = 1.54187 Å, $2\theta_{max}$ = 136.5°, 83650/30535 reflections collected/unique (R_{int} = 0.0415), R_1 = 0.1053 ($I > 2\sigma(I)$), wR_2 = 0.3481 (for all data), GOF = 1.381, largest diff. peak and hole 3.485/–2.938 eÅ^{-3}. CCDC deposit number 2223914

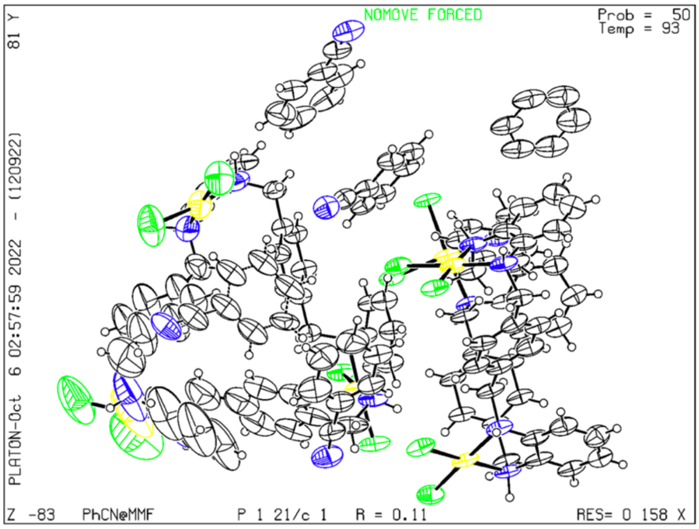

Supplementary Fig. 45 ORTEP drawing of the structurally extended MMF soaked in **PhCN** at the 50% probability level. Color: C black, N blue, O red, Cl green and Pd yellow. This figure was produced by the checkCIF report of the International Union of Crystallography.

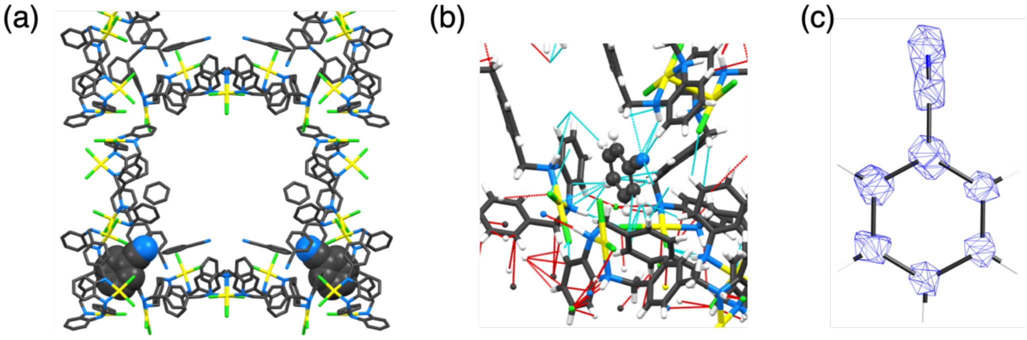

Supplementary Fig. 46 (a) The unit-space structure of the MMF that accommodates PhCN as shown in the CPK model, as an effector. (b) Short contact around the allosteric site accommodating PhCN, illustrated by the ball and stick style. (c) The electron density map of PhCN (100% occupancy) adsorbed in the allosteric binding site (contour level; 3.0 eÅ^{-3}).

•Nitrobenzene (NB)

Soaking procedure: As-crystalised MMF crystals were firstly soaked in dichloromethane in order to remove acetonitrile from the supernatant. Afterwards, the crystals were transferred into nitrobenzene (NB) to be soaked at 70 °C for 1 day, and one of the crystals was picked up and immediately mixed with paratone oil to measure single-crystal XRD.

Crystal data for $(Pd_3\mathbf{L}Cl_6)_2\cdot(\mathbf{NB})_1\cdot(H_2O)_{1.65}$: $C_{90}H_{89}Cl_{12}N_{13}O_{3.65}Pd_6$, F_w = 2474.94, crystal dimensions 0.09 × 0.05 × 0.04 mm^3, monoclinic, space group $P2_1/c$, a = 22.7158(10), b = 53.189(3), c = 14.4006(3) Å, β = 97.063(3)°, V = 17267.2(13) Å^3, Z = 4, ρ_{calcd} = 0.952 g cm^{-3}, μ = 6.894 mm^{-1}, T = 93 K, λ(CuKα) = 1.54187 Å, $2\theta_{max}$ = 136.5°, 67039/30843 reflections collected/unique (R_{int} = 0.0660), R_1 = 0.1505 ($I > 2\sigma(I)$), wR_2 = 0.4495 (for all data), GOF = 1.360, largest diff. peak and hole 1.842/–1.382 eÅ^{-3}. CCDC deposit number 2223915

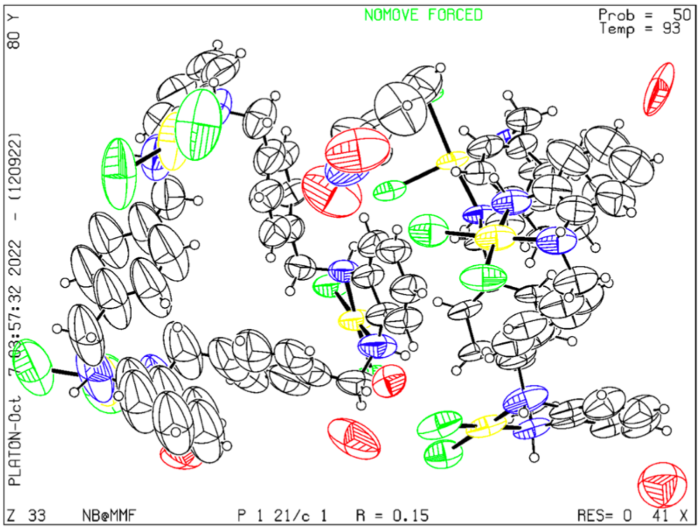

Supplementary Fig. 47 ORTEP drawing of the structurally extended MMF soaked in **NB** at the 50% probability

level. Color: C black, N blue, O red, Cl green and Pd yellow. This figure was produced by the checkCIF report of the International Union of Crystallography.

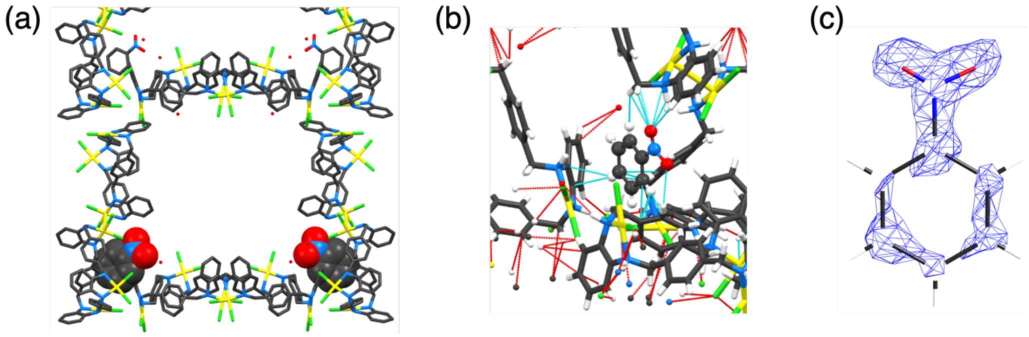

Supplementary Fig. 48 (a) The unit-space structure of the MMF that accommodates NB shown in the CPK model, as an effector. (b) Short contact around the allosteric site accommodating NB, illustrated by the ball and stick style. (c) The electron density map of NB (100% occupancy) adsorbed in the allosteric binding site (contour level; 1.7 eÅ^{-3})

•Furfuryl alcohol (FA)

Soaking procedure: As-crystalised MMF crystals were firstly soaked in DME in order to induce the lattice expansion at room temperature. Afterwards, the crystals were transferred into furfuryl alcohol (FA) to be soaked at 20 °C for 1 h, and one of the crystals was picked up and immediately mixed with paratone oil to measure single-crystal XRD.

Crystal data for $(Pd_3\mathbf{L}Cl_6)_2\cdot(\mathbf{FA})_{2.7}\cdot(H_2O)_2$: $C_{100.41}H_{97.49}Cl_{12}N_{12}O_{8.12}Pd_6$, F_w = 2666.08, crystal dimensions 0.18 × 0.09 × 0.08 mm^3, monoclinic, space group $P2_1/c$, a = 22.7094(3), b = 52.2820(11), c = 14.37320(16) Å, β = 97.2932(12)°, V = 16927.1(5) Å^3, Z = 4, ρ_{calcd} = 1.046 g cm^{-3}, μ = 7.083 mm^{-1}, T = 93 K, λ(CuKα) = 1.54187 Å, $2\theta_{max}$ = 136.5°, 184931/31005 reflections collected/unique (R_{int} = 0.0696), R_1 = 0.1283 ($I > 2\sigma(I)$), wR_2 = 0.3652 (for all data), GOF = 1.124, largest diff. peak and hole 2.516/–2.331 eÅ^{-3}. CCDC deposit number 2223916

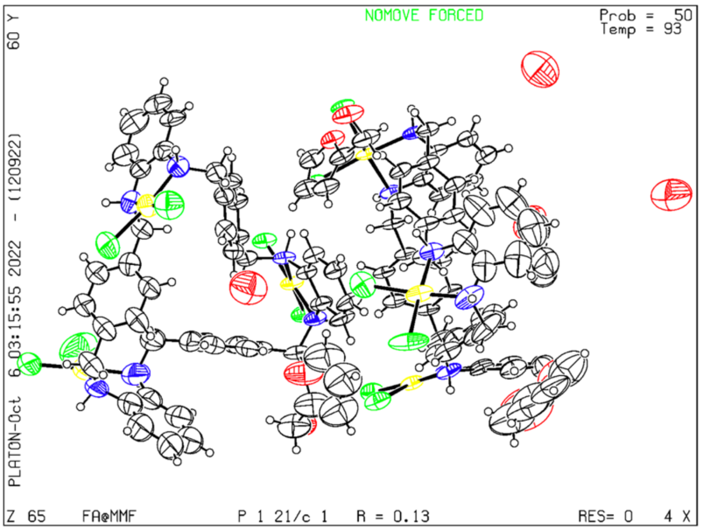

Supplementary Fig. 49 ORTEP drawing of the structurally extended MMF soaked in **FA** at the 50% probability level. Color: C black, N blue, O red, Cl green and Pd yellow. This figure was produced by the checkCIF report of the International Union of Crystallography.

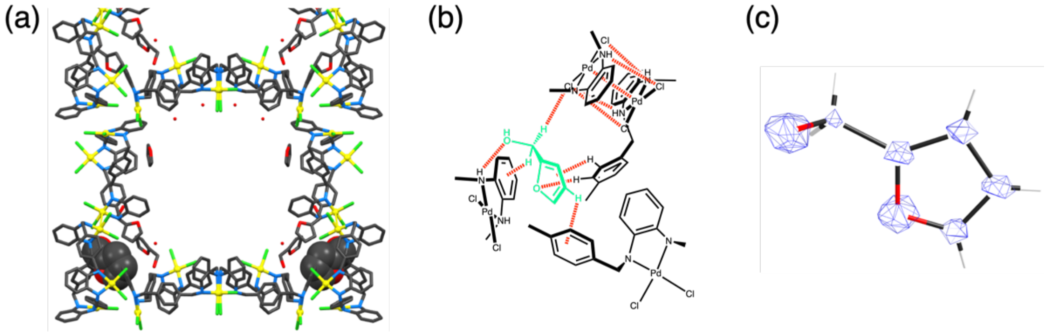

Supplementary Fig. 50 (a) The unit-space structure of the MMF that accommodates FA shown in the CPK model, as an effector. (b) Interaction patterns around the allosteric site of MMF and FA. (c) The electron density map of FA (97.6% occupancy) adsorbed in the allosteric binding site (contour level; 3.8 eÅ^{-3}).

•Resorcinol

Soaking procedure: As-crystalised MMF crystals were firstly soaked in dichloromethane in order to remove acetonitrile from the supernatant. Afterwards, the crystals were transferred into 1,2,3,4-tetrahydronaphthalene:acetone = 9:1 (v:v) solution of resorcinol (500 mM) to be soaked at 20 °C for 1 day, and one of the crystals was picked up and immediately mixed with paratone oil to measure single-crystal XRD.

Crystal data for $(Pd_3\mathbf{L}Cl_6)_2\cdot(\mathbf{resorcinol})_{1.4}\cdot(H_2O)_1$: $C_{92.40}H_{89.60}Cl_{12}N_{12}O_{3.80}Pd_6$, F_w = 2492.76, crystal dimensions 0.14 × 0.10 × 0.05 mm^3, monoclinic, space group $P2_1/c$, a = 22.5272(4), b = 52.9226(13), c = 14.4948(2) Å, β = 96.7202(14)°, V = 17161.9(6) Å^3, Z = 4, ρ_{calcd} = 0.965 g cm^{-3}, μ = 6.938 mm^{-1}, T = 93 K, λ(CuKα) = 1.54187 Å, $2\theta_{max}$ = 136.5°, 97552/31144 reflections collected/unique (R_{int} = 0.0713), R_1 = 0.1160 ($I > 2\sigma(I)$), wR_2 =

0.3771 (for all data), GOF = 1.220, largest diff. peak and hole 1.696/–1.511 eÅ^{-3}. CCDC deposit number 2223917

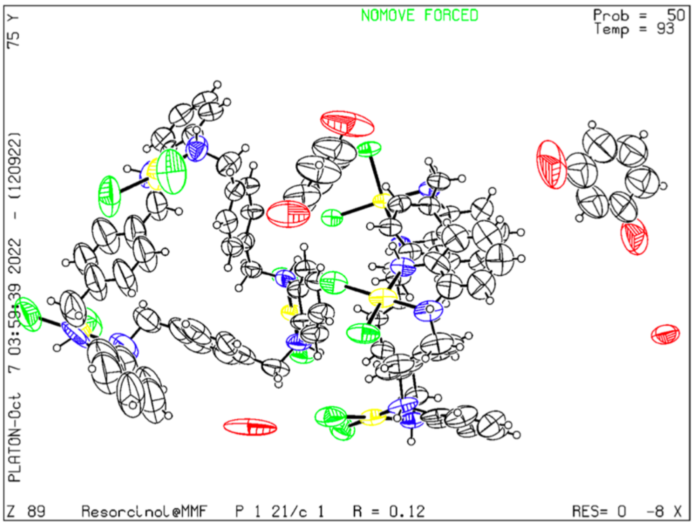

Supplementary Fig. 51 ORTEP drawing of the structurally extended MMF soaked in 1,2,3,4-tetrahydronaphthalene:acetone = 9:1 (v:v) solution of **resorcinol** at the 50% probability level. Color: C black, N blue, O red, Cl green and Pd yellow. This figure was produced by the checkCIF report of the International Union of Crystallography.

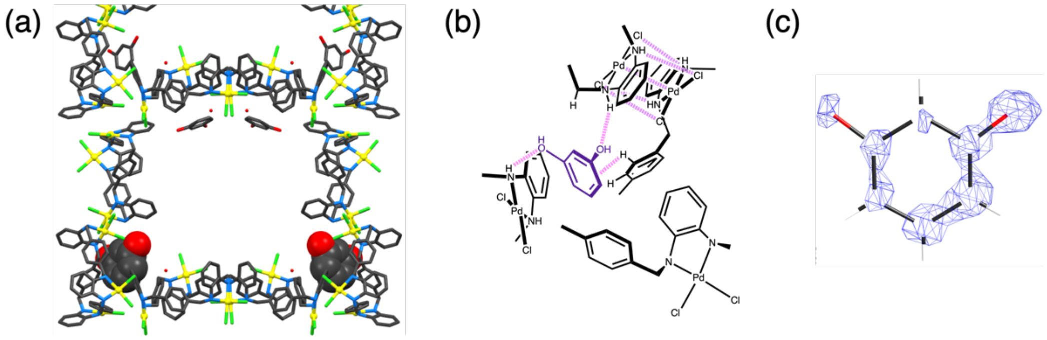

Supplementary Fig. 52 (a) The unit-space structure of the MMF that accommodates resorcinol shown in the CPK model, as an effector. (b) Interaction patterns around the allosteric site of MMF and resorcinol. (c) The electron density map of resorcinol (100% occupancy) adsorbed in the allosteric binding site (contour level; 1.5 eÅ^{-3}).

•4-Methoxyphenol (mequinol)

Soaking procedure: As-crystalised MMF crystals were firstly soaked in dichloromethane in order to remove acetonitrile from the supernatant. Afterwards, the crystals were transferred into 1,2,3,4-tetrahydronaphthalene:acetone = 9:1 (v:v) solution of 4-methoxyphenol (mequinol) (1.0 M) to be soaked at 20 °C for 1 day, and one of the crystals was picked up and immediately mixed with paratone oil to measure single-crystal XRD.

Crystal data for $(Pd_3\mathbf{L}Cl_6)_2\cdot(\mathbf{mequinol})_{0.78}\cdot(H_2O)_1$: $C_{89.46}H_{89.46}Cl_{12}N_{12}O_{2.56}Pd_6$, F_w = 2437.43, crystal dimensions 0.17 × 0.12 × 0.07 mm^3, monoclinic, space group $P2_1/c$, a = 22.4857(4), b = 52.1472(18), c = 14.50620(18) Å, β = 97.9739(14)°, V = 16845.0(7) Å^3, Z = 4, ρ_{calcd} = 0.961 g cm^{-3}, μ = 7.053 mm^{-1}, T = 93 K, λ(CuKα) = 1.54187 Å, $2\theta_{max}$ = 136.5°, 69571/30392 reflections collected/unique (R_{int} = 0.0514), R_1 = 0.1362 ($I > 2\sigma(I)$), wR_2 = 0.4176 (for all data), GOF = 1.460, largest diff. peak and hole 3.188/–3.215 eÅ^{-3}. CCDC deposit number 2223918

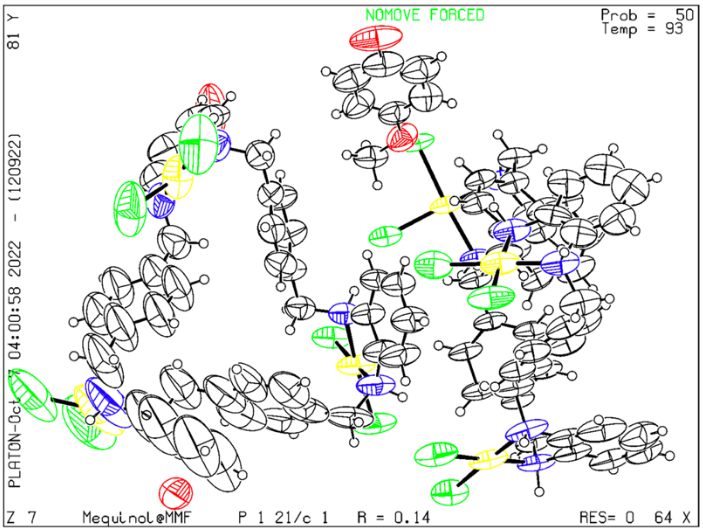

Supplementary Fig. 53 ORTEP drawing of the structurally extended MMF soaked in 1,2,3,4-tetrahydronaphthalene:acetone = 9:1 (v:v) solution of **mequinol** at the 50% probability level. Color: C black, N blue, O red, Cl green, and Pd yellow. This figure was produced by the checkCIF report of the International Union of Crystallography.

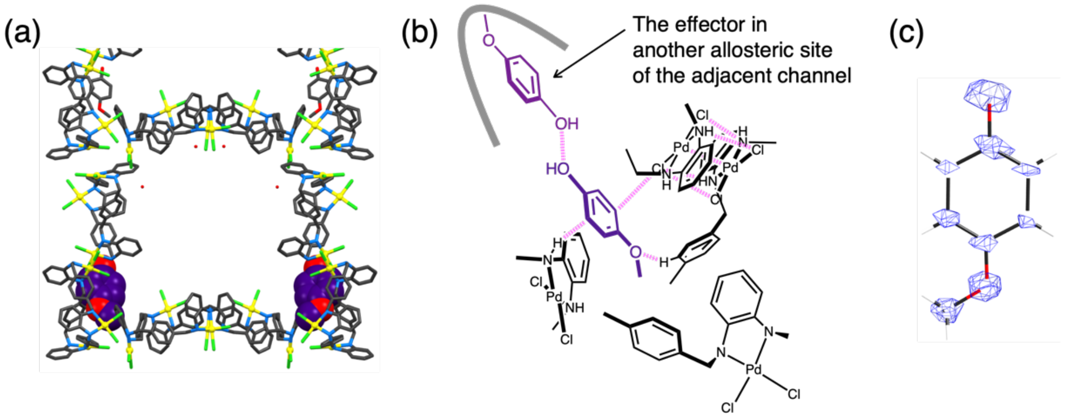

Supplementary Fig. 54 (a) The unit-space structure of that MMF that accommodates mequinol shown in the CPK model, as an effector. (b) Interaction patterns around the allosteric site of MMF and mequinol. The hydroxy group forms hydrogen bonding with the effector in another allosteric site of the adjacent channel. (c) The electron density map of mequinol (78.1% occupancy) adsorbed in the allosteric binding site. (contour level; 2.3 eÅ^{-3})

•Water

Soaking procedure: As-crystalised MMF crystals were firstly soaked in dichloromethane in order to remove acetonitrile from the supernatant. Afterwards, the crystals were transferred into water to be soaked at 20 °C for 4 h, and one of the crystals was picked up and immediately mixed with paratone oil to measure single-crystal XRD.

Crystal data for $(Pd_3\mathbf{L}Cl_6)_2\cdot(\mathbf{H_2O})_{17.5}$: $C_{84}H_{84}Cl_{12}N_{12}O_{17.50}Pd_6$, F_w = 2605.43, crystal dimensions 0.54 × 0.27 × 0.10 mm^3, monoclinic, space group $P2_1/c$, a = 19.83690(15), b = 52.8454(5), c = 14.20970(11) Å, β = 90.7011(7)°, V = 14894.8(2) Å^3, Z = 4, ρ_{calcd} = 1.162 g cm^{-3}, μ = 8.086 mm^{-1}, T = 93 K, λ(CuKα) = 1.54187 Å, $2\theta_{max}$ = 136.5°, 159965/ 27240 reflections collected/unique (R_{int} = 0.0627), R_1 = 0.0845 ($I > 2\sigma(I)$), wR_2 = 0.2521 (for all data), GOF = 1.097, largest diff. peak and hole 3.113/–2.446 eÅ^{-3}. CCDC deposit number 2223919

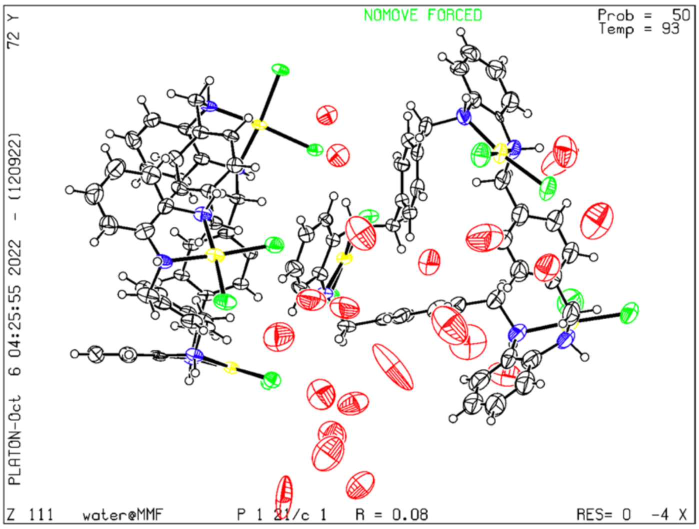

Supplementary Fig. 55 ORTEP drawing of the structurally extended MMF soaked in $\mathbf{H_2O}$ at the 50% probability level. Color: C black, N blue, O red, Cl green and Pd yellow. This figure was produced by the checkCIF report of the International Union of Crystallography.

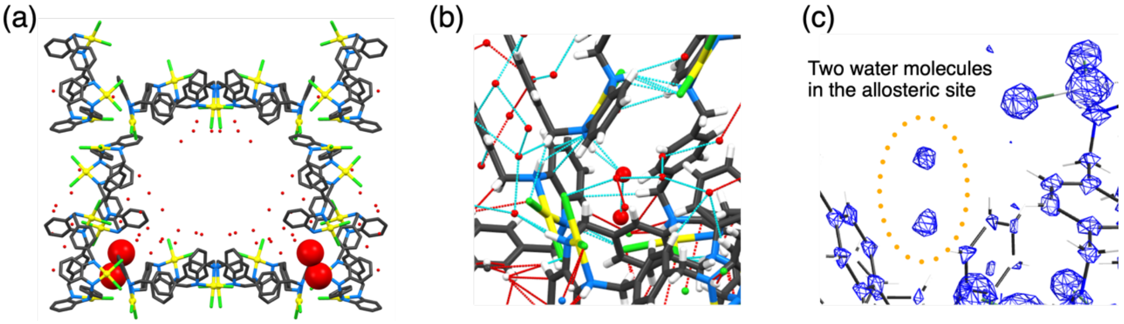

Supplementary Fig. 56 (a) The unit-space structure of the MMF that accommodates H_2O shown in the CPK model, as an effector. (b) Short contact around the allosteric site accommodating H_2O, illustrated by the ball and stick style. (c) The electron density map allosteric binding site. (contour level; 5.0 e$^-$/Å^3, Two water molecules in the allosteric site whose occupancies are 100% is highlighted by the orange dotted line.)

•Ethanol (EtOH)

Soaking procedure: As-crystalised MMF crystals were soaked in ethanol (EtOH) at 20 °C for 1 day, and one of the crystals was picked up and immediately mixed with paratone oil to measure single-crystal XRD.

Crystal data for $(Pd_3\mathbf{L}Cl_6)_2\cdot(\mathbf{EtOH})_{1.5}\cdot(H_2O)_{3.5}$: $C_{87}H_{91.50}Cl_{12}N_{12}O_5Pd_6$, F_w = 2449.02, crystal dimensions 0.50 × 0.15 × 0.11 mm^3, monoclinic, space group $P2_1/c$, a = 19.6678(2), b = 52.4980(6), c = 14.29920(11) Å, β = 90.5652(7)°, V = 14763.5(3) Å^3, Z = 4, ρ_{calcd} = 1.102 g cm^{-3}, μ = 8.062 mm^{-1}, T = 93 K, λ(CuKα) = 1.54187 Å, $2\theta_{max}$ = 136.5°, 158085/ 27008 reflections collected/unique (R_{int} = 0.0533), R_1 = 0.1174 ($I > 2\sigma(I)$), wR_2 = 0.3440 (for all data), GOF = 1.112, largest diff. peak and hole 2.623/–1.880 eÅ^{-3}. CCDC deposit number 2223920

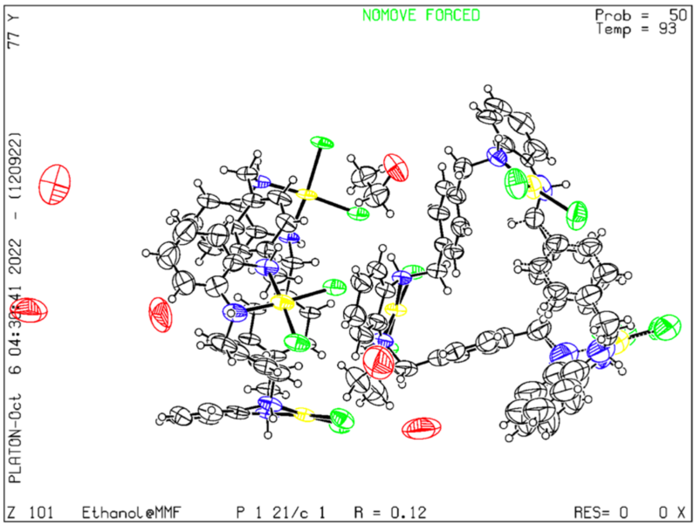

Supplementary Fig. 57 ORTEP drawing of the structurally extended MMF soaked in **EtOH** at the 50% probability level. Color: C black, N blue, O red, Cl green and Pd yellow. This figure was produced by the checkCIF report of the International Union of Crystallography.

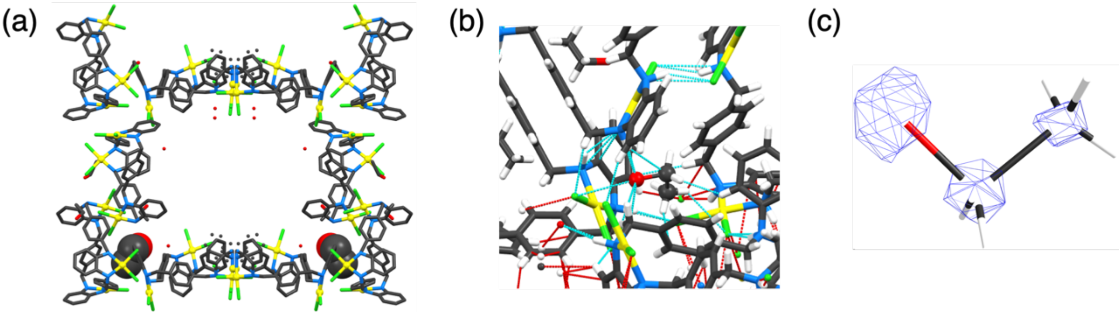

Supplementary Fig. 58 (a) Unit-space structure of MMF accommodating EtOH, illustrated by CPK model, as an effector. (b) Short contact around the allosteric site accommodating EtOH, illustrated by the ball and stick style. (c) The electron density map of EtOH (100% occupancy) adsorbed in the allosteric binding site (contour level; 2.5 eÅ^{-3}).

•Isopropanol (IPA)

Soaking procedure: As-crystalised MMF crystals were firstly soaked in dichloromethane in order to remove acetonitrile from the supernatant. Afterwards, the crystals were transferred into isopropanol (IPA) to be soaked at 20 °C for 1 day, and one of the crystals was picked up and immediately mixed with paratone oil to measure single-crystal XRD.

Crystal data for $(Pd_3\mathbf{L}Cl_6)_2\cdot(\mathbf{IPA})_{2.86}\cdot(H_2O)_{0.75}$: $C_{92.55}H_{103.95}Cl_{12}N_{12}O_{3.60}Pd_6$, $F_w = 2505.82$, crystal dimensions $0.43 \times 0.13 \times 0.08$ mm^3, monoclinic, space group $P2_1/c$, $a = 20.0430(2)$, $b = 52.8809(6)$, $c = 14.3881(1)$ Å, $\beta = 90.107(1)$°, $V = 15249.8(3)$ Å^3, $Z = 4$, $\rho_{calcd} = 1.091$ g cm^{-3}, $\mu = 7.807$ mm^{-1}, $T = 93$ K, λ(CuKα) = 1.54187 Å, $2\theta_{max} = 136.5$°, 163992/ 29764 reflections collected/unique ($R_{int} = 0.0486$), $R_1 = 0.1143$ ($I > 2\sigma(I)$), $wR_2 = 0.3289$ (for all data), GOF = 1.090, largest diff. peak and hole 3.681/–4.491 eÅ^{-3}. CCDC deposit number 2223921

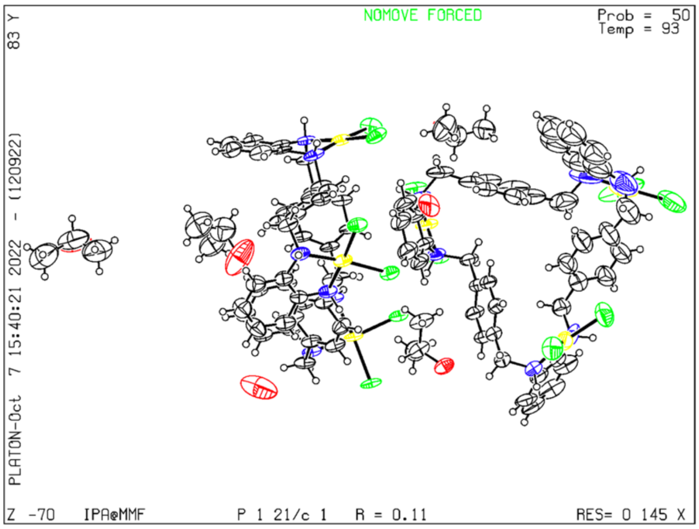

Supplementary Fig. 59 ORTEP drawing of the structurally extended MMF soaked in **IPA** at the 50% probability level. Color: C black, N blue, O red, Cl green and Pd yellow. This figure was produced by the checkCIF report of the International Union of Crystallography.

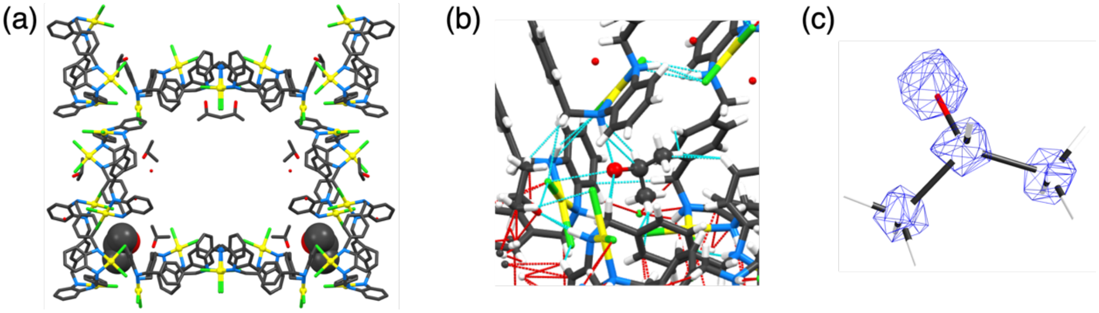

Supplementary Fig. 60 (a) The unit-space structure of the MMF that accommodates IPA shown in the CPK model, as an effector. (b) Short contact around the allosteric site accommodating IPA, illustrated by the ball and stick style. (c) The electron density map of IPA (100% occupancy) adsorbed in the allosteric binding site. (contour level; 2.5 eÅ^{-3}).

•(*rac*)-*sec*-Butyl alcohol (SBA)

Soaking procedure: As-crystalised MMF crystals were firstly soaked in dichloromethane in order to remove acetonitrile from the supernatant. Afterwards, the crystals were transferred into (*rac*)-*sec*-butyl alcohol (SBA) to be soaked at 20 °C for 4 h, and one of the crystals was picked up and immediately mixed with paratone oil to measure single-crystal XRD.

Crystal data for $(Pd_3\mathbf{L}Cl_6)_2\cdot(\mathbf{SBA})_{1.49}\cdot(H_2O)_{1.5}$: $C_{89.93}H_{97.35}Cl_{12}N_{12}O_{2.98}Pd_6$, F_w = 2457.85, crystal dimensions 0.29 × 0.20 × 0.08 mm^3, monoclinic, space group $P2_1/c$, a = 20.1631(4), b = 53.4992(5), c = 14.3264(2) Å, β = 90.286(1)°, V = 15453.8(6) Å^3, Z = 4, ρ_{calcd} = 1.056 g cm^{-3}, μ = 7.693 mm^{-1}, T = 93 K, λ(CuKα) = 1.54187 Å, $2\theta_{max}$ = 136.5°, 152519/ 29920 reflections collected/unique (R_{int} = 0.0962), R_1 = 0.1167 ($I > 2\sigma(I)$), wR_2 = 0.3357 (for all data), GOF = 1.068, largest diff. peak and hole 2.145/–2.796 eÅ^{-3}. CCDC deposit number 2223922

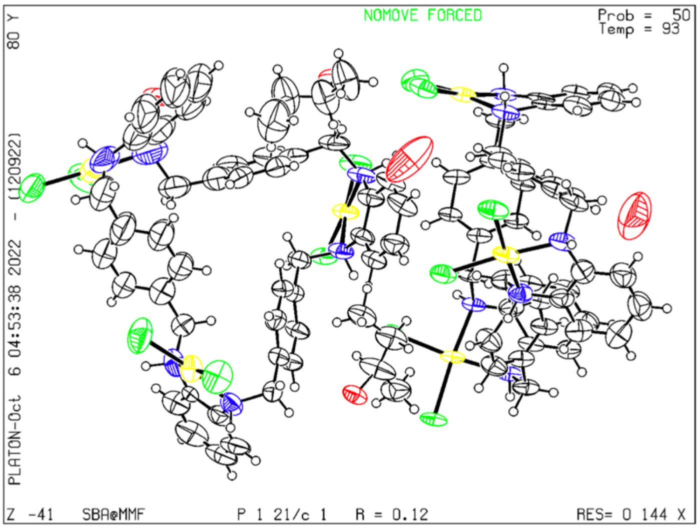

Supplementary Fig. 61 ORTEP drawing of the structurally extended MMF soaked in **SBA** at the 50% probability level. Color: C black, N blue, O red, Cl green and Pd yellow. This figure was produced by the checkCIF report of the International Union of Crystallography.

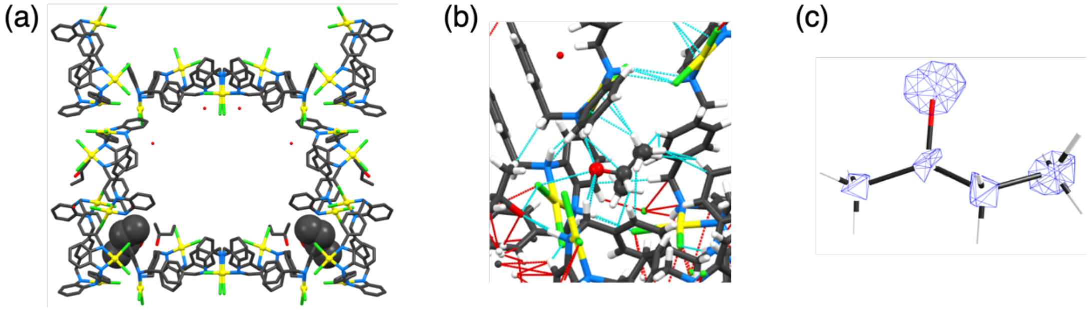

Supplementary Fig. 62 (a) The unit-space structure of the MMF that accommodates SBA shown in the CPK model, as an effector. (b) Short contact around the allosteric site accommodating SBA, illustrated by the ball and stick style. (c) The electron density map of SBA (86.3% occupancy) adsorbed in the allosteric binding site

(contour level; 2.5 eÅ^{-3}).

•(*rac*)-*sec*-Pentyl alcohol (SPA)

Soaking procedure: As-crystalised MMF crystals were firstly soaked in dichloromethane in order to remove acetonitrile from the supernatant. Afterwards, the crystals were transferred into (*rac*)-*sec*-pentyl alcohol (SPA) to be soaked at 20 °C for 1 day, and one of the crystals was picked up and immediately mixed with paratone oil to measure single-crystal XRD.

Crystal data for $(Pd_3\mathbf{L}Cl_6)_2\cdot(\mathbf{SPA})_{1.49}\cdot(H_2O)_{1.5}$: $C_{89.93}H_{97.35}Cl_{12}N_{12}O_{2.98}Pd_6$, F_w = 2457.85, crystal dimensions 0.18 × 0.13 × 0.06 mm^3, monoclinic, space group $P2_1/c$, a = 20.2244(3), b = 53.3374(14), c = 14.3374(2) Å, β = 90.356(1)°, V = 15465.7(5) Å^3, Z = 4, ρ_{calcd} = 1.066 g cm^{-3}, μ = 7.687 mm^{-1}, T = 93 K, λ(CuKα) = 1.54187 Å, $2\theta_{max}$ = 136.5°, 167296/ 30082 reflections collected/unique (R_{int} = 0.0795), R_1 = 0.1483 ($I > 2\sigma(I)$), wR_2 = 0.4496 (for all data), GOF = 1.660, largest diff. peak and hole 5.137/–1.871 eÅ^{-3}. CCDC deposit number 2223923

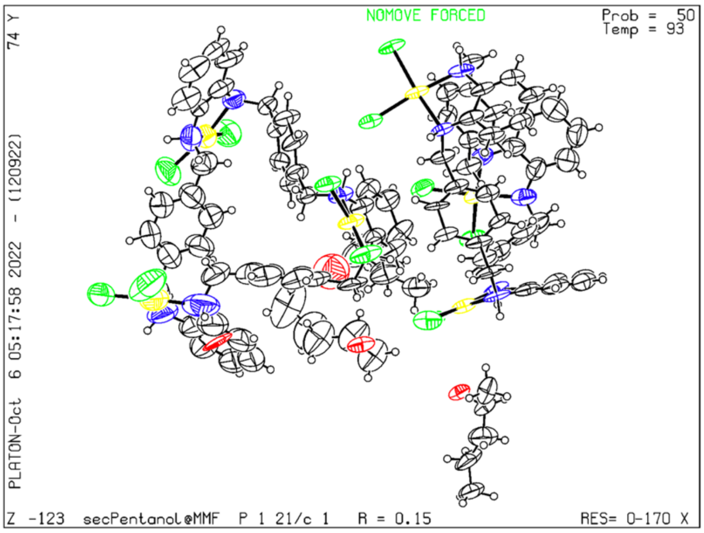

Supplementary Fig. 63 ORTEP drawing of the structurally extended MMF soaked in **SPA** at the 50% probability level. Color: C black, N blue, O red, Cl green and Pd yellow. This figure was produced by the checkCIF report of the International Union of Crystallography.

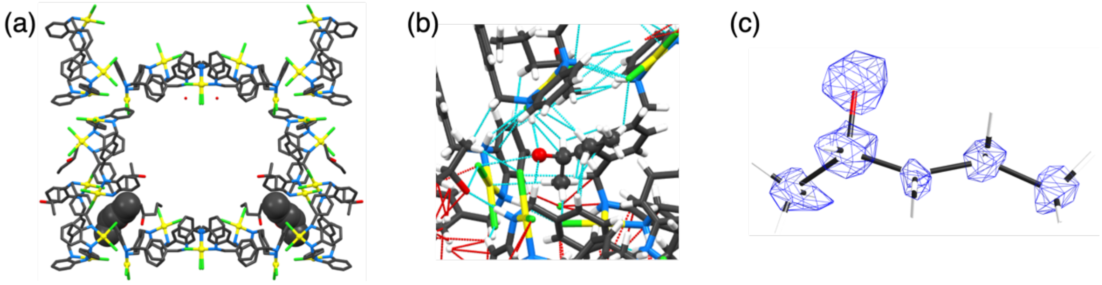

Supplementary Fig. 64 (a) Unit-space structure of MMF accommodating SPA, illustrated by CPK model, as an effector. (b) Short contact around the allosteric site accommodating SPA, illustrated by ball and stick style. (c) The electron density map of SPA (70.5% occupancy) adsorbed in the allosteric binding site (contour level;

1.8 eÅ^{-3}).

•(*rac*)-1-Phenylethanol (PEA)

Soaking procedure: As-crystalised MMF crystals were firstly soaked in dichloromethane in order to remove acetonitrile from the supernatant. Afterwards, the crystals were transferred into (*rac*)-1-phenylethanol (PEA) to be soaked at 20 °C for 8 h, and one of the crystals was picked up and immediately mixed with paratone oil to measure single-crystal XRD.

Crystal data for $(Pd_3\mathbf{L}Cl_6)_2\cdot(\mathbf{PEA})_{1.65}\cdot(H_2O)_2$: $C_{104.65}H_{98.79}Cl_{12}N_{12}O_{3.64}Pd_6$, F_w = 2646.59, crystal dimensions 0.28 × 0.13 × 0.07 mm^3, monoclinic, space group $P2_1/c$, a = 20.6607(3), b = 54.5683(7), c = 14.2293(2) Å, β = 90.972(1)°, V = 16040.1(4) Å^3, Z = 4, ρ_{calcd} = 1.096 g cm^{-3}, μ = 7.450 mm^{-1}, T = 93 K, λ(CuKα) = 1.54187 Å, $2\theta_{max}$ = 136.5°, 175623/ 31297 reflections collected/unique (R_{int} = 0.0785), R_1 = 0.1175 ($I > 2\sigma(I)$), wR_2 = 0.3882 (for all data), GOF = 1.528, largest diff. peak and hole 3.435/–1.891 eÅ^{-3}. CCDC deposit number 2223924

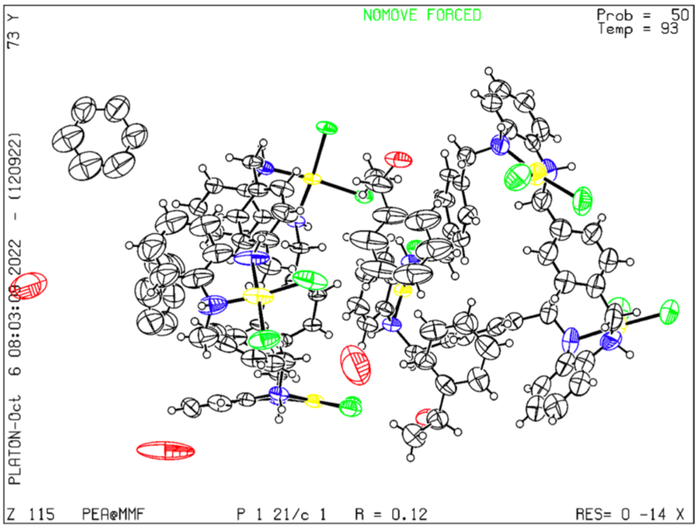

Supplementary Fig. 65 ORTEP drawing of the structurally extended MMF soaked in **PEA** at the 50% probability level. Color: C black, N blue, O red, Cl green, and Pd yellow. This figure was produced by the checkCIF report of the International Union of Crystallography.

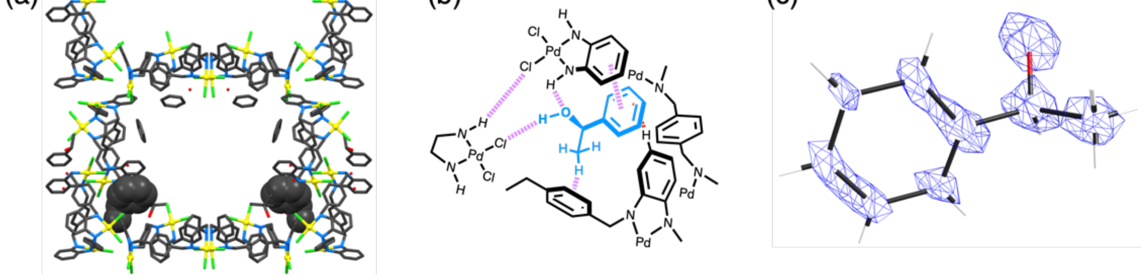

Supplementary Fig. 66 (a) The unit-space structure of the MMF that accommodates PEA shown in the CPK model, as an effector. (b) Interaction patterns around the allosteric site of MMF and PEA. (c) The electron

density map of PEA (85.6% occupancy) adsorbed in the allosteric binding site (contour level; 3.0 eÅ^{-3}).

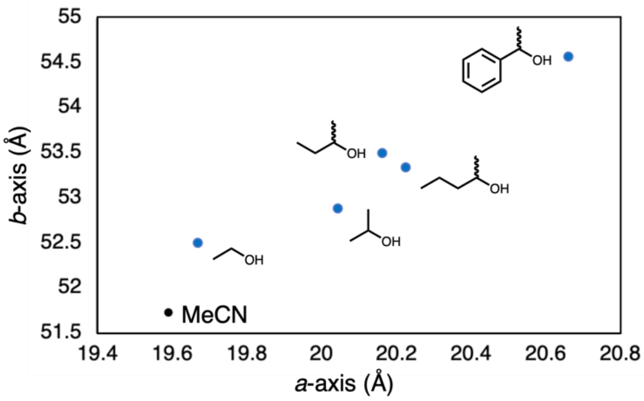

Supplementary Fig. 67 The cell parameter of the extended MMF in five secondary alcohols

•Ethyl acetate (AcOEt)

Soaking procedure: As-crystalised MMF crystals were soaked in ethyl acetate (AcOEt) at 20 °C for 1 day, and one of the crystals was picked up and immediately mixed with paratone oil to measure single-crystal XRD.

Crystal data for $(Pd_3\mathbf{L}Cl_6)_2\cdot(\mathbf{AcOEt})_{0.88}\cdot(H_2O)_3$: $C_{87.53}H_{91.05}Cl_{12}N_{12}O_{4.76}Pd_6$, $F_w = 2451.07$, crystal dimensions $0.30 \times 0.22 \times 0.11$ mm^3, monoclinic, space group $P2_1/c$, $a = 20.0376(4)$, $b = 52.2313(13)$, $c = 14.3310(2)$ Å, $\beta = 91.120(1)°$, $V = 14995.8(5)$ Å^3, $Z = 4$, $\rho_{calcd} = 1.086$ g cm^{-3}, $\mu = 7.936$ mm^{-1}, $T = 93$ K, λ(CuKα) = 1.54187 Å, $2\theta_{max} = 136.5°$, 88371/ 28839 reflections collected/unique ($R_{int} = 0.0447$), $R_1 = 0.1380$ ($I > 2\sigma(I)$), $wR_2 = 0.4353$ (for all data), GOF = 1.640, largest diff. peak and hole 4.189/–2.272 eÅ^{-3}. CCDC deposit number 2223925

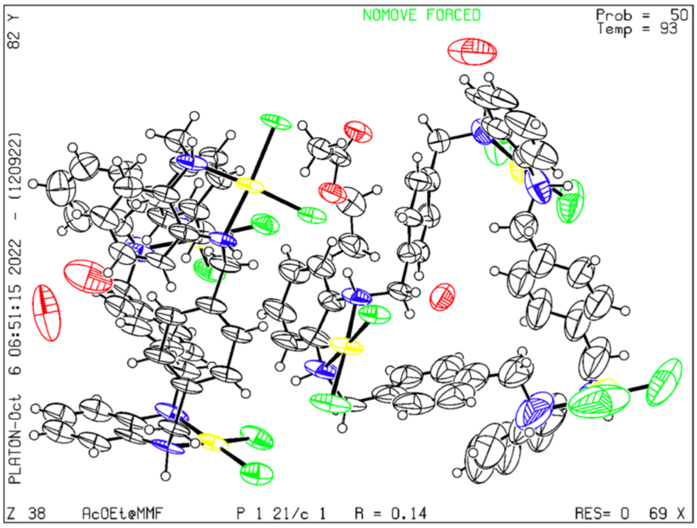

Supplementary Fig. 68 ORTEP drawing of the structurally extended MMF soaked in **AcOEt** at the 50% probability level. Color: C black, N blue, O red, Cl green and Pd yellow. This figure was produced by the checkCIF report of the International Union of Crystallography.

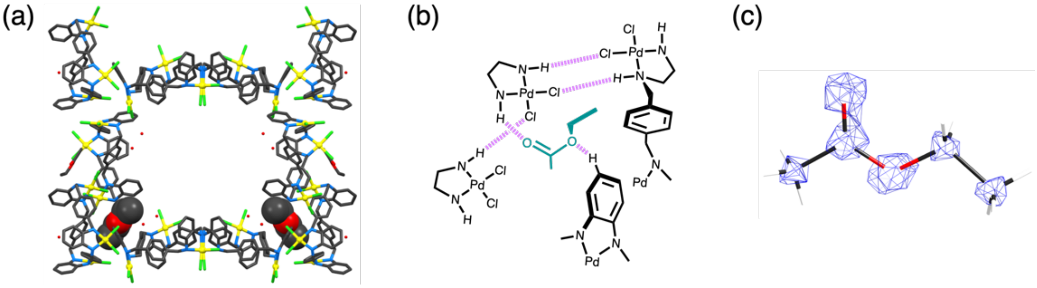

Supplementary Fig. 69 (a) The unit-space structure of the MMF that accommodates AcOE shown in the CPK model, as an effector. (b) Interaction patterns around the allosteric site of MMF and AcOEt. (c) The electron density map of AcOEt (88.1% occupancy) adsorbed in the allosteric binding site (contour level; 2.5 e$Å^{-3}$).

•Ethylene glycol (EG)

Soaking procedure: As-crystalised MMF crystals were firstly soaked in dichloromethane in order to remove acetonitrile from the supernatant. Afterwards, the crystals were transferred into ethylene glycol (EG) to be soaked at 20 °C for 6 days, and one of the crystals was picked up and immediately mixed with paratone oil to measure single-crystal XRD.

Crystal data for $(Pd_3\mathbf{L}Cl_6)_2\cdot(\mathbf{EG})_{3.46}\cdot(H_2O)_{0.5}$: $C_{90.91}H_{97.82}Cl_{12}N_{12}O_{7.41}Pd_6$, F_w = 2540.98, crystal dimensions 0.38 × 0.12 × 0.09 mm^3, monoclinic, space group $P2_1/c$, a = 19.86880(17), b = 53.1417(5), c = 14.29610(9) Å, β = 90.1104(7)°, V = 15093.2(2) $Å^3$, Z = 4, ρ_{calcd} = 1.118 g cm^{-3}, μ = 7.915 mm^{-1}, T = 93 K, λ(CuKα) = 1.54187 Å, $2\theta_{max}$ = 136.5°, 161663/ 27611 reflections collected/unique (R_{int} = 0.0482), R_1 = 0.1122 ($I > 2\sigma(I)$), wR_2 = 0.3296 (for all data), GOF = 1.123, largest diff. peak and hole 3.404/–2.464 e$Å^{-3}$. CCDC deposit number 2223926

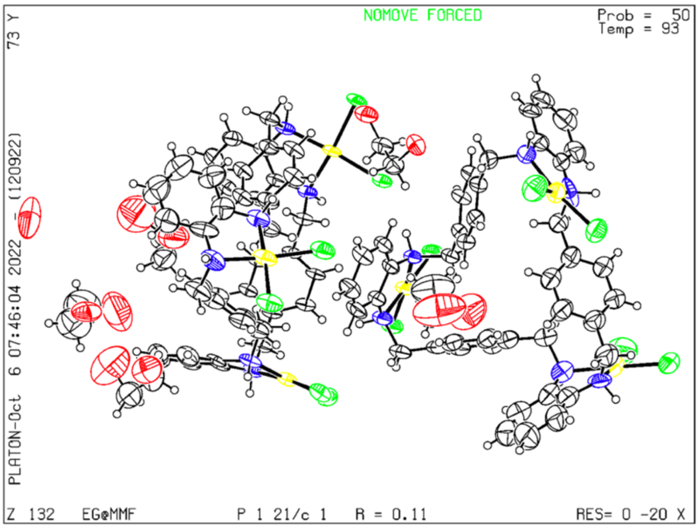

Supplementary Fig. 70 ORTEP drawing of the structurally extended MMF soaked in **EG** at the 50% probability level. Color: C black, N blue, O red, Cl green and Pd yellow. This figure was produced by the checkCIF report of the International Union of Crystallography.

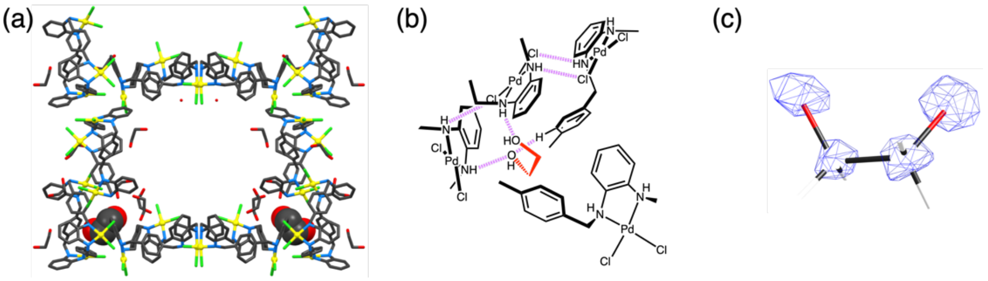

Supplementary Fig. 71 (a) The unit-space structure of the MMF that accommodates EG shown in the CPK model, as an effector. (b) Interaction patterns around the allosteric site of MMF and EG. (c) The electron density map of EG (95.6% occupancy) adsorbed in the allosteric binding site (contour level; 3.0 eÅ^{-3}).

•1,3-Propanediol (PD)

Soaking procedure: As-crystalised MMF crystals were firstly soaked in dichloromethane in order to remove acetonitrile from the supernatant. Afterwards, the crystals were transferred into 1,3-propanediol (PD) to be soaked at 20 °C for 5 h, and one of the crystals was picked up and immediately mixed with paratone oil to measure single-crystal XRD.

Crystal data for $(Pd_3\mathbf{L}Cl_6)_2\cdot(\mathbf{PD})_{1.21}\cdot(H_2O)_{1.5}$: $C_{87.59}H_{91.17}Cl_{12}N_{12}O_{3.89}Pd_6$, F_w = 2437.98, crystal dimensions 0.20 × 0.13 × 0.06 mm^3, monoclinic, space group $P2_1/c$, a = 20.1269(2), b = 52.9679(8), c = 14.4202(1) Å, β = 90.301(1)°, V = 15372.9(3) Å^3, Z = 4, ρ_{calcd} = 1.053 g cm^{-3}, μ = 7.735 mm^{-1}, T = 93 K, λ(CuKα) = 1.54187 Å, $2\theta_{max}$ = 136.5°, 157388/ 29924 reflections collected/unique (R_{int} = 0.0550), R_1 = 0.1255 ($I > 2\sigma(I)$), wR_2 = 0.4117 (for all data), GOF = 1.667, largest diff. peak and hole 3.725/–3.327 eÅ^{-3}. CCDC deposit number 2223927

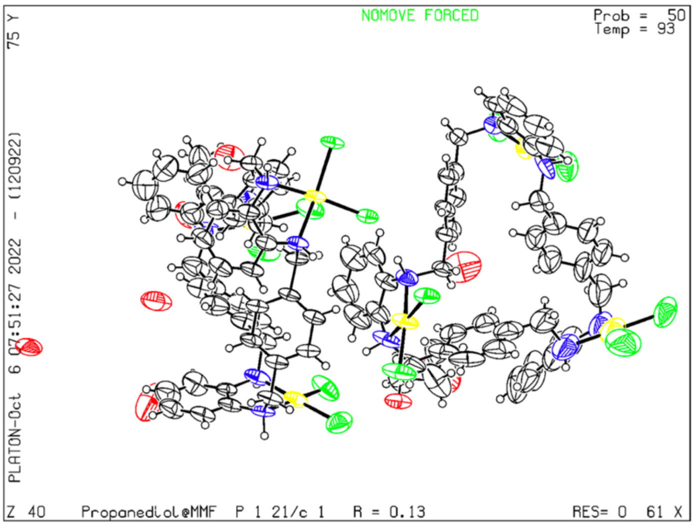

Supplementary Fig. 72 ORTEP drawing of the structurally extended MMF soaked in **PD** at the 50% probability level. Color: C black, N blue, O red, Cl green and Pd yellow. This figure was produced by the checkCIF report of the International Union of Crystallography.

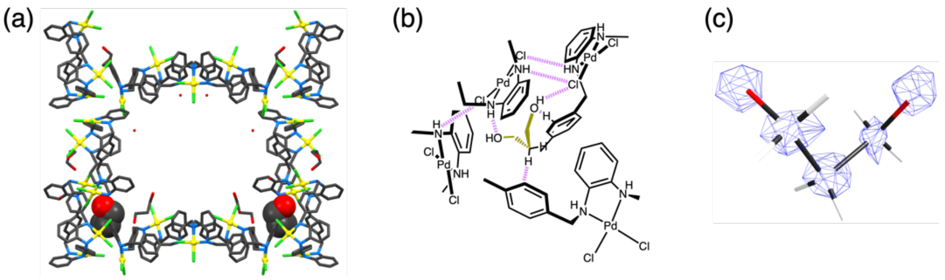

Supplementary Fig. 73 (a) The unit-space structure of the MMF that accommodates PD shown in the CPK model, as an effector. (b) Interaction patterns around the allosteric site of MMF and PD. (c) The electron density map of PD (71.0% occupancy) adsorbed in the allosteric binding site (contour level; 2.0 eÅ^{-3}).

•Diethyl ether (Et_2O)

Soaking procedure: As-crystalised MMF crystals were firstly soaked in DME at 20 °C for 4 h. Afterwards, the crystals were transferred into diethyl ether (Et_2O) to be soaked at 20 °C for 1 day, and one of the crystals was picked up and immediately mixed with paratone oil to measure single-crystal XRD at 93 K. The adsorption of Et_2O was not observed on any parts of the nanochannel.

Crystal data for $(Pd_3\mathbf{L}Cl_6)_2{\cdot}(H_2O)_{1.36}$: $C_{84}H_{84}Cl_{12}N_{12}O_{1.45}Pd_6$, F_w = 2348.63, crystal dimensions 0.11 × 0.05 × 0.04 mm^3, monoclinic, space group $P2_1/c$, a = 18.9524(2), b = 49.6621(11), c = 14.3768(2) Å, β = 92.119(1)°, V = 13522.4(4) Å^3, Z = 4, ρ_{calcd} = 1.154 g cm^{-3}, μ = 8.762 mm^{-1}, T = 93 K, λ(CuKα) = 1.54187 Å, $2\theta_{max}$ = 136.5°, 87890/ 25647 reflections collected/unique (R_{int} = 0.0460), R_1 = 0.1100 ($I > 2\sigma(I)$), wR_2 = 0.3685 (for all data), GOF = 1.336, largest diff. peak and hole 2.012/–1.363 eÅ^{-3}. CCDC deposit number 2223928

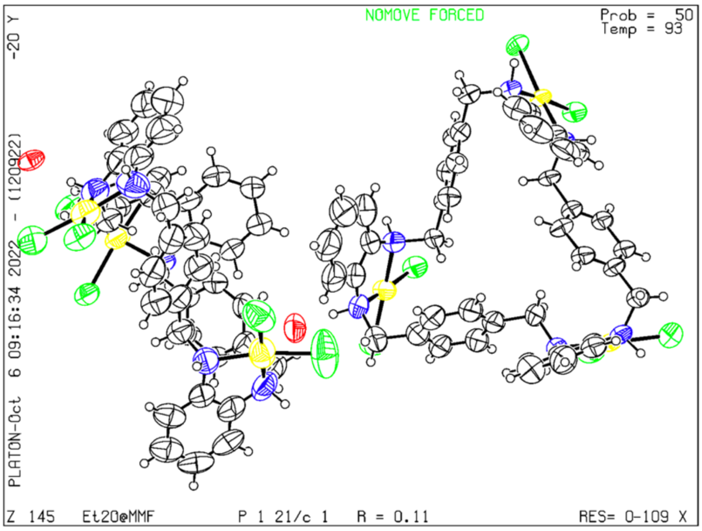

Supplementary Fig. 74 ORTEP drawing of the structurally contracted MMF cooled after soaking in $\mathbf{Et_2O}$ at the 50% probability level. Color: C black, N blue, O red, Cl green and Pd yellow. This figure was produced by the checkCIF report of the International Union of Crystallography.

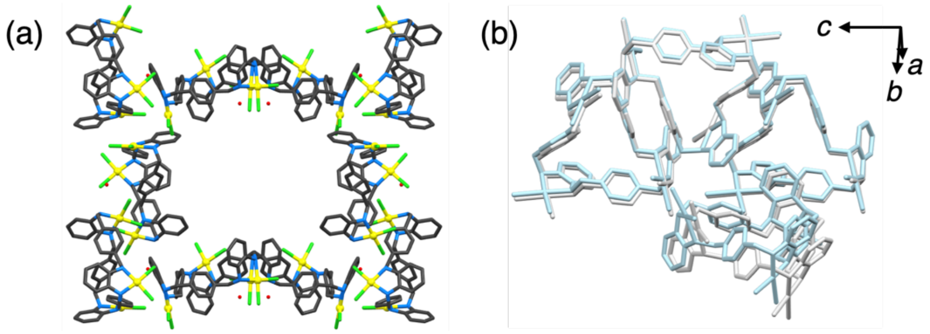

Supplementary Fig. 75 (a) The unit-space structure of the structurally contracted MMF obtained under the cryogenic condition after soaking in Et_2O. (b) Structural overlap of Pd^{II}-macrocycles around the allosteric site before and after the contraction, Grey line indicates as-crystalised MMF in acetonitrile and light blue line indicates structurally-contracted MMF with the allosteric site closed.

•Benzyl alcohol (BnOH)

-First structure (Cluster II)

Soaking procedure: As-crystalised MMF crystals were firstly soaked in dichloromethane in order to remove acetonitrile from the supernatant. Afterwards, the crystals were transferred into benzyl alcohol (BnOH) to be soaked at 20 °C for 3 days, and one of the crystals was picked up and immediately mixed with paratone oil to measure single-crystal XRD.

Crystal data for $(Pd_3\mathbf{L}Cl_6)_2\cdot(\mathbf{BnOH})_{5.39}$: $C_{119.15}H_{103.86}Cl_{12}N_{12}O_{2.84}Pd_6$, $F_w = 2813.06$, crystal dimensions 0.25 × 0.15 × 0.09 mm^3, monoclinic, space group $P2_1/c$, a = 20.4383(2), b = 52.3223(6), c = 14.4312(1) Å, β = 92.561(1)°, V = 15417.0(3) $Å^3$, Z = 4, ρ_{calcd} = 1.212 g cm^{-3}, μ = 7.778 mm^{-1}, T = 93 K, λ(CuKα) = 1.54187 Å, $2\theta_{max}$ = 136.5°, 65598/ 29246 reflections collected/unique (R_{int} = 0.0335), R_1 = 0.0936 ($I > 2\sigma(I)$), wR_2 = 0.3119 (for all data), GOF = 1.213, largest diff. peak and hole 2.655/–3.058 $eÅ^{-3}$. CCDC deposit number 2223929

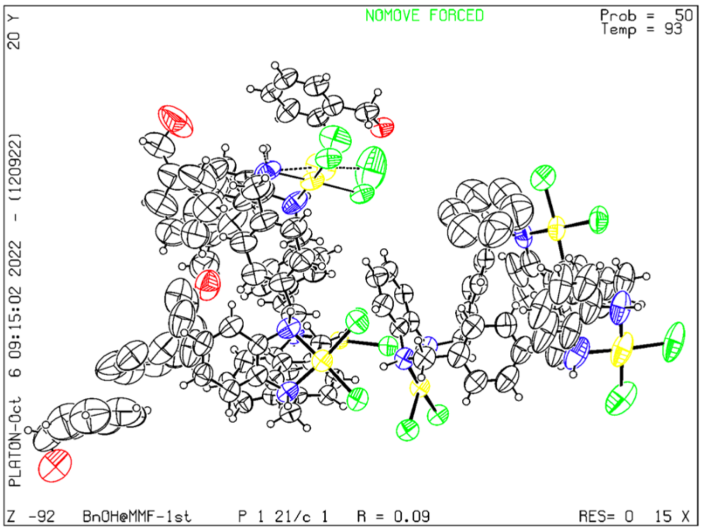

Supplementary Fig. 76 ORTEP drawing of the moderately structurally-extended MMF soaked in **BnOH** at the

50% probability level. Color: C black, N blue, O red, Cl green and Pd yellow. This figure was produced by the checkCIF report of the International Union of Crystallography.

-Second structure (Cluster III)

Soaking procedure: As-crystalised MMF crystals were firstly soaked in DME at 20 °C for 3 days in order to induce the lattice expansion. Afterwards, the crystals were transferred into benzyl alcohol (BnOH) to be soaked at 20 °C for 4 h, and one of the crystals was picked up and immediately mixed with paratone oil to measure single-crystal XRD.

Crystal data for $(Pd_3\mathbf{L}Cl_6)_2\cdot(\mathbf{BnOH})_{2.75}(H_2O)_1$: $C_{102.09}H_{97.18}Cl_{12}N_{12}O_{2.59}Pd_6$, F_w = 2597.34, crystal dimensions 0.24 × 0.10 × 0.07 mm^3, monoclinic, space group $P2_1/c$, a = 22.6779(4), b = 53.3547(12), c = 14.4420(2) Å, β = 97.4152(16)°, V = 17328.3(6) $Å^3$, Z = 4, ρ_{calcd} = 0.996 g cm^{-3}, μ = 6.883 mm^{-1}, T = 93 K, λ(CuKα) = 1.54187 Å, $2\theta_{max}$ = 136.5°, 90801/ 31577 reflections collected/unique (R_{int} = 0.0427), R_1 = 0.1310 ($I > 2\sigma(I)$), wR_2 = 0.4300 (for all data), GOF = 1.718, largest diff. peak and hole 2.257/–2.686 e$Å^{-3}$. CCDC deposit number 2223930

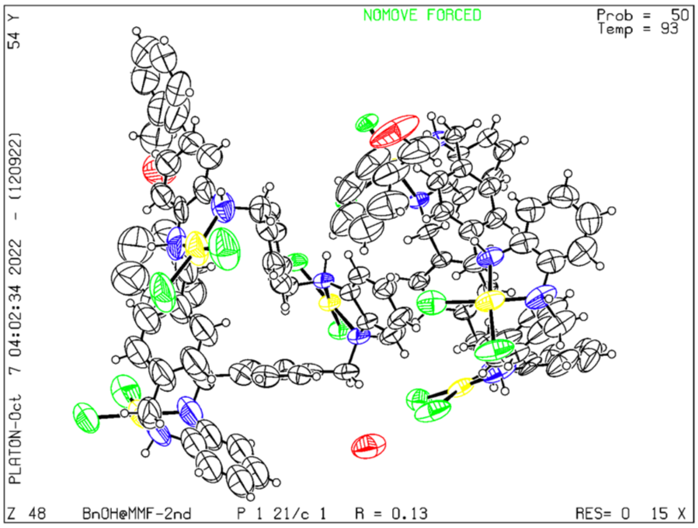

Supplementary Fig. 77 ORTEP drawing of the significantly structurally-extended MMF soaked in **BnOH** at the 50% probability level. Color: C black, N blue, O red, Cl green and Pd yellow. This figure was produced by the checkCIF report of the International Union of Crystallography.

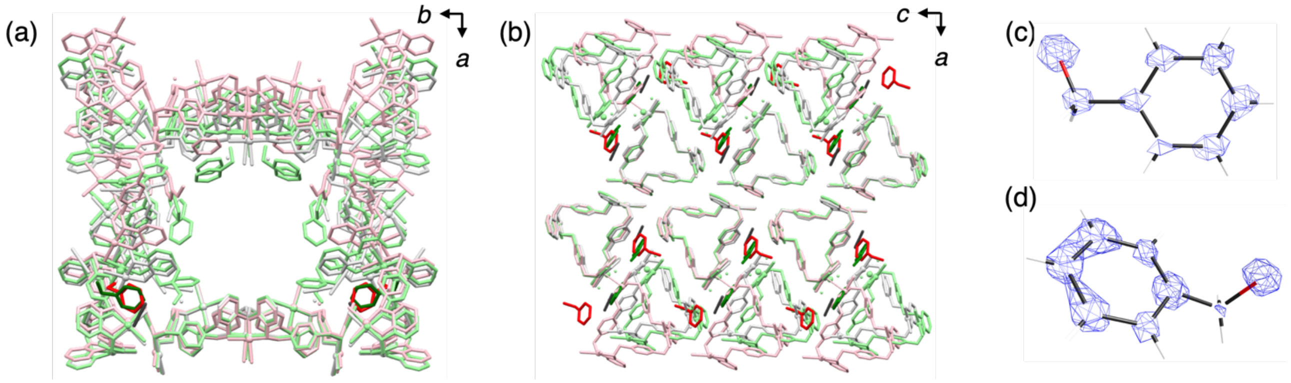

Supplementary Fig. 78 (a), (b) Overlap of three MMF crystal structures: the as-crystalised MMF in acetonitrile (grey), the metastable MMF in BnOH (light green), and the most stable MMF in BnOH (light pink). (a) The unit-space structure and (b) two-dimensional network of *syn*-Pd-macrocycles. (c), (d) The electron density maps of BnOH adsorbed in the allosteric binding site (c) of the metastable MMF (77.8% occupancy, contour level; 2.2 eÅ^{-3}), and (d) of the most stable MMF (100% occupancy, contour level; 1.5 eÅ^{-3}).

•2-Fluorobenzyl alcohol (FBA)

-First structure (Cluster II)

Soaking procedure: As-crystalised MMF crystals were firstly soaked in dichloromethane in order to remove acetonitrile from the supernatant. Afterwards, the crystals were transferred into 2-fluorobenzyl alcohol (FBA) to be soaked at 20 °C for 1 day, and one of the crystals was picked up and immediately mixed with paratone oil to measure single-crystal XRD.

Crystal data for $(Pd_3\mathbf{L}Cl_6)_2\cdot(\mathbf{FBA})_{2.44}(H_2O)_3$: $C_{100.50}H_{95.57}Cl_{12}N_{12}O_{2.84}F_{1.93}Pd_6$, F_w = 2650.68, crystal dimensions 0.22 × 0.15 × 0.08 mm^3, monoclinic, space group $P2_1/c$, a = 20.4933(2), b = 52.4853(9), c = 14.4101(1) Å, β = 92.665(1)°, V = 15482.7(3) Å^3, Z = 4, ρ_{calcd} = 1.137 g cm^{-3}, μ = 7.743 mm^{-1}, T = 93 K, λ(CuKα) = 1.54187 Å, $2\theta_{max}$ = 136.5°, 66256/ 29349 reflections collected/unique (R_{int} = 0.0515), R_1 = 0.1088 ($I > 2\sigma(I)$), wR_2 = 0.3424 (for all data), GOF = 1.229, largest diff. peak and hole 1.628/–2.464 eÅ^{-3}. CCDC deposit number 2223931

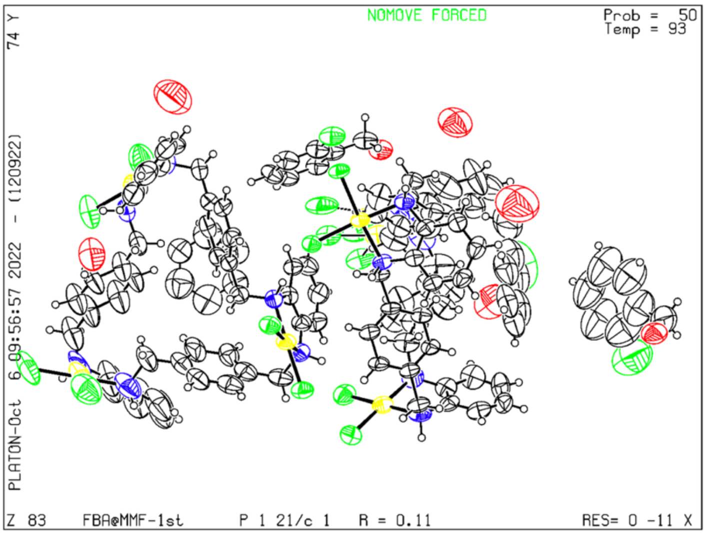

Supplementary Fig. 79 ORTEP drawing of the moderately-extended MMF soaked in **FBA** at the 50% probability level. Color: C black, N blue, O red, Cl green and Pd yellow. This figure was produced by the checkCIF report of the International Union of Crystallography.

-Second structure (Cluster III)

Soaking procedure: As-crystalised MMF crystals were firstly soaked in DME at 20 °C for 3 days in order to induce the lattice expansion. Afterwards, the crystals were transferred into 2-fluorobenzyl alcohol (FBA) to be

soaked at 20 °C for 1 day, and one of the crystals was picked up and immediately mixed with paratone oil to measure single-crystal XRD.

Crystal data for $(Pd_3\mathbf{L}Cl_6)_2\cdot(\mathbf{FBA})_{1.55}(H_2O)_{2.5}$: $C_{94.84}H_{91.79}Cl_{12}F_{1.55}N_{12}O_{3.30}Pd_6$, $F_w = 2545.63$, crystal dimensions $0.24 \times 0.11 \times 0.06$ mm^3, monoclinic, space group $P2_1/c$, $a = 22.7035(5)$, $b = 53.2174(14)$, $c = 14.4098(3)$ Å, $\beta = 97.178(2)°$, $V = 17273.8(7)$ Å^3, $Z = 4$, $\rho_{calcd} = 0.979$ g cm^{-3}, $\mu = 6.913$ mm^{-1}, $T = 93$ K, $\lambda(CuK\alpha) = 1.54187$ Å, $2\theta_{max} = 136.5°$, 70913/ 31185 reflections collected/unique ($R_{int} = 0.0575$), $R_1 = 0.1209$ ($I > 2\sigma(I)$), $wR_2 = 0.3858$ (for all data), GOF = 1.384, largest diff. peak and hole 1.894/–2.303 eÅ^{-3}. CCDC deposit number 2223932

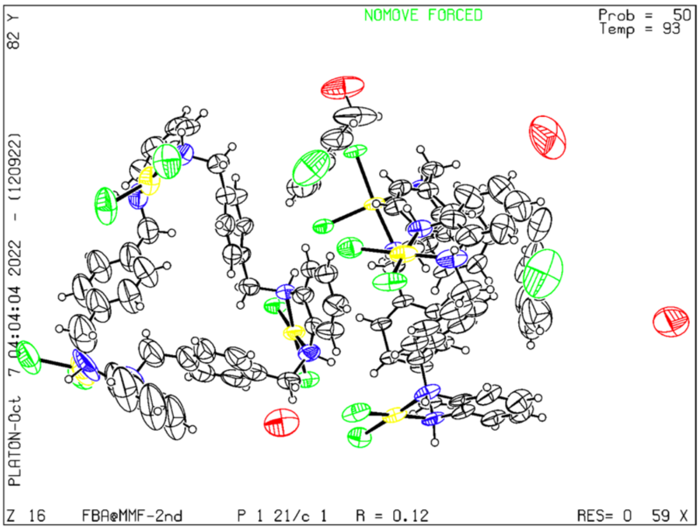

Supplementary Fig. 80 ORTEP drawing of the significantly-extended MMF soaked in **FBA** at the 50% probability level. Color: C black, N blue, O red, Cl green and Pd yellow. This figure was produced by the checkCIF report of the International Union of Crystallography.

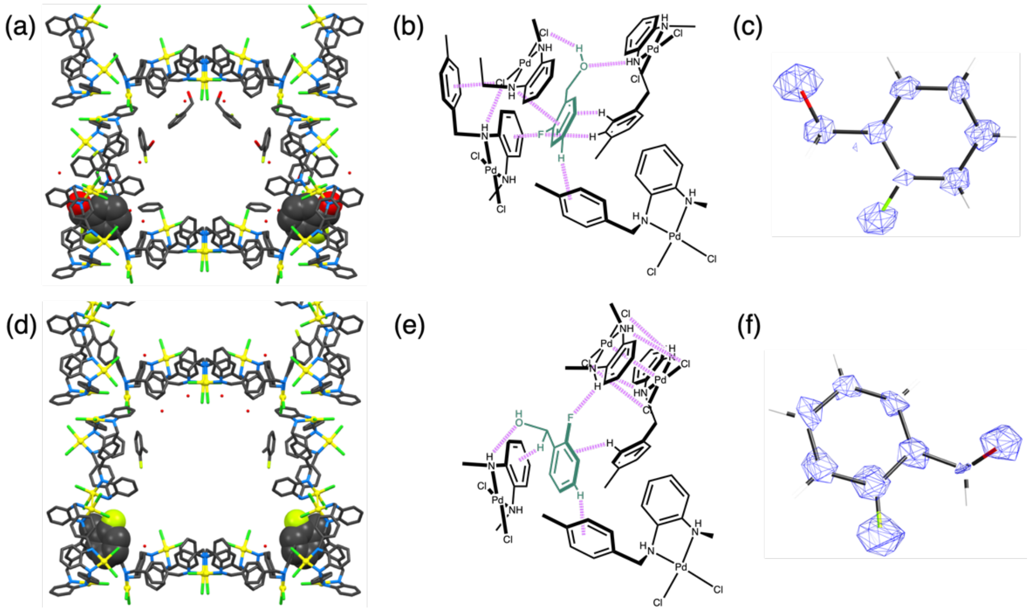

Supplementary Fig. 81 (a) The unit-space structure of the moderately-extended MMF accommodating FBA, illustrated by CPK model, as an effector. (b) Interaction patterns around the allosteric site of the moderately-

extended MMF and FBA. (c) The electron density map of FBA (75.6% occupancy) adsorbed in the allosteric binding site of the moderately-extended MMF. (contour level; 2.2 eÅ^{-3}) (d) Unit-space structure of the significantly-extended MMF accommodating FBA, illustrated by CPK model, as an effector. (e) Interaction patterns around the allosteric site of the significantly-extended MMF and FBA. (f) The electron density map of FBA (79.6% occupancy) adsorbed in the allosteric binding site of the significantly-extended MMF. (contour level; 1.8 eÅ^{-3})

•3-Methoxybenzyl alcohol (MOBA)

-First structure (Cluster II)

Soaking procedure: As-crystalised MMF crystals were firstly soaked in dichloromethane in order to remove acetonitrile from the supernatant. Afterwards, the crystals were transferred into 3-methoxybenzyl alcohol (MOBA) to be soaked at 20 °C for 1 day, and one of the crystals was picked up and immediately mixed with paratone oil to measure single-crystal XRD.

Crystal data for $(Pd_3\mathbf{L}Cl_6)_2\cdot(\mathbf{MOBA})_{2.22}(H_2O)_{1.33}$: $C_{100.20}H_{94.92}Cl_{12}N_{12}O_{4.26}Pd_6$, F_w = 2599.25, crystal dimensions 0.25 × 0.14 × 0.04 mm^3, monoclinic, space group $P2_1/c$, a = 20.6183(2), b = 52.5596(6), c = 14.4117(1) Å, β = 93.271(1)°, V = 15592.4(3) Å^3, Z = 4, ρ_{calcd} = 1.107 g cm^{-3}, μ = 7.658 mm^{-1}, T = 93 K, λ(CuKα) = 1.54187 Å, $2\theta_{max}$ = 136.5°, 171410/ 30415 reflections collected/unique (R_{int} = 0.0805), R_1 = 0.1131 ($I > 2\sigma(I)$), wR_2 = 0.3485 (for all data), GOF = 1.342, largest diff. peak and hole 2.366/–1.996 eÅ^{-3}. CCDC deposit number 2223933

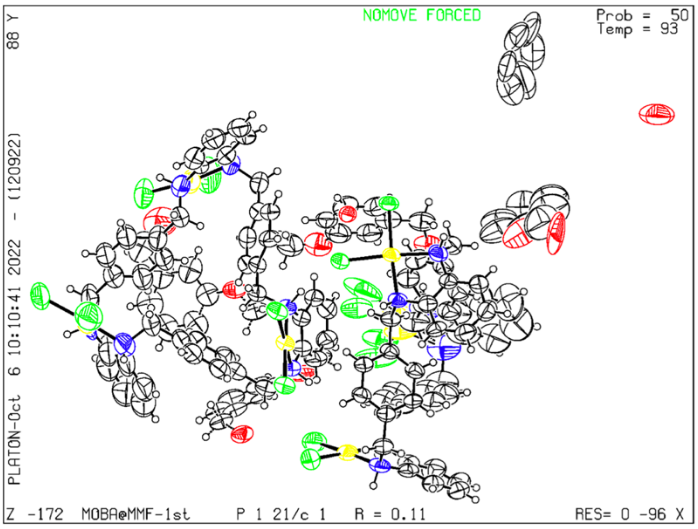

Supplementary Fig. 82 ORTEP drawing of the moderately structurally-extended MMF soaked in **MOBA** at the 50% probability level. Color: C black, N blue, O red, Cl green and Pd yellow. This figure was produced by the checkCIF report of the International Union of Crystallography.

-Second structure (Cluster III)

Soaking procedure: As-crystalised MMF crystals were firstly soaked in DME at 20 °C for 3 days in order to

induce the lattice expansion. Afterwards, the crystals were transferred into 3-methoxybenzyl alcohol (MOBA) to be soaked at 20 °C for 1 day, and one of the crystals was picked up and immediately mixed with paratone oil to measure single-crystal XRD.

Crystal data for $(Pd_3\mathbf{L}Cl_6)_2\cdot(\mathbf{MOBA})_{0.89}(H_2O)_{1.75}$: $C_{91.13}H_{92.03}Cl_{12}N_{12}O_{3.53}Pd_6$, $F_w = 2475.70$, crystal dimensions 0.23 × 0.13 × 0.07 mm^3, monoclinic, space group $P2_1/c$, $a = 22.7282(4)$, $b = 53.3975(13)$, $c = 14.42740(15)$ Å, $\beta = 97.1088(13)$°, $V = 17374.9(6)$ Å^3, $Z = 4$, $\rho_{calcd} = 0.946$ g cm^{-3}, $\mu = 6.849$ mm^{-1}, $T = 93$ K, $\lambda(\mathrm{CuK}\alpha) = 1.54187$ Å, $2\theta_{max} = 136.5$°, 87185/ 31506 reflections collected/unique ($R_{int} = 0.0792$), $R_1 = 0.1387$ ($I > 2\sigma(I)$), $wR_2 = 0.3883$ (for all data), GOF = 1.245, largest diff. peak and hole 2.390/–1.382 eÅ^{-3}. CCDC deposit number 2223934

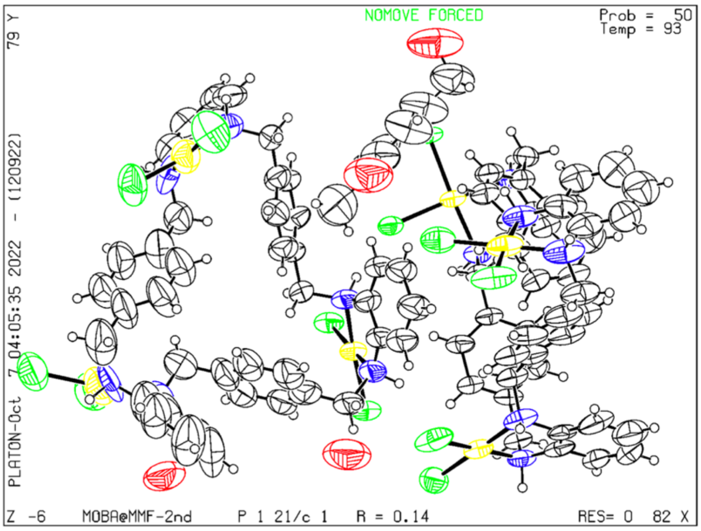

Supplementary Fig. 83 ORTEP drawing of the significantly structurally-extended MMF soaked in **MOBA** at the 50% probability level. Color: C black, N blue, O red, Cl green and Pd yellow. This figure was produced by the checkCIF report of the International Union of Crystallography.

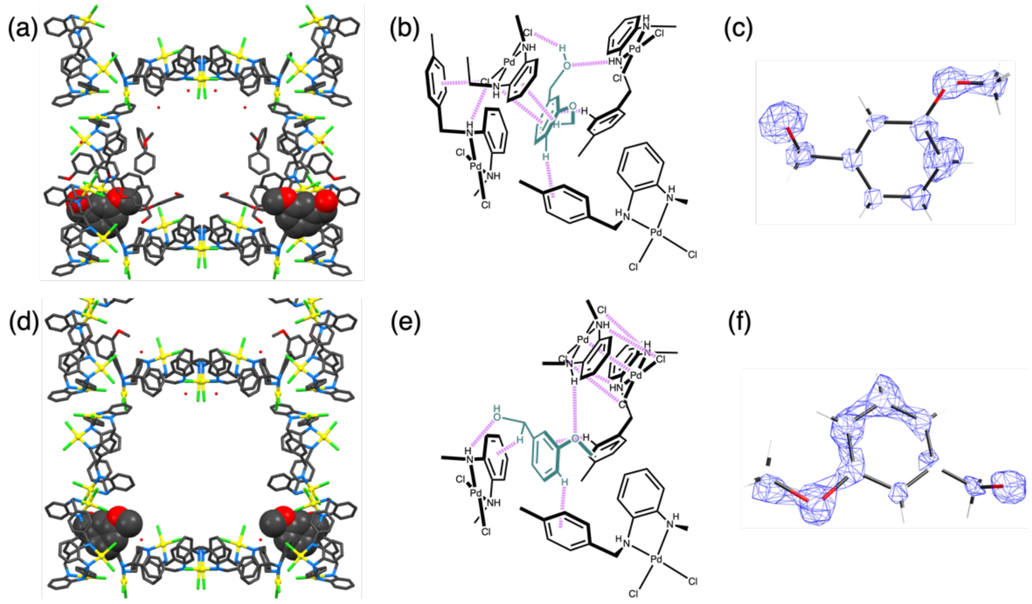

Supplementary Fig. 84 (a) The unit-space structure of the moderately-extended MMF that accommodates MOBA shown in the CPK model, as an effector. (b) Interaction patterns around the allosteric site of the moderately-extended MMF and MOBA. (c) The electron density map of MOBA (72.0% occupancy) adsorbed in the allosteric binding site of the moderately-extended MMF. (contour level; 1.8 e$Å^{-3}$) (d) The unit-space structure of the significantly-extended MMF that accommodates MOBA shown in the CPK model, as an effector. (e) Interaction patterns around the allosteric site of the significantly-extended MMF and MOBA. (f) The electron density map of MOBA (89.4% occupancy) adsorbed in the allosteric binding site of the significantly-extended MMF. (contour level; 1.6 e$Å^{-3}$)

•3-Methylbenzyl alcohol (MBA)

-First structure (Cluster II)

Soaking procedure: As-crystalised MMF crystals were firstly soaked in dichloromethane in order to remove acetonitrile from the supernatant. Afterwards, the crystals were transferred into 3-methylbenzyl alcohol (MBA) to be soaked at 20 °C for 1 day, and one of the crystals was picked up and immediately mixed with paratone oil to measure single-crystal XRD.

Crystal data for $(Pd_3\mathbf{L}Cl_6)_2\cdot(\mathbf{MBA})_{2.38}(H_2O)_{1.}$: $C_{103.03}H_{100.91}Cl_{12}N_{12}O_{2.88}Pd_6$, $F_w = 2617.13$, crystal dimensions $0.24 \times 0.09 \times 0.07$ mm^3, monoclinic, space group $P2_1/c$, $a = 20.5563(2)$, $b = 52.7166(7)$, $c = 14.4801(1)$ Å, $\beta = 93.308(1)°$, $V = 15665.3(3)$ Å^3, $Z = 4$, $\rho_{calcd} = 1.110$ g cm^{-3}, $\mu = 7.618$ mm^{-1}, $T = 93$ K, λ(CuKα) = 1.54187 Å, $2\theta_{max} = 136.5°$, 71145/ 29785 reflections collected/unique ($R_{int} = 0.0738$), $R_1 = 0.1217$ ($I > 2\sigma(I)$), $wR_2 = 0.3741$ (for all data), GOF = 1.413, largest diff. peak and hole 3.087/–2.676 e$Å^{-3}$. CCDC deposit number 2223935

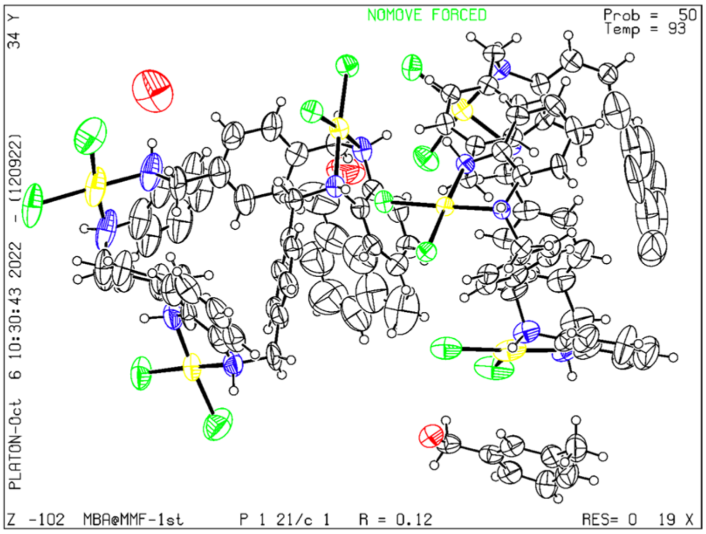

Supplementary Fig. 85 ORTEP drawing of the moderately structurally-extended MMF soaked in **MBA** at the 50% probability level. Color: C black, N blue, O red, Cl green and Pd yellow. This figure was produced by the checkCIF report of the International Union of Crystallography.

-Second structure (Cluster III)

Soaking procedure: As-crystalised MMF crystals were firstly soaked in DME at 20 °C for 1day in order to induce the lattice expansion. Afterwards, the crystals were transferred into 3-methylbenzyl alcohol (MBA) to be soaked at 20 °C for 1 day, and one of the crystals was picked up and immediately mixed with paratone oil to measure single-crystal XRD.

Crystal data for $(Pd_3\mathbf{L}Cl_6)_2\cdot(\mathbf{MBA})_{4.45}(H_2O)_1$: $C_{119.21}H_{119.68}Cl_{12}N_{12}O_{4.96}Pd_6$, F_w = 2863.76, crystal dimensions 0.22 × 0.14 × 0.08 mm^3, monoclinic, space group $P2_1/c$, a = 22.6690(4), b = 53.5740(8), c = 14.39750(19) Å, β = 97.0403(14)°, V = 17353.5(5) Å^3, Z = 4, ρ_{calcd} = 1.096 g cm^{-3}, μ = 6.926 mm^{-1}, T = 93 K, λ(CuKα) = 1.54187 Å, $2\theta_{max}$ = 136.5°, 69881/ 31300 reflections collected/unique (R_{int} = 0.0538), R_1 = 0.1026 ($I > 2\sigma(I)$), wR_2 = 0.3090 (for all data), GOF = 1.070, largest diff. peak and hole 3.302/–2.081 eÅ^{-3}. CCDC deposit number 2223936

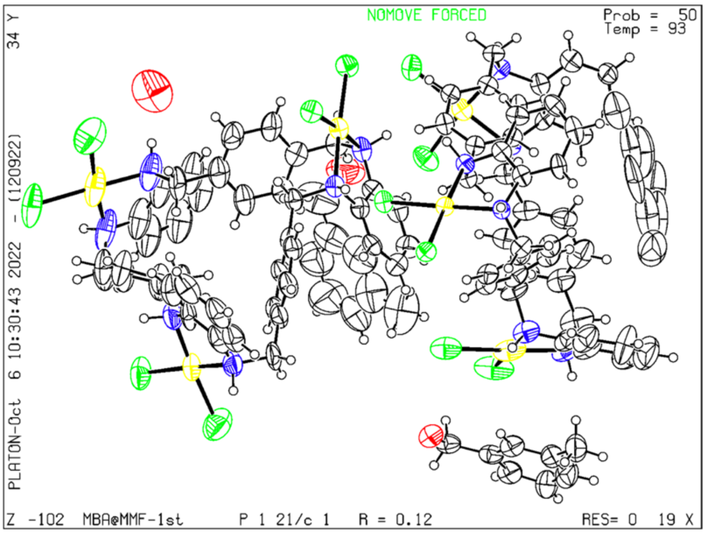

Supplementary Fig. 86 ORTEP drawing of the significantly structurally-extended MMF soaked in **MBA** at the 50% probability level. Color: C black, N blue, O red, Cl green and Pd yellow. This figure was produced by the checkCIF report of the International Union of Crystallography.

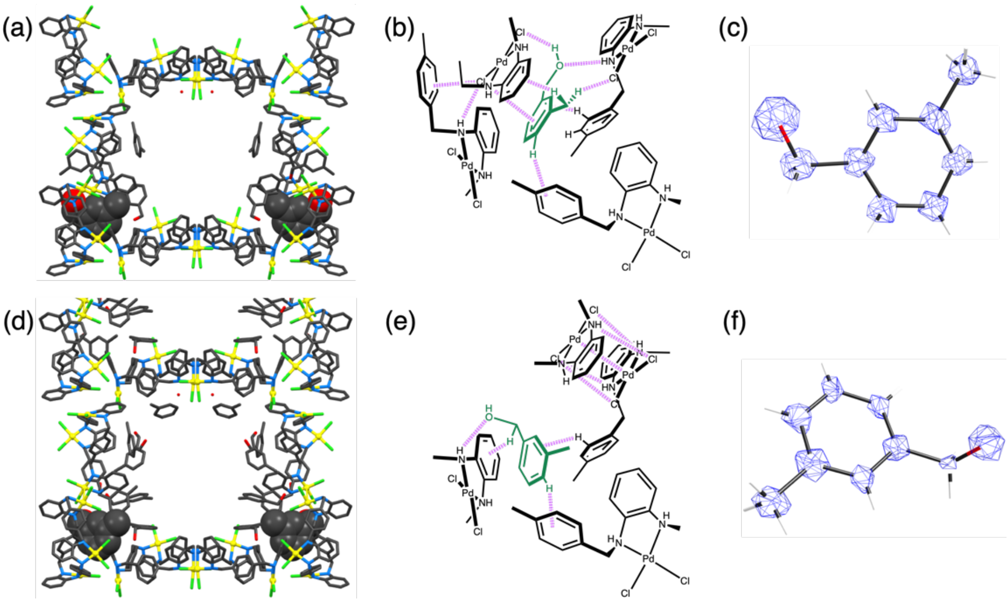

Supplementary Fig. 87 (a) The unit-space structure of the moderately-extended MMF that accommodates MBA shown in the CPK model, as an effector. (b) Interaction patterns around the allosteric site of the moderately-extended MMF and MBA. (c) The electron density map of MBA (93.1% occupancy) adsorbed in the allosteric binding site of the moderately-extended MMF (contour level; 2.5 eÅ^{-3}). (d) The unit-space structure of the significantly-extended MMF that accommodates MBA shown in the CPK model, as an effector. (e) Interaction patterns around the allosteric site of the significantly-extended MMF and MBA. (f) The electron density map of MBA (82.6% occupancy) adsorbed in the allosteric binding site of the significantly-extended MMF. (contour level; 2.5 eÅ^{-3})

•3-Bromobenzyl alcohol (BBA)

-First structure (Cluster II)

Soaking procedure: As-crystalised MMF crystals were firstly soaked in dichloromethane in order to remove acetonitrile from the supernatant. Afterwards, the crystals were transferred into 3-bromobenzyl alcohol (BBA) to be soaked at 20 °C for 1 day, and one of the crystals was picked up and immediately mixed with paratone oil to measure single-crystal XRD.

Crystal data for $(Pd_3\mathbf{L}Cl_6)_2\cdot(\mathbf{BBA})_{2.30}(H_2O)_{4.96}$: $C_{93.26}H_{91.94}Br_{2.30}Cl_{12}N_{12}O_{6.28}Pd_6$, F_w = 2729.08, crystal dimensions 0.19 × 0.12 × 0.07 mm^3, monoclinic, space group $P2_1/c$, a = 20.6267(3), b = 52.5972(11), c = 14.4553(2) Å, β = 93.519(1)°, V = 15653.1(5) Å^3, Z = 4, ρ_{calcd} = 1.158 g cm^{-3}, μ = 8.322 mm^{-1}, T = 93 K, λ(CuKα) = 1.54187 Å, $2\theta_{max}$ = 136.5°, 69445/ 29628 reflections collected/unique (R_{int} = 0.0691), R_1 = 0.1163 ($I > 2\sigma(I)$), wR_2 = 0.3611 (for all data), GOF = 1.249, largest diff. peak and hole 2.490/–2.735 eÅ^{-3}. CCDC deposit number 2223937

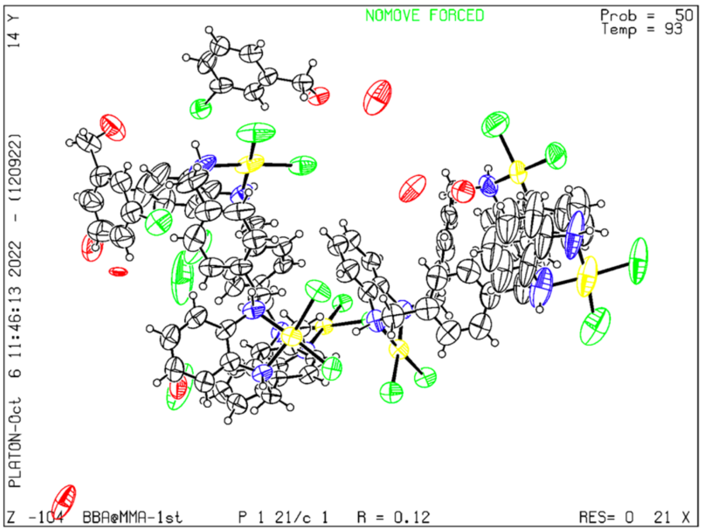

Supplementary Fig. 88 ORTEP drawing of the moderately structurally-extended MMF soaked in **BBA** at the 50% probability level. Color: C black, N blue, O red, Cl/Br green and Pd yellow. This figure was produced by the checkCIF report of the International Union of Crystallography.

-Second structure (Cluster III)

Soaking procedure: As-crystalised MMF crystals were firstly soaked in DME at 20 °C for 1day in order to induce the lattice expansion. Afterwards, the crystals were transferred into 3-bromobenzyl alcohol (BBA) to be soaked at 20 °C for 1 day, and one of the crystals was picked up and immediately mixed with paratone oil to measure single-crystal XRD.

Crystal data for $(Pd_3\mathbf{L}Cl_6)_2\cdot(\mathbf{BBA})_{2.88}(H_2O)_{1.4}$: $C_{89.77}H_{88.94}Br_{2.89}Cl_{12}N_{12}O_{2.22}Pd_6$, F_w = 2666.05, crystal dimensions 0.14 × 0.06 × 0.05 mm^3, monoclinic, space group $P2_1/c$, a = 22.6621(4), b = 53.3977(17), c = 14.4423(2) Å, β = 97.2344(16)°, V = 17337.6(7) Å^3, Z = 4, ρ_{calcd} = 1.021 g cm^{-3}, μ = 7.637 mm^{-1}, T = 93 K, λ(CuKα) = 1.54187 Å, $2\theta_{max}$ = 136.5°, 76931/ 31283 reflections collected/unique (R_{int} = 0.1075), R_1 = 0.1628 ($I > 2\sigma(I)$), wR_2 = 0.4276 (for all data), GOF = 1.362, largest diff. peak and hole 2.887/–1.613 eÅ^{-3}. CCDC deposit number 2223938

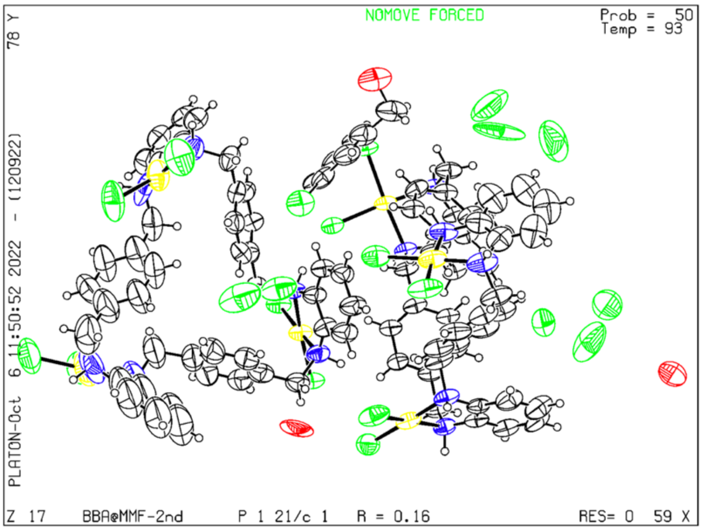

Supplementary Fig. 89 ORTEP drawing of the significantly structurally-extended MMF soaked in **BBA** at the 50% probability level. Color: C black, N blue, O red, Cl/Br green and Pd yellow. This figure was produced by the checkCIF report of the International Union of Crystallography.

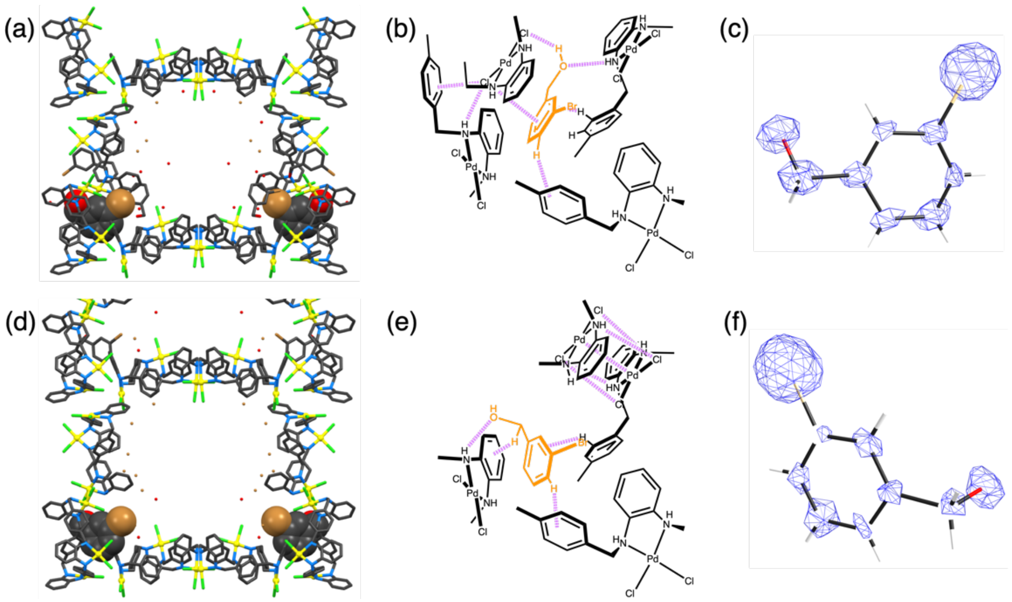

Supplementary Fig. 90 (a) The unit-space structure of the moderately-extended MMF that accommodates BBA shown in the CPK model, as an effector. (b) Interaction patterns around the allosteric site of the moderately-extended MMF and BBA. (c) The electron density map of BBA (77.9% occupancy) adsorbed in the allosteric binding site of the moderately-extended MMF (contour level; 2.7 eÅ^{-3}). (d) The unit-space structure of the significantly-extended MMF that accommodates BBA shown in the CPK model, as an effector. (e) Interaction patterns around the allosteric site of the significantly-extended MMF and BBA. (f) The electron density map of BBA (82.4% occupancy) adsorbed in the allosteric binding site of the significantly-extended MMF. (contour level; 2.7 eÅ^{-3})

•Phenol (PhOH)

-First structure (Cluster II)

Soaking procedure: As-crystalised MMF crystals were firstly soaked in dichloromethane in order to remove acetonitrile from the supernatant. Afterwards, the crystals were transferred into 1,2,3,4-tetrahydronaphthalene solution of phenol (1.0 M) to be soaked at 20 °C for 1 h, and one of the crystals was picked up and immediately mixed with paratone oil to measure single-crystal XRD.

Crystal data for $(Pd_3\mathbf{L}Cl_6)_2\cdot(\mathbf{PhOH})_{2.35}(H_2O)_{1.5}$: $C_{98.11}H_{95.76}Cl_{12}N_{12}O_{3.85}Pd_6$, F_w = 2568.41, crystal dimensions 0.18 × 0.10 × 0.07 mm^3, monoclinic, space group $P2_1/c$, a = 20.4692(3), b = 52.094(2), c = 14.5400(4) Å, β = 93.177(3)°, V = 15480.5(9) Å^3, Z = 4, ρ_{calcd} = 1.102 g cm^{-3}, μ = 7.705 mm^{-1}, T = 93 K, λ(CuKα) = 1.54187 Å, $2\theta_{max}$ = 136.5°, 77341/ 29385 reflections collected/unique (R_{int} = 0.0587), R_1 = 0.1549 ($I > 2\sigma(I)$), wR_2 = 0.4498 (for all data), GOF = 1.528, largest diff. peak and hole 2.314/–2.092 eÅ^{-3}. CCDC deposit number 2223939

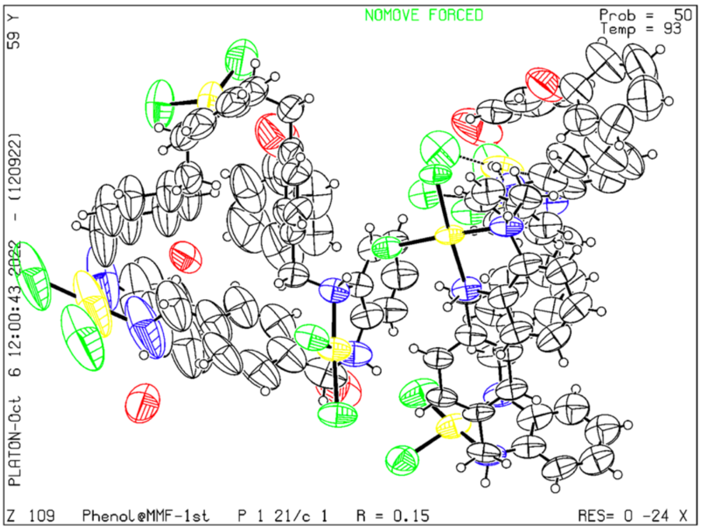

Supplementary Fig. 91 ORTEP drawing of the moderately structurally-extended MMF soaked in 1,2,3,4-tetrahydronaphthalene solution of **PhOH** at the 50% probability level. Color: C black, N blue, O red, Cl green and Pd yellow. This figure was produced by the checkCIF report of the International Union of Crystallography.

-Second structure (Cluster III)

Soaking procedure: As-crystalised MMF crystals were firstly soaked in DME at 20 °C for 1day in order to induce the lattice expansion. Afterwards, the crystals were transferred into 1,2,3,4-tetrahydronaphthalene solution of phenol (2.0 M) to be soaked at 20 °C for 1 day, and one of the crystals was picked up and immediately mixed with paratone oil to measure single-crystal XRD.

Crystal data for $(Pd_3\mathbf{L}Cl_6)_2\cdot(\mathbf{PhOH})_{2.9}$: $C_{101.19}H_{98.32}Cl_{12}N_{12}O_{2.86}Pd_6$, F_w = 2591.99, crystal dimensions 0.18× 0.07 × 0.06 mm^3, monoclinic, space group $P2_1/c$, a = 22.4849(4), b = 51.1640(17), c = 14.58770(18) Å, β = 97.2493(13)°, V = 16647.8(7) Å^3, Z = 4, ρ_{calcd} = 1.034 g cm^{-3}, μ = 7.165 mm^{-1}, T = 93 K, λ(CuKα) = 1.54187 Å, $2\theta_{max}$ = 136.5°, 70034/ 30159 reflections collected/unique (R_{int} = 0.0476), R_1 = 0.1399 ($I > 2\sigma(I)$), wR_2 =

0.4284 (for all data), GOF = 1.513, largest diff. peak and hole 3.415/–2.597 eÅ^{-3}. CCDC deposit number 2223940

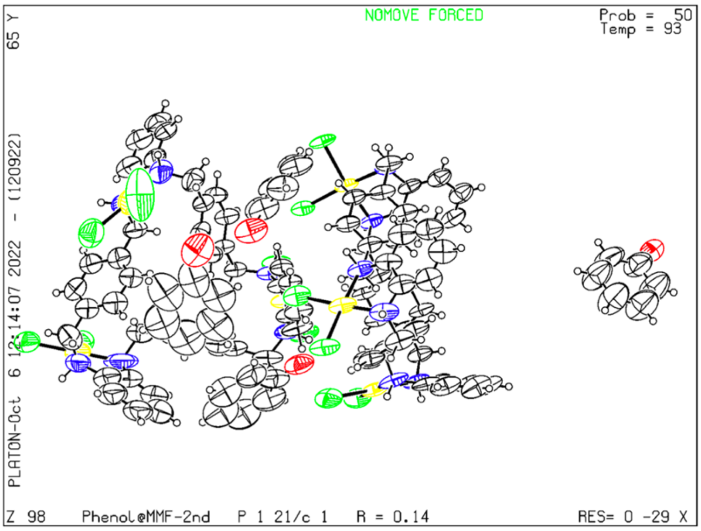

Supplementary Fig. 92 ORTEP drawing of the significantly structurally-extended MMF soaked in 1,2,3,4-tetrahydronaphthalene solution of **PhOH** at the 50% probability level. Color: C black, N blue, O red, Cl green and Pd yellow. This figure was produced by the checkCIF report of the International Union of Crystallography.

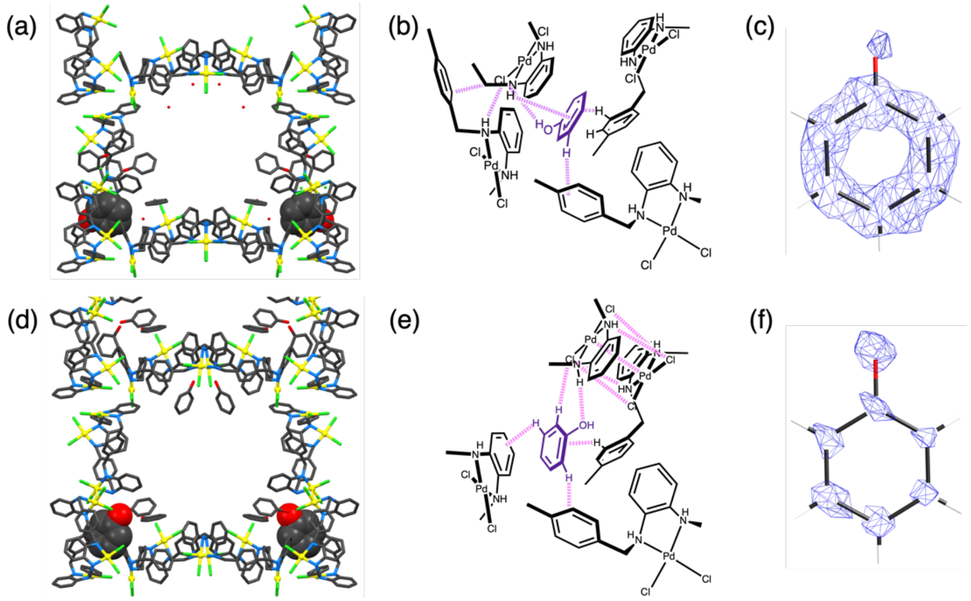

Supplementary Fig. 93 (a) The unit-space structure of the moderately-extended MMF that accommodates PhOH shown in the CPK model, as an effector. (b) Interaction patterns around the allosteric site of the moderately-extended MMF and PhOH. (c) The electron density map of PhOH (78.5% occupancy) adsorbed in the allosteric binding site of the moderately-extended MMF (contour level; 1.2 eÅ^{-3}). (d) The unit-space structure of the significantly-extended MMF that accommodates PhOH shown in the CPK model, as an effector. (e) Interaction patterns around the allosteric site of the significantly-extended MMF and PhOH. (f) The electron density map of PhOH (84.0% occupancy) adsorbed in the allosteric binding site of the significantly-extended MMF (contour level; 2.0 eÅ^{-3}).

•*p*-Cresol

-First structure (Cluster II)

Soaking procedure: As-crystalised MMF crystals were firstly soaked in dichloromethane in order to remove acetonitrile from the supernatant. Afterwards, the crystals were transferred into 1,2,3,4-tetrahydronaphthalene solution of *p*-cresol (2.0 M) to be soaked at 20 °C for 3 h, and one of the crystals was picked up and immediately mixed with paratone oil to measure single-crystal XRD.

Crystal data for $(Pd_3\mathbf{L}Cl_6)_2\cdot$(***p*-cresol**)$_{2.32}\cdot$(tetralin)$_{0.5}\cdot(H_2O)_{0.5}$: $C_{105.24}H_{106.24}Cl_{12}N_{12}O_{2.82}Pd_6$, F_w = 2648.12, crystal dimensions 0.23 × 0.06 × 0.05 mm^3, monoclinic, space group $P2_1/c$, a = 20.5358(4), b = 51.8755(15), c = 14.5073(2) Å, β = 93.209(2)°, V = 15430.5(6) Å^3, Z = 4, ρ_{calcd} = 1.140 g cm^{-3}, μ = 7.739 mm^{-1}, T = 93 K, λ(CuKα) = 1.54187 Å, $2\theta_{max}$ = 136.5°, 65500/ 29165 reflections collected/unique (R_{int} = 0.0497), R_1 = 0.1188 ($I > 2\sigma(I)$), wR_2 = 0.3782 (for all data), GOF = 1.234, largest diff. peak and hole 1.996/–1.888 eÅ^{-3}. CCDC deposit number 2223941

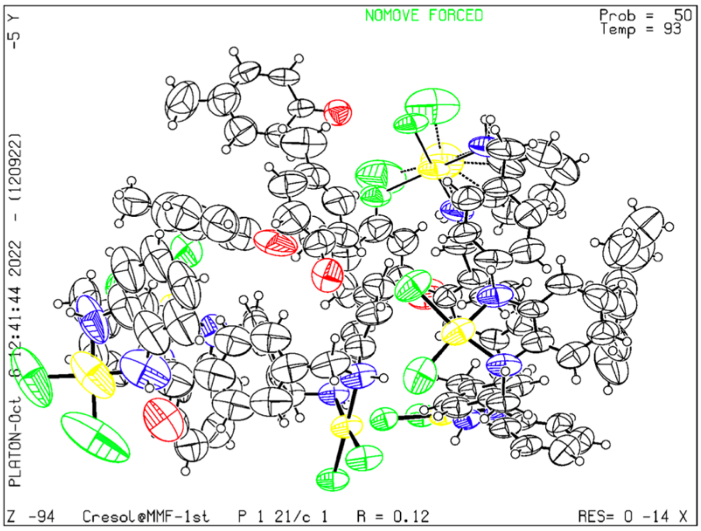

Supplementary Fig. 94 ORTEP drawing of the moderately structurally-extended MMF soaked in 1,2,3,4-tetrahydronaphthalene solution of ***p*-cresol** at the 50% probability level. Color: C black, N blue, O red, Cl green and Pd yellow. This figure was produced by the checkCIF report of the International Union of Crystallography.

-Second structure (Cluster III)

Soaking procedure: As-crystalised MMF crystals were firstly soaked in DME at 20 °C for 1 day in order to induce the lattice expansion. Afterwards, the crystals were transferred into 1,2,3,4-tetrahydronaphthalene solution of *p*-cresol (2.0 M) to be soaked for at 20 °C,1 day and one of the crystals was picked up and immediately mixed with paratone oil to measure single-crystal XRD.

Crystal data for $(Pd_3\mathbf{L}Cl_6)_2\cdot$(***p*-cresol**)$_{0.5}\cdot(H_2O)_1$: $C_{87.50}H_{87.50}Cl_{12}N_{12}O_{1.50}Pd_6$, F_w = 2394.99, crystal dimensions

0.19× 0.08 × 0.05 mm^3, monoclinic, space group $P2_1/c$, a = 22.4169(7), b = 52.813(3), c = 14.4306(3) Å, β = 96.892(2)°, V = 16961.0(12) Å^3, Z = 4, ρ_{calcd} = 0.938 g cm^{-3}, μ = 6.993 mm^{-1}, T = 93 K, λ(CuKα) = 1.54187 Å, $2\theta_{max}$ = 136.5°, 70704/ 30277 reflections collected/unique (R_{int} = 0.0533), R_1 = 0.1472 ($I > 2\sigma(I)$), wR_2 = 0.4516 (for all data), GOF = 1.567, largest diff. peak and hole 1.824/–1.676 eÅ^{-3}. CCDC deposit number 2223942

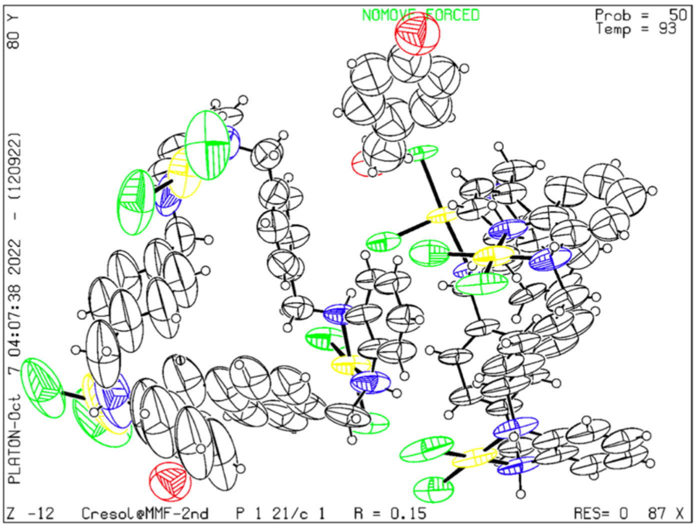

Supplementary Fig. 95 ORTEP drawing of the significantly structurally-extended MMF soaked in 1,2,3,4-tetrahydronaphthalene solution of ***p*-cresol** at the 50% probability level. Color: C black, N blue, O red, Cl green and Pd yellow. This figure was produced by the checkCIF report of the International Union of Crystallography.

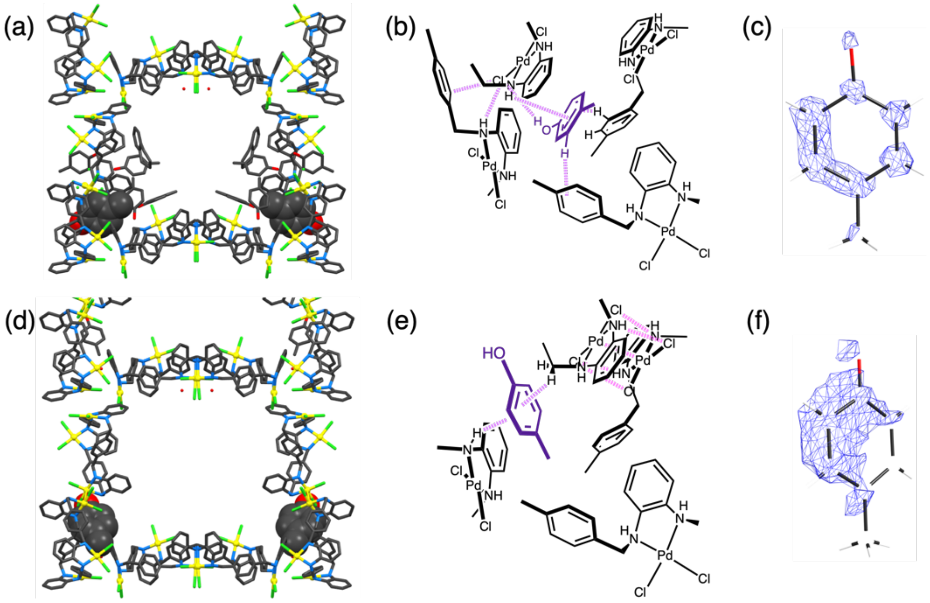

Supplementary Fig. 96 (a) The unit-space structure of the moderately-extended MMF that accommodates *p*-cresol shown in the CPK model, as an effector. (b) Interaction patterns around the allosteric site of the moderately-extended MMF and *p*-cresol. (c) The electron density map of *p*-cresol (82.2% occupancy) adsorbed in the allosteric binding site of the moderately-extended MMF. (contour level; 1.8 eÅ^{-3}) (d) The unit-space structure of the significantly-extended MMF that accommodates *p*-cresol shown in the CPK model, as an effector. (e) Interaction patterns around the allosteric site of the significantly-extended MMF and *p*-cresol. (f)

The electron density map of *p*-cresol (50.0% occupancy) adsorbed in the allosteric binding site of the significantly-extended MMF (contour level; 1.0 eÅ^{-3}).

•<u>Investigation of transformation speed</u>

-DME (*soaking for 50 seconds*)

Soaking procedure: As-crystalised MMF crystals were firstly soaked in dichloromethane in order to remove acetonitrile from the supernatant. Afterwards, the crystals were transferred into DME at 20 °C, and one of the crystals was picked up and immediately mixed with paratone oil to measure single-crystal XRD. The total soaking time was 50 sec before the MMF was set on the cryogenic condition.

Crystal data for $(Pd_3\mathbf{L}Cl_6)_2\cdot(\mathbf{DME})_2\cdot(H_2O)_{2.44}$: $C_{92}H_{104}Cl_{12}N_{12}O_{6.44}Pd_6$, F_w = 2544.75, crystal dimensions 0.15 × 0.09 × 0.05 mm^3, monoclinic, space group $P2_1/c$, a = 22.0470(6), b = 51.293(2), c = 14.5432(3) Å, β = 97.323(2)°, V = 16312.1(8) Å^3, Z = 4, ρ_{calcd} = 1.036 g cm^{-3}, μ = 7.319 mm^{-1}, T = 93 K, λ(CuKα) = 1.54187 Å, $2\theta_{max}$ = 136.5°, 87760/ 29651 reflections collected/unique (R_{int} = 0.0887), R_1 = 0.1670 ($I > 2\sigma(I)$), wR_2 = 0.4828 (for all data), GOF = 1.488, largest diff. peak and hole 2.555/–2.710 eÅ^{-3}. CCDC deposit number 2223947

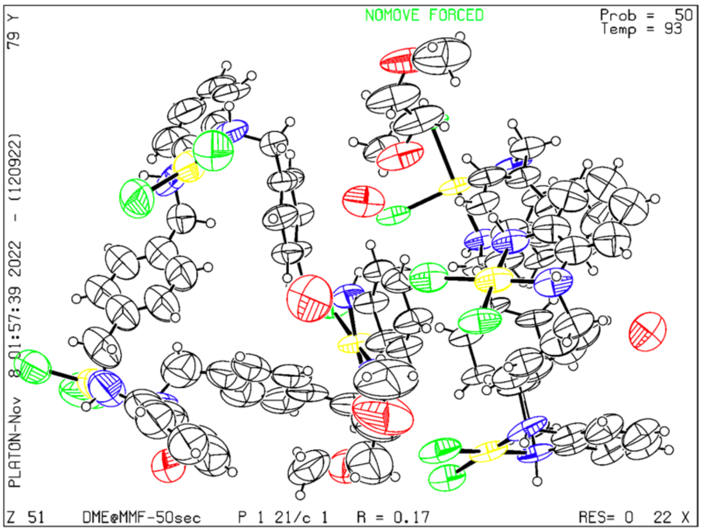

Supplementary Fig. 97 ORTEP drawing of the structurally extended MMF soaked in **DME for 50 seconds** at the 50% probability level. Color: C black, N blue, O red, Cl green and Pd yellow. This figure was produced by the checkCIF report of the International Union of Crystallography.

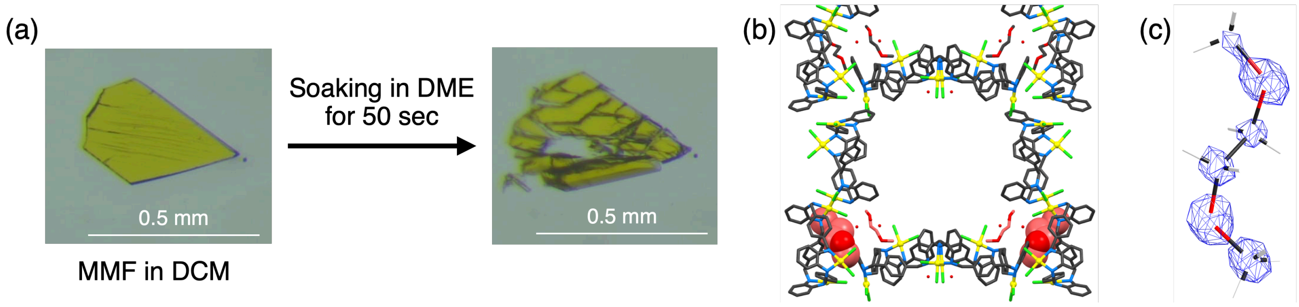

Supplementary Fig. 98 (a) Microscopic observation of MMF soaked in DME. (b) The unit-space structure of the extended MMF soaked in DME for 50 sec. (c) The electron density map of DME (100% occupancy) adsorbed in the allosteric binding site (contour level; 1.8 $eÅ^{-3}$).

-ACP (*soaking at 20 °C*)

Soaking procedure: As-crystalised MMF crystals were firstly soaked in dichloromethane in order to remove acetonitrile from the supernatant. Afterwards, the crystals were transferred into ACP to be soaked at 20 °C for 1 day, and one of the crystals was picked up and immediately mixed with paratone oil to measure single-crystal XRD.

Crystal data for $(Pd_3\mathbf{L}Cl_6)_2\cdot(\mathbf{ACP})_{0.82}\cdot(H_2O)_{2.5}$: $C_{90.43}H_{90.43}Cl_{12}N_{12}O_{3.30}Pd_6$, F_w = 2461.94, crystal dimensions 0.26 × 0.09 × 0.06 mm^3, monoclinic, space group $P2_1/c$, a = 19.6401(2), b = 52.8625(6), c = 14.21720(12) Å, β = 91.3058(9)°, V = 14756.8(3) $Å^3$, Z = 4, ρ_{calcd} = 1.108 g cm^{-3}, μ = 8.060 mm^{-1}, T = 93 K, λ(CuKα) = 1.54187 Å, $2\theta_{max}$ = 136.5°, 73525/ 26913 reflections collected/unique (R_{int} = 0.0336), R_1 = 0.0934 ($I > 2\sigma(I)$), wR_2 = 0.2851 (for all data), GOF = 1.093, largest diff. peak and hole 3.229/–2.818 $eÅ^{-3}$. CCDC deposit number 2223948

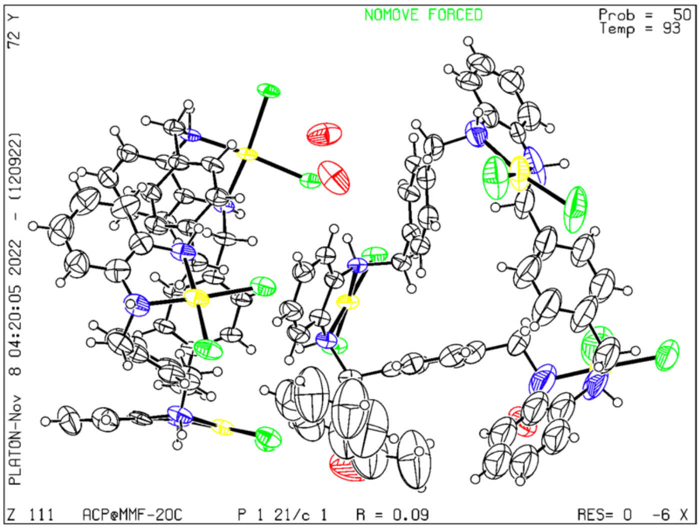

Supplementary Fig. 99 ORTEP drawing of not-extended MMF soaked in **ACP at 20 °C** at the 50% probability level. Color: C black, N blue, O red, Cl green and Pd yellow. This figure was produced by the checkCIF report of the International Union of Crystallography.

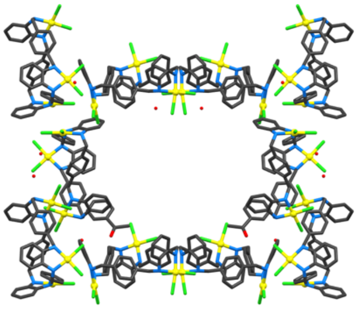

Supplementary Fig. 100 The unit-space structure of not-extended MMF soaked in ACP at 20 °C. Adsorption of ACP was observed on the void space of MMF channel, while water molecules were accommodated in the allosteric site.

<u>•Reversibility</u>

Soaking procedure: As-crystalised MMF crystals were soaked in 1,2-dimethoxyethane (DME) in order to induce the lattice expansion. Afterwards, the crystals were transferred into acetonitrile (MeCN) to be soaked at 20 °C for 10 min, and one of the crystals was picked up and immediately mixed with paratone oil to measure single-crystal XRD.

Crystal data for $(Pd_3\mathbf{L}Cl_6)_2\cdot(\mathbf{MeCN})_{2.44}\cdot(H_2O)_4$: $C_{88.90}H_{91.36}Cl_{12}N_{14.45}O_4Pd_6$, F_w = 2490.11, crystal dimensions 0.09 × 0.04 × 0.03 mm^3, monoclinic, space group $P2_1/c$, a = 19.6224(4), b = 52.0467(13), c = 14.2595(3) Å, β = 90.8645(18)°, V = 14561.3(6) Å^3, Z = 4, ρ_{calcd} = 1.136 g cm^{-3}, μ = 8.182 mm^{-1}, T = 93 K, λ(CuKα) = 1.54187 Å, $2\theta_{max}$ = 136.5°, 63196/ 26287 reflections collected/unique (R_{int} = 0.0829), R_1 = 0.1185 ($I > 2\sigma(I)$), wR_2 = 0.3588 (for all data), GOF = 1.167, largest diff. peak and hole 2.486/–2.398 eÅ^{-3}. CCDC deposit number 2223949

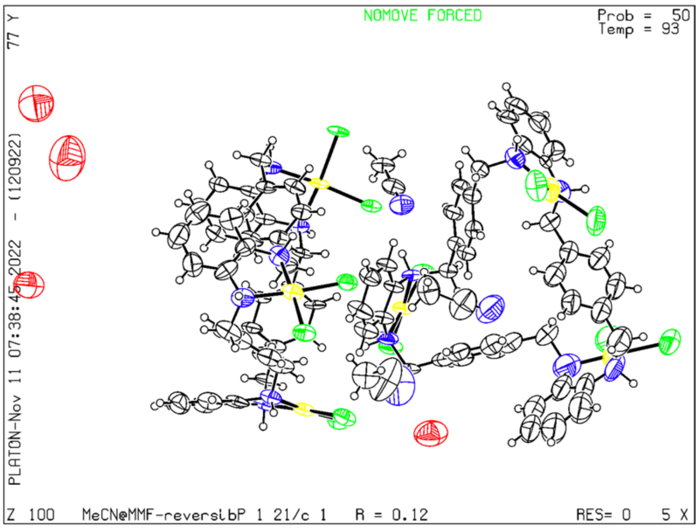

Supplementary Fig. 101 ORTEP drawing of the structurally contracted MMF soaked in **MeCN after in DME** at the 50% probability level. Color: C black, N blue, O red, Cl green and Pd yellow. This figure was produced by the checkCIF report of the International Union of Crystallography.

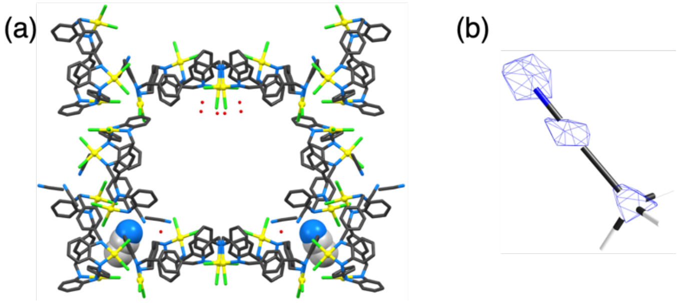

Supplementary Fig. 102 (a) The unit-space structure of the structurally contracted MMF soaked in MeCN after in DME, (b) The electron density map of MeCN (100% occupancy) adsorbed in the allosteric binding site (contour level; 4.0 eÅ^{-3}).

4. Principal component analysis of MMF crystal structures

Principal component analysis (PCA) of the crystal structures (Supplementary Figs. 1–96) was conducted for their characterization. It used six standardized interatomic distances[a] of N/C and Cl, which can potentially form NH…Cl hydrogen bonding or π–Cl interactions. For the crystals with space group $P2_1$ (MMF ⊃ 1,4-dioxane, MMF ⊃ 1,3-dioxolane), the arithmetic mean of (*P*/*M*)-sides was used. The main contribution to the first principal component PC1 comes from the four interatomic distance (d_2, d_3, d_5, d_6) between *syn*-1…*syn*-2 and *syn*-2…*syn*-3 complexes, correlating to the positional difference of the crystal components (*syn*-1, *syn*-2 and *syn*-3) in the crystallographic ac-plane (Supplementary Fig. 104a,c). The main contribution to the second principal component PC2 comes from the two interatomic distances (d_1, d_4) between *anti*…*syn*-3 and *syn*-2…*syn*-3 complexes, correlating to the positional difference of the crystal components (*syn*-2, *syn*-3 and *anti*) in the crystallographic ab-plane (Supplementary Fig. 104a,d). The contribution ratio was 67.6 % for PC1, and 29.3% for PC2, respectively. Resulting PCA score plot showed that all reported crystal structures naturally separate into three clusters on the (PC1, PC2) plane.

a…The measured distance values are centred and divided by the standard deviation of all the observations of each distance.

$$z_{ij} = \frac{(d_{ij} - \bar{d_j})}{s_j}$$

z_{ij}; the standardized distance (i; the indicator of the crystal structures, j; the indicator of the interatomic pairs), d_{ij}; the measured distance values d_j of the i-th crystal structure. s_j; the standard deviation of all the observations of each distance d_j.

		*d*1	*d*2	*d*3	*d*4	*d*5	*d*6	PC 1	PC 2
		anti–syn3	*syn1–syn2*			*syn2–syn3*			
		N–Cl	N–Cl	C–Cl	N–Cl	Cl–N	Cl–N		
1	MeCN	3.295	3.267	4.688	3.233	5.467	5.038	-1.6967961	-1.2884948
2	1,4-Dioxane	3.3895	3.2105	4.4855	3.245	5.787	5.306	-2.2154176	-2.5932164
3	IPA	3.368	3.27	4.304	3.297	5.724	5.166	-2.124948	-2.2457552
4	SBA	3.388	3.251	4.388	3.282	5.674	5.156	-2.1350995	-2.553604
5	SPA	3.376	3.231	4.335	3.277	5.747	5.195	-2.1638414	-2.3791954
6	1,3-Dioxolane	3.33	3.2695	4.5225	3.248	5.586	5.0775	-1.8858349	-1.7657111
7	Water	3.295	3.313	4.796	3.231	5.304	4.839	-1.5236704	-1.3177337
8	EtOH	3.272	3.268	4.58	3.273	5.597	5.112	-1.7430735	-0.9026359
9	Et2O	3.22	3.27	4.979	3.157	5.601	5.154	-1.5118646	-0.2673642
10	AcOEt	3.312	3.208	4.658	3.245	5.573	5.15	-1.8418358	-1.5072835
11	PEA	3.339	3.178	4.125	3.29	6.059	5.472	-2.3546725	-1.7908703
12	EG	3.286	3.364	4.573	3.289	5.552	5.039	-1.7138784	-1.0917801
13	PD	3.343	3.288	4.4	3.307	5.692	5.11	-1.9990938	-1.8831206
14	BnOH(II)	3.216	3.243	3.356	5.423	7.067	6.607	-3.1383181	2.28978597
15	FBA (II)	3.219	3.221	3.37	5.453	7.044	6.634	-3.1519523	2.27683669
16	MOBA (II)	3.235	3.204	3.323	5.511	7.122	6.676	-3.2548338	2.11221598
17	MBA (II)	3.212	3.238	3.435	5.5	7.074	6.648	-3.1299857	2.42834625
18	BBA (II)	3.22	3.231	3.49	5.411	7.036	6.605	-3.1021858	2.21713389
19	PhOH (II)	3.253	3.278	3.479	5.618	7.045	6.714	-3.2332315	1.96469728
20	p-cresol (II)	3.247	3.276	3.332	5.731	7.092	6.753	-3.2967455	2.170913
21	BnOH(III)	3.285	7.206	7.628	4.34	3.22	3.256	1.50477932	-0.0961365
22	FBA (III)	3.312	7.188	7.671	4.355	3.198	3.253	1.45220136	-0.4722868
23	MOBA (III)	3.261	7.202	7.603	4.198	3.217	3.27	1.56042251	0.10843912
24	MBA (III)	3.273	7.15	7.626	4.329	3.172	3.242	1.54236111	0.05778338
25	BBA (III)	3.253	7.104	7.587	4.226	3.174	3.26	1.56824783	0.24171987
26	PhOH (III)	3.278	7.352	7.804	4.094	3.259	3.275	1.60111825	-0.2280579
27	p-cresol (III)	3.269	6.92	7.478	4.131	3.185	3.257	1.45470803	-0.0948366
28	cyclohexanol	3.233	7.462	7.757	4.285	3.128	3.277	1.76024244	0.61095093
29	ACAC	3.264	7.392	7.904	4.408	3.173	3.242	1.6980551	0.28114191
30	FA	3.255	7.246	7.652	4.48	3.197	3.221	1.61017642	0.47271733
31	NB	3.247	7.243	7.668	4.358	3.23	3.208	1.63486105	0.46594585
32	PhCN	3.294	7.206	7.664	4.231	3.228	3.217	1.51082106	-0.3383903
33	pentaglyme	3.249	7.121	7.651	4.206	3.194	3.245	1.60071389	0.2795312
34	tetraglyme	3.25	7.091	7.634	4.146	3.19	3.288	1.57391386	0.2077715
35	ACP	3.279	7.08	7.59	4.239	3.249	3.248	1.47700684	-0.1183402
36	cyclohexanone	3.216	7.063	7.465	3.948	3.204	3.35	1.58723877	0.50531383
37	triglyme	3.249	7.032	7.568	4.084	3.172	3.278	1.55660295	0.15458488
38	DME	3.272	6.795	7.396	3.793	3.265	3.345	1.35118986	-0.4698772
39	diglycol	3.277	7.102	7.531	4.062	3.203	3.324	1.4664635	-0.2586902
40	triglycol	3.236	7.184	7.674	4.388	3.176	3.261	1.64506865	0.65315913
41	teraglycol	3.254	7.286	7.78	4.424	3.168	3.239	1.66409081	0.43380275
42	pentaglycol	3.237	7.132	7.631	4.322	3.182	3.25	1.62357046	0.56869443
43	hexaglycol	3.254	7.27	7.749	4.406	3.163	3.246	1.65138079	0.41539665
44	1,4-butanediol	3.266	7.049	7.505	4.147	3.176	3.266	1.50042829	-0.0267044
45	resorcinol	3.273	7.092	7.564	4.091	3.244	3.301	1.47700433	-0.1734263
46	mequinol	3.233	7.085	7.462	3.996	3.195	3.289	1.57327441	0.30315436
47	BHC	3.214	8.42	8.661	5.147	3.43	3.331	2.12984629	1.82625673
48	Glycerol	3.299	6.629	7.18	4.187	3.239	3.334	1.17291253	-0.4774496
49	MPD	3.211	6.936	7.471	3.971	3.238	3.347	1.55853632	0.593095
50	Pyranol	3.236	7.371	7.709	4.426	3.148	3.257	1.71004215	0.70157354

Supplementary Fig. 103 The measured six interatomic distance values (Å) and the principal component scores of 50 kinds of MMF structures accommodating effector molecules. The first principal component (PC1) is the linear combination of six standardised interatomic distance variables that has maximum variance of the 50 data

sets. The sum of the squared coefficients of six variables is fixed to be one. The second principal component (PC2) is the linear combination of the variables that has maximum variance under the constraint that the correlation with PC1 is zero, and that the sum of the squared coefficients of six variables is one.

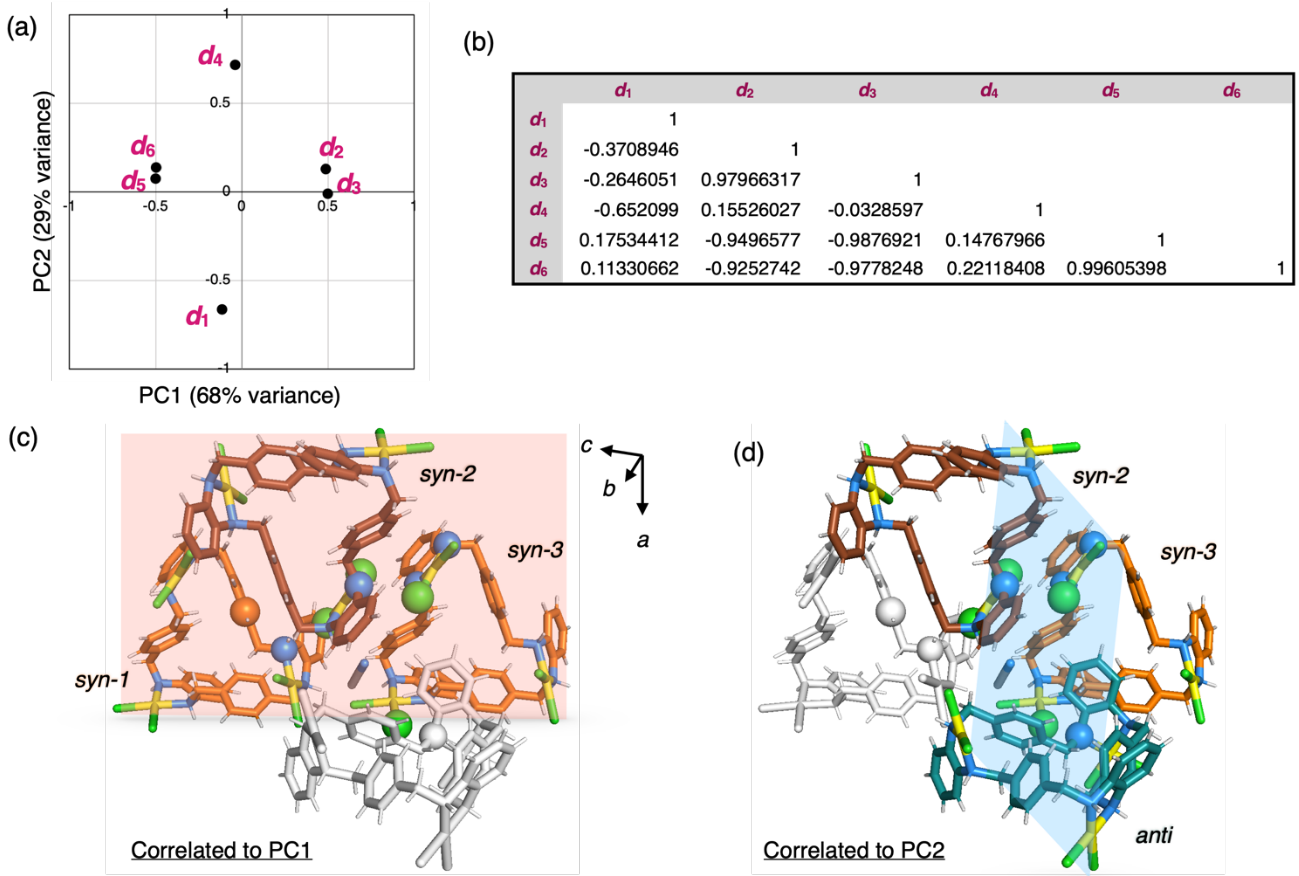

	d_1	d_2	d_3	d_4	d_5	d_6
d_1	1					
d_2	-0.3708946	1				
d_3	-0.2646051	0.97966317	1			
d_4	-0.652099	0.15526027	-0.0328597	1		
d_5	0.17534412	-0.9496577	-0.9876921	0.14767966	1	
d_6	0.11330662	-0.9252742	-0.9778248	0.22118408	0.99605398	1

Supplementary Fig. 104 (a,b) (a) PCA loading plot and (b) the correlation matrix of the standardised interatomic distance. (c,d) The crystal components whose intermolecular positional difference contributed to each principal component. (c) The first principal component PC1 is mainly correlated to the positional difference of the crystal components (*syn*-1, *syn*-2 and *syn*-3) in the crystallographic ac-plane, and (d) the second principal component PC2 is correlated to the positional difference of the crystal components (*syn*-2, *syn*-3 and *anti*) in the crystallographic ab-plane.

5. Powder X-ray diffraction analysis of MMF crystal transformation

<u>Powder X-ray diffraction analysis of MMF in ethers</u>

Preparation: MMF crystals soaked in each solvent (MeCN, DME, 1,4-dioxane) were transferred into a glass capillary with a solvent to measure powder X-ray diffraction (PXRD) at rt. The total soaking time in DME or in 1,4-dioxane was 10 min before the samples were subject to X-ray irradiation.

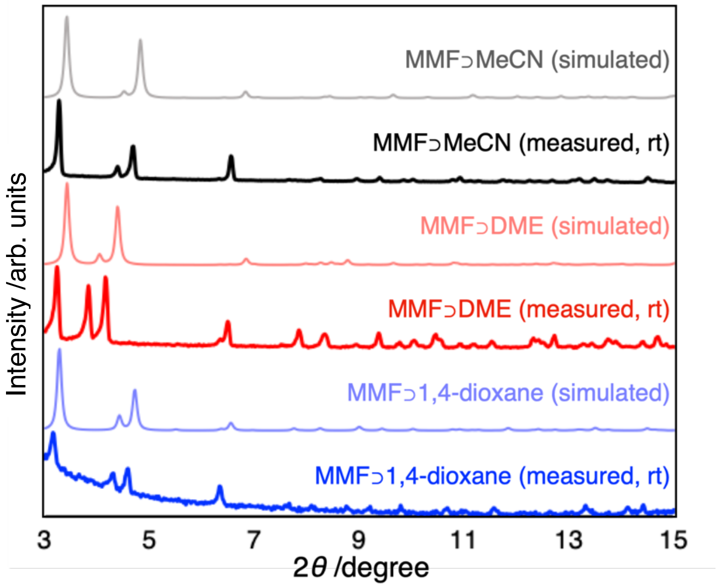

Supplementary Fig. 105 Powder X-ray diffraction patterns (CuKα, rt) of MMF dispersed in MeCN, DME and 1,4-dioxane with simulated patterns from the crystal structures. The soaking time in DME or in 1,4-dioxane was 10 min. Due to the thermal expansion of crystals, the peaks shifted to the lower angle compared to the simulated patterns.

<u>Powder X-ray diffraction analysis of reversible transformation</u>

Procedure: Firstly as-crystalised MMF was dispersed in MeCN in a vial. The supernatant was replaced by an ethereal solvent (DME/1,4-dioxane). After soaking for 1 h, a small amount of the crystals was picked and transferred into a capillary with the solvent, to be subject to powder X-ray diffraction analysis. Next, the remaining solvent in a vial was replaced by MeCN. After soaking for 1 h, small amount of the crystals was picked and transferred into another capillary with the solvent, to be subject to powder X-ray diffraction analysis. This solvent exchange and crystal transfer procedure were repeatedly operated up to four cycles and the prepared eight samples are subject to X-ray irradiation, respectively.

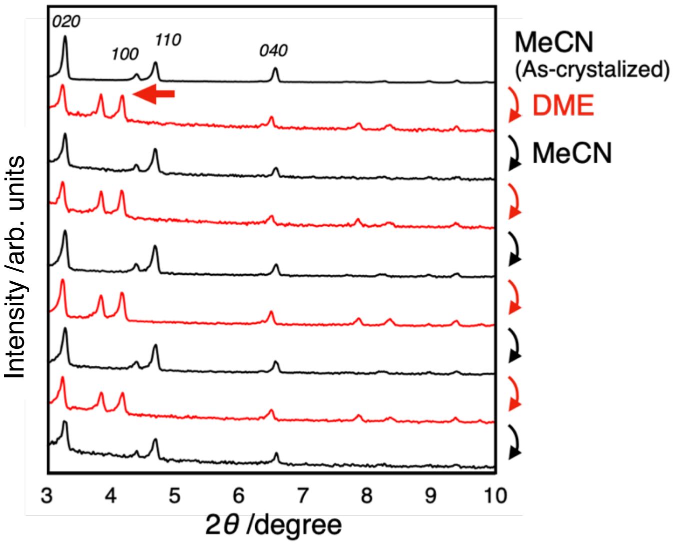

Supplementary Fig. 106 Powder X-ray diffraction patterns (CuKα, rt) of MMF repeatedly soaked in MeCN and DME.

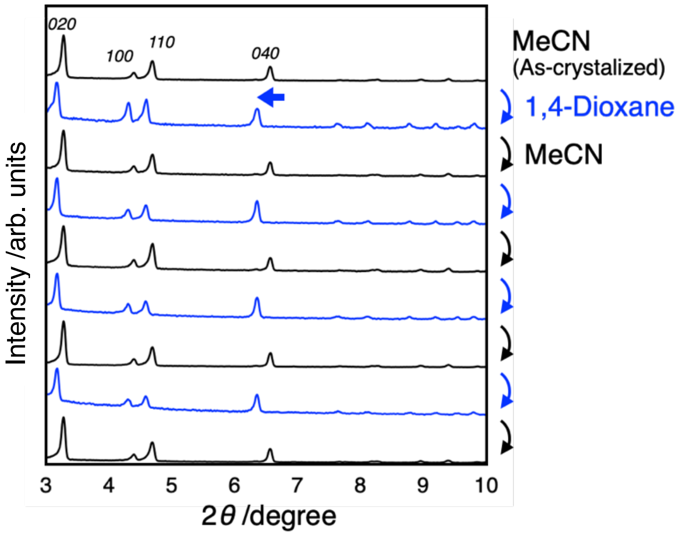

Supplementary Fig. 107 Powder X-ray diffraction patterns (CuKα, rt) of MMF repeatedly soaked in MeCN and 1,4-dioxane.

Powder X-ray diffraction analysis of MMF transformation with various effectors

•Structural extension (Cluster III) in neat oil of effectors at 20 °C

Procedure: MMF crystals were soaked in each solvent (triethyleneglycol dimethyl ether, tetraethyleneglycol dimethyl ether, pentaethyleneglycol dimethyl ether, acetylacetone, cyclohexanone, cyclohexanol, furfuryl alcohol and 1,1-bis(hydroxymethyl)cyclopropane) at 20 °C for 1 day. Afterwards, they were transferred into a glass capillary with the solvent to measure powder X-ray diffraction at room temperature. For most of linear diols and oligoethylene glycols, sample preparation was technically difficult due to their high viscosity.

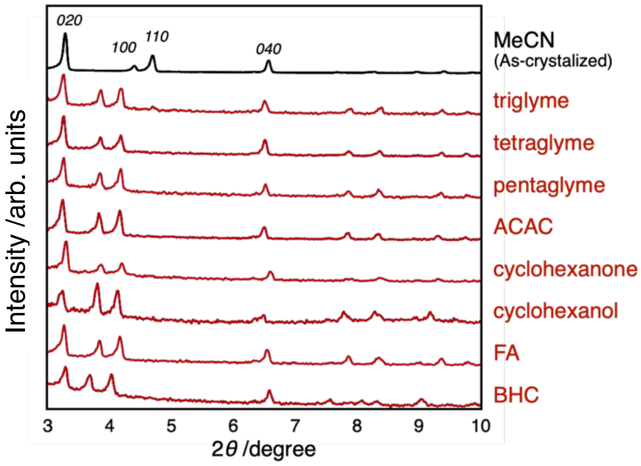

Supplementary Fig. 108 Powder X-ray diffraction patterns (CuKα, rt) of MMF crystals soaked in

triethyleneglycol dimethyl ether (triglyme), tetraethyleneglycol dimethyl ether (tetraglyme), pentaethyleneglycol dimethyl ether (pentaglyme), acetylacetone (ACAC), cyclohexanone, cyclohexanol, furfuryl alcohol (FA) and 1,1-bis(hydroxymethyl)cyclopropane (BHC).

•Transformation in acetophenone (ACP) by heating

Procedure:

[**20 °C (1 d)**] MMF crystals were soaked in acetophenone at 20 °C for 1 day.

[**100 °C (3 h)**] MMF crystals soaked in acetophenone were heated at 100 °C for 3 h.

[**DME then ACP**] MMF crystals were firstly soaked in DME to induce the lattice expansion. Afterwards, the crystals were transferred into acetophenone to be soaked at 20 °C for 1 day.

For all three samples, the crystals were transferred into a glass capillary with acetophenone to measure powder X-ray diffraction at room temperature.

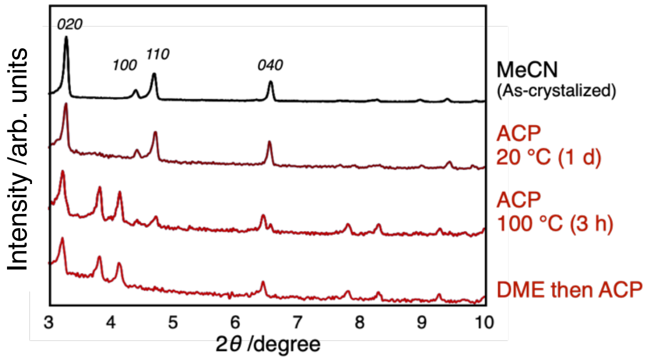

Supplementary Fig. 109 Powder X-ray diffraction patterns (CuKα, rt) of MMF crystals soaked in acetophenone (ACP). While soaking at 20 °C for 1 day did not afford transformation (second row), heating at 100 °C for 3 h afforded the extended structure (third row). By soaking in DME before in ACP, the extended structure was obtained as well (bottom).

•Transformation in benzonitrile (PhCN) by heating

Procedure:

[**20 °C (1 d)**] MMF crystals were soaked in benzonitrile at 20 °C for 1 day.

[**70 °C (1 d)**] MMF crystals soaked in benzonitrile were heated at 70 °C for 1 day.

For both samples, the crystals were transferred into a glass capillary with benzonitrile to measure powder X-ray diffraction at room temperature.

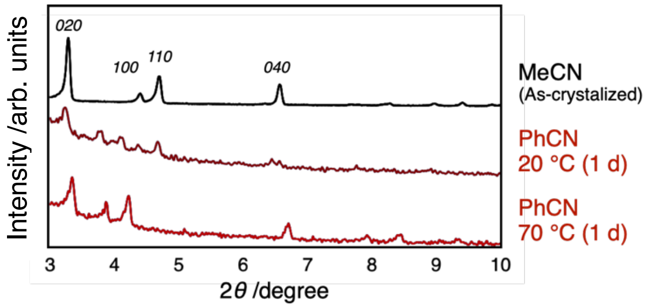

Supplementary Fig. 110 Powder X-ray diffraction patterns (CuKα, rt) of MMF crystals soaked in benzonitrile (PhCN). While soaking at 20 °C afforded both contracted and extended structure (middle), heating at 70 °C afforded the extended structure with high purity (bottom).

•Transformation in nitrobenzene (NB) by heating

Procedure:

[**20 °C (1 d)**] MMF crystals were soaked in nitrobenzene at 20 °C for 1 day.

[**70 °C (1 d)**] MMF crystals soaked in nitrobenzene were heated at 70 °C for 1 day.

For both samples, the crystals were transferred into a glass capillary with nitrobenzene to measure powder X-ray diffraction at rt.

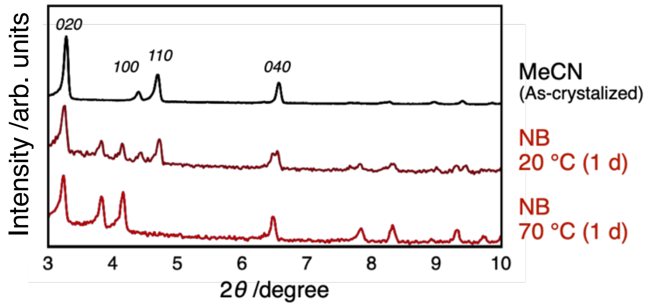

Supplementary Fig. 111 Powder X-ray diffraction patterns (CuKα, rt) of MMF crystals soaked in nitrobenzene (NB). While soaking at 20 °C afforded both contracted and extended structure (middle), heating at 70 °C afforded the extended structure with high purity (bottom).

•Transformation in solution of resorcinol

Procedure: MMF crystals were soaked in solution of resorcinol at 20 °C for 1 day. A 9:1 (v:v) mixture of 1,2,3,4-tetrahydronaphthalene and acetone, or just acetone were used as solvents. For all samples, the crystals were transferred into a glass capillary with the solution to measure powder X-ray diffraction at rt.

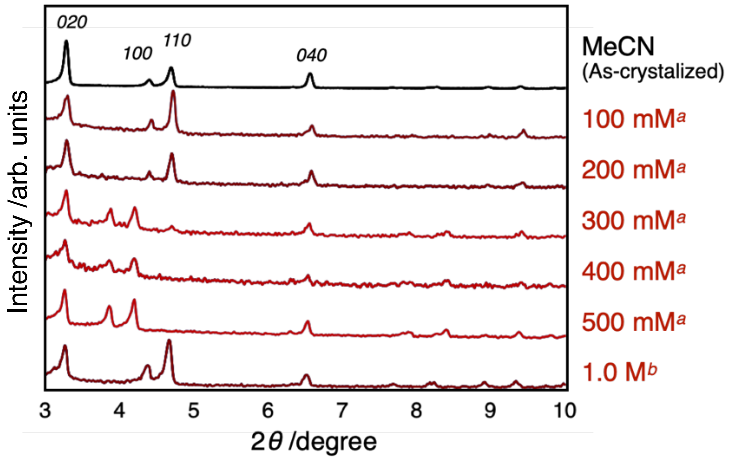

Supplementary Fig. 112 Powder X-ray diffraction patterns (CuKα, rt) of MMF crystals soaked in a solution

of resorcinol. *a*: a 9:1 (v:v) mixture of 1,2,3,4-tetrahydronaphthalene (tetralin) and acetone was used a solvent. *b*: Acetone was used a solvent. In the tetralin/acetone mixed solution, the extended structure was obtained with high purity in more than 300 mM solution.

•Transformation in a solution of mequinol

Procedure: MMF crystals were soaked in a 1,2,3,4-tetrahydronaphthalene:acetone = 9:1 (v:v) solution of mequinol at 20 °C for 1 day. The crystals were transferred into a glass capillary with the solution to measure powder X-ray diffraction at room temperature.

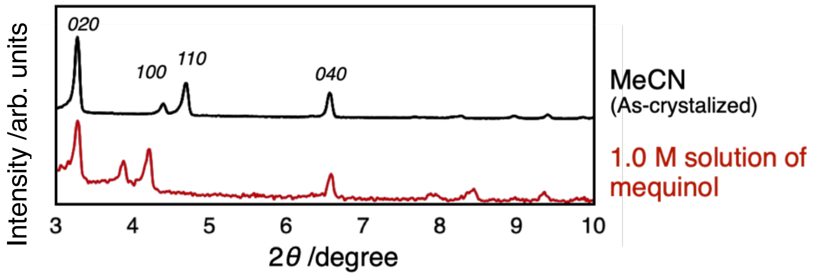

Supplementary Fig. 113 Powder X-ray diffraction patterns (CuKα, rt) of MMF crystals soaked in a 1.0 M solution of mequinol. 9:1 (v:v) mixture of 1,2,3,4-tetrahydronaphthalene (tetralin) and acetone was used a solvent.

•Structural extension (Cluster I) in neat oil of effectors at 20 °C

Procedure: MMF crystals were soaked in each solvent (isopropanol, (*rac*)-*sec*-butyl alcohol, (*rac*)-*sec*-pentyl alcohol, (*rac*)-1-phenylethanol, ethyl acetate) at 20 °C for 1 day. Afterwards, they were transferred into a glass capillary with the solvent to measure powder X-ray diffraction at room temperature. For most of linear diols and oligoethylene glycols, sample preparation was technically difficult due to their high viscosity.

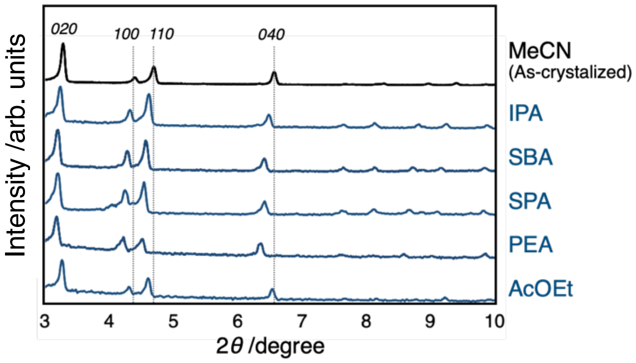

Supplementary Fig. 114 Powder X-ray diffraction patterns (CuKα, rt) of MMF crystals soaked in isopropanol (IPA), (*rac*)-*sec*-butyl alcohol (SBA), (*rac*)-*sec*-pentyl alcohol (SPA), (*rac*)-1-phenylethanol (PEA) and ethyl acetate (AcOEt).

•MMF in Et_2O

Procedure: MMF crystals were soaked in Et_2O at 20 °C for 1 day. Afterwards, they were transferred into a glass capillary with Et_2O to measure powder X-ray diffraction at rt.

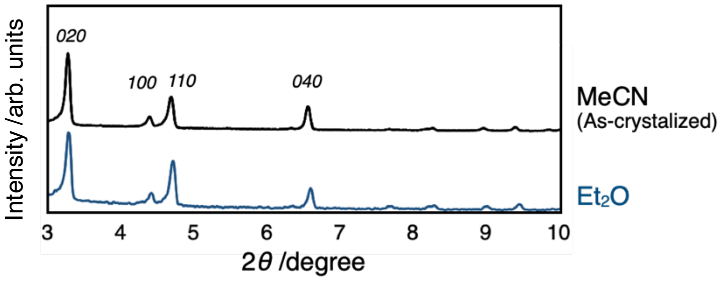

Supplementary Fig. 115 Powder X-ray diffraction patterns (CuKα, rt) of MMF crystals soaked in Et_2O. No transformation was observed, indicating that cooling to the cryogenic condition is necessary for the structural contraction.

•Stepwise transformation with benzyl alcohol (BnOH)

Procedure:

[**20 °C (1 d)**] MMF crystals were soaked in benzyl alcohol at 20 °C for 1 day.

[**100 °C/125 °C (10 min)**] MMF crystals soaked in benzyl alcohol were heated at 100 or 125 °C for 10 min.

[**DME then BnOH**] MMF crystals were firstly soaked in DME to induce the lattice expansion. Afterwards, the crystals were transferred into benzyl alcohol to be soaked at 20 °C for 1 day.

For all four samples, the crystals were transferred into a glass capillary with BnOH to measure powder X-ray diffraction at room temperature.

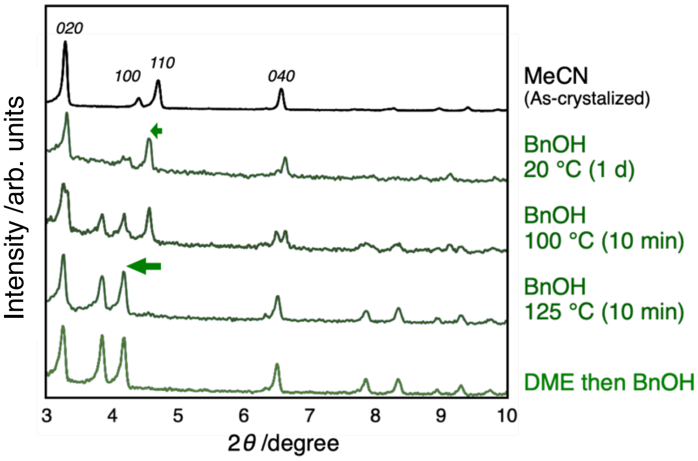

Supplementary Fig. 116 Powder X-ray diffraction patterns (CuKα, rt) of MMF crystals soaked in benzyl alcohol (BnOH). After soaking at 20 °C for 1 day, the moderately-extended structure was obtained (second row). By heating the crystals, further transformation to the significantly-extended structure was observed (third and fourth row). By soaking in DME before in BnOH, the significantly-extended structure was obtained as well (bottom).

•Stepwise transformation with 2-fluorobenzyl alcohol (FBA)

Procedure:

[**20 °C (1 d/2 d)**] MMF crystals were soaked in 2-fluorobenzyl alcohol at 20 °C for 1 or 2 days.

[**125 °C (10 min)**] MMF crystals soaked in 2-fluorobenzyl alcohol were heated at 125 °C for 10 min.

[**DME then FBA**] MMF crystals were firstly soaked in DME to induce the lattice expansion. Afterwards, the crystals were transferred into 2-fluorobenzyl alcohol to be soaked at 20 °C for 1 day.

For all four samples, the crystals were transferred into a glass capillary with FBA to measure powder X-ray diffraction at rt.

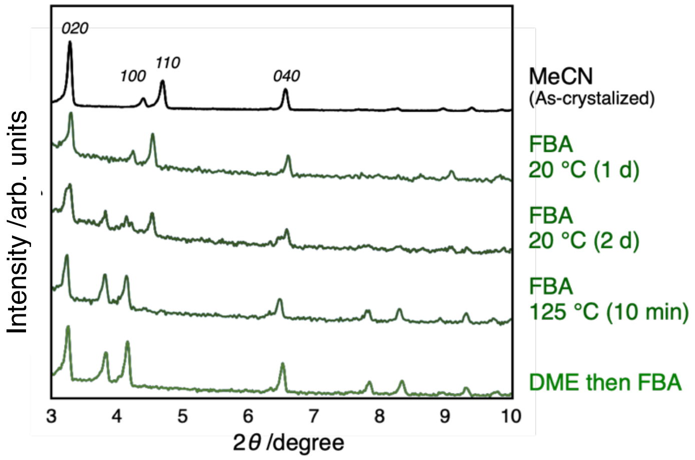

Supplementary Fig. 117 Powder X-ray diffraction patterns (CuKα, rt) of MMF crystals soaked in 2-fluorobenzyl alcohol (FBA). After soaking at 20 °C for 1 day, the moderately-extended structure was obtained (second row). Further transformation to the significantly-extended structure started by soaking for 2 days, and completed by heating (third and fourth row). By soaking in DME before in FBA, the significantly-extended structure was obtained as well (bottom).

•Stepwise transformation with 3-methoxybenzyl alcohol (MOBA)

Procedure:

MMF crystals were soaked in 3-methoxybenzyl alcohol at 20 °C for 1 or 2 days.

For two samples, the crystals were transferred into a glass capillary with MOBA to measure powder X-ray diffraction at room temperature.

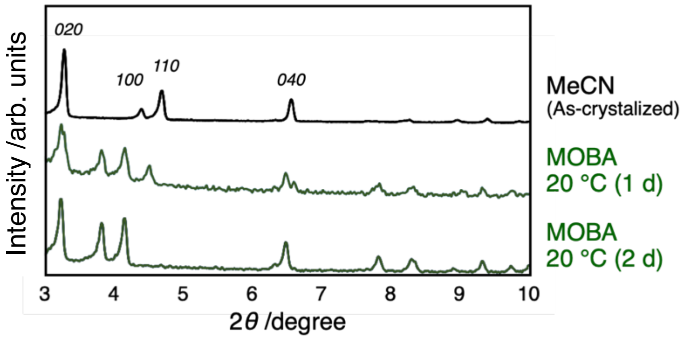

Supplementary Fig. 118 Powder X-ray diffraction patterns (CuKα, rt) of MMF crystals soaked in 3-methoxybenzyl alcohol (MOBA). After soaking at 20 °C for 1 day, the mixture of the moderately-extended and the significantly-extended structure was obtained (middle). The significantly-extended structure was obtained with high purity in 2 days (bottom).

•Two different MMF crystal structures with 3-methylbenzyl alcohol (MBA)

Procedure:

[**MBA**] MMF crystals were soaked in 3-methylbenzyl alcohol at 20 °C for 1 day.

[**DME then MBA**] MMF crystals were firstly soaked in DME to induce the lattice expansion. Afterwards, the crystals were transferred into 3-methylbenzyl alcohol to be soaked at 20 °C for 1 day.

For two samples, the crystals were transferred into a glass capillary with MBA to measure powder X-ray diffraction at rt.

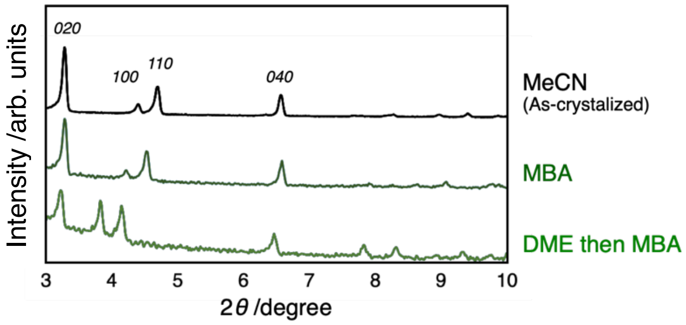

Supplementary Fig. 119 Powder X-ray diffraction patterns (CuKα, rt) of MMF crystals soaked in 3-methylbenzyl alcohol (MBA). After soaking at 20 °C for 1 day, the moderately-extended structure was obtained (middle). By soaking in DME before in MBA, the significantly-extended structure was obtained (bottom). Heating treatment induced the crystal degradation before the interconversion between these two structures.

• Two different MMF crystal structures in solution of phenol (PhOH)

Procedure:

[**PhOH/tetralin**] MMF crystals were soaked in a 1,2,3,4-tetrahydronaphthalene (tetralin) solution of phenol (300 mM, 1.0 M) at 20 °C for 1 day.

[**DME then PhOH/tetralin**] MMF crystals were firstly soaked in DME to induce the lattice expansion. Afterwards, the crystals were transferred into tetralin solution of phenol (300 mM, 1.0 M) to be soaked at 20 °C for 1 day.

For four samples, the crystals were transferred into a glass capillary with solution to measure powder X-ray diffraction at room temperature.

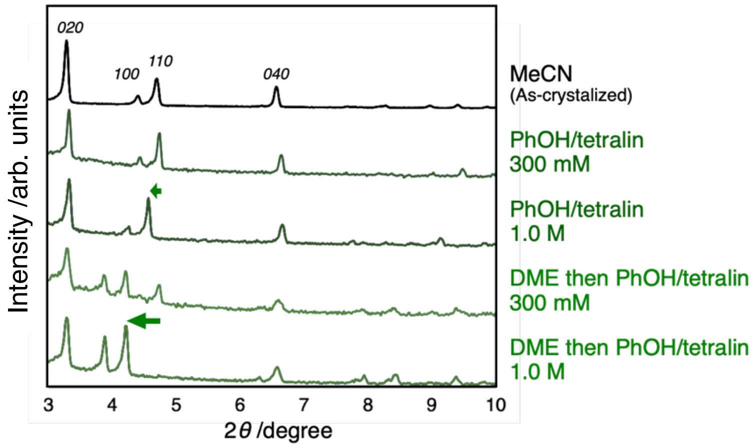

Supplementary Fig. 120 Powder X-ray diffraction patterns (CuKα, rt) of MMF crystals soaked in a 1,2,3,4-tetrahydronaphthalene (tetralin) solution of phenol (PhOH). By soaking in 1.0 M solution, the moderately-extended structure was obtained (second and third row). By soaking in DME before in a 1.0 M solution of PhOH, the significantly-extended structure was obtained (fourth and bottom row). Heating treatment induced the crystal degradation before the interconversion between these two structures.

• Two different MMF crystal structures in solution of *p*-cresol

Procedure:

[***p*-Cresol/tetralin**] MMF crystals were soaked in a 1,2,3,4-tetrahydronaphthalene (tetralin) solution of *p*-cresol (1.0, 2.0 M) at 20 °C for 1 day.

[**DME then *p*-cresol/tetralin**] MMF crystals were firstly soaked in DME to induce the lattice expansion. Afterwards, the crystals were transferred into a tetralin solution of *p*-cresol (1.0, 2.0 M) to be soaked at 20 °C for 1 day.

For four samples, the crystals were transferred into a glass capillary with solution to measure powder X-ray diffraction at rt.

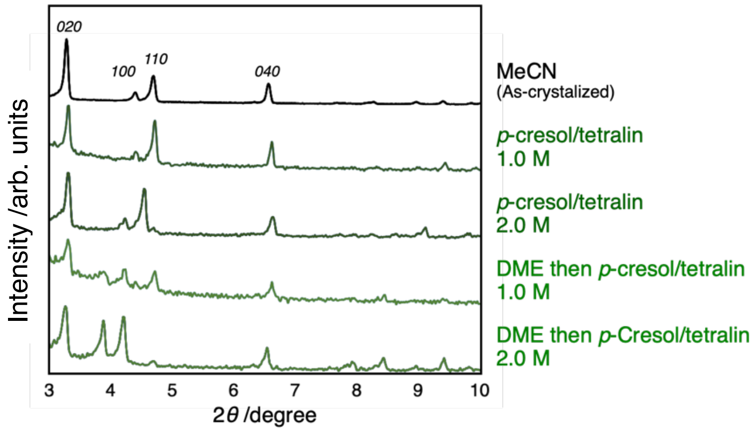

Supplementary Fig. 121 Powder X-ray diffraction patterns (CuKα, rt) of MMF crystals soaked in a 1,2,3,4-tetrahydronaphthalene (tetralin) solution of *p*-cresol. By soaking in 2.0 M solution, the moderately-extended structure was obtained (second and third row). By soaking in DME before in a 2.0 M solution of *p*-cresol, the

significantly-extended structure was obtained (fourth and bottom row). Heating treatment induced the crystal degradation before the interconversion between these two structures.

Powder X-ray diffraction analysis of MMF transformation in solution of effectors

•In solution of DME

Procedure: MMF crystals were soaked in a solution of DME at 20 °C for 1 day. Acetone, toluene and 1,2,3,4-tetrahydronaphthalene (tetralin) were used as solvents. For all samples, the crystals were transferred into a glass capillary with solution to measure powder X-ray diffraction at room temperature.

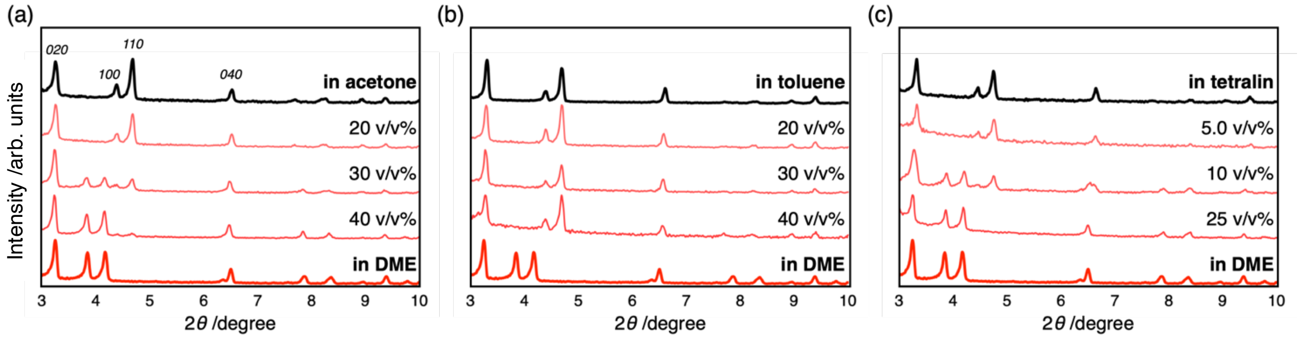

Supplementary Fig. 122 Powder X-ray diffraction patterns (CuKα, rt) of MMF crystals soaked in (a) acetone, (b) toluene, and (c) a 1,2,3,4-tetrahydronaphthalene (tetralin) solution of DME. In tetralin, the relatively effective transformation can be induced with the diluted condition.

•In a solution of furfuryl alcohol (FA)

Procedure: MMF crystals were soaked in solution of furfuryl alcohol at 20 °C for 1 day. Acetone and 1,2,3,4-tetrahydronaphthalene (tetralin) were used as solvents. For all samples, the crystals were transferred into a glass capillary with solution to measure powder X-ray diffraction at rt.

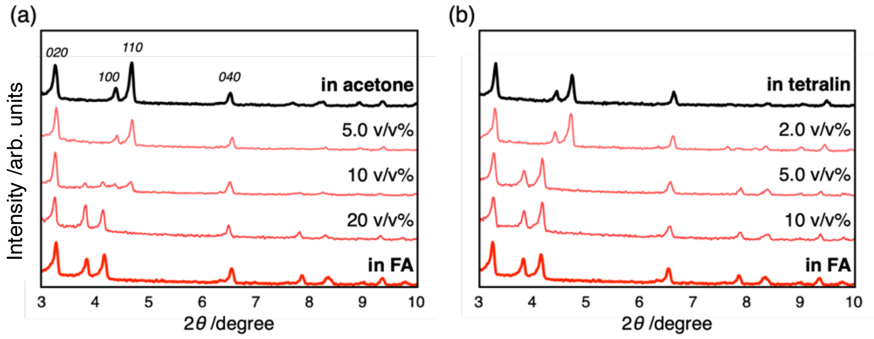

Supplementary Fig. 123 Powder X-ray diffraction patterns (CuKα, rt) of MMF crystals soaked in (a) acetone and (b) a 1,2,3,4-tetrahydronaphthalene (tetralin) solution of furfuryl alcohol (FA). Compared to DME, the more effective transformation can be induced, indicating the preferential accommodation.

•In acetone solution of effectors

Procedure: MMF crystals were soaked in a 30 v/v% acetone solution of acetylacetone, triethyleneglycol dimethyl ether, tetraethyleneglycol dimethyl ether, benzonitrile, cyclohexanone, cyclohexanol, 2-methyl-1,3-propanediol or 1-phenylethanol at 20 °C for 1 day. For all samples, the crystals were transferred into a glass capillary with solution to measure powder X-ray diffraction at rt.

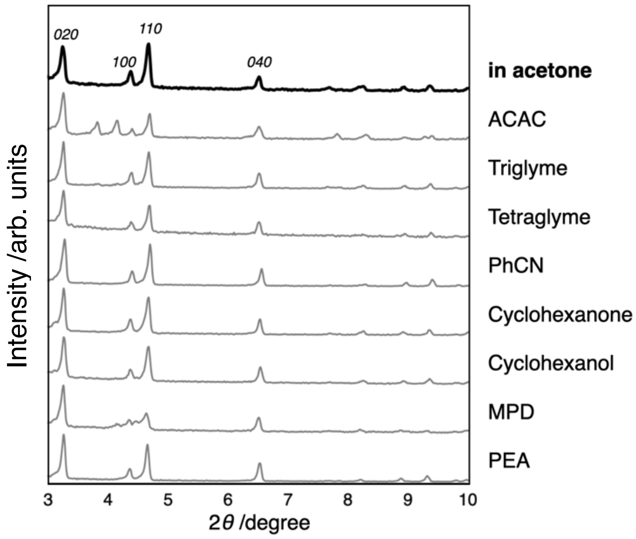

Supplementary Fig. 124 Powder X-ray diffraction patterns (CuKα, rt) of MMF crystals soaked in a 30 v/v% acetone solution of acetylacetone (ACAC), triethylene glycol dimethyl ether (triglyme), tetraethylene glycol dimethyl ether (tetraglyme), benzonitrile (PhCN), cyclohexanone, cyclohexanol, 2-methyl-1,3-propanediol (MPD) or 1-phenylethanol (PEA). No or just partial transformation can be induced.

<u>Effector competition experiments</u>

Procedure: MMF crystals were soaked in the mixture of two different effectors at 20 °C for 1 day. For all samples, the crystals were transferred into a glass capillary with solvents to measure powder X-ray diffraction at rt.

•MeCN and DME

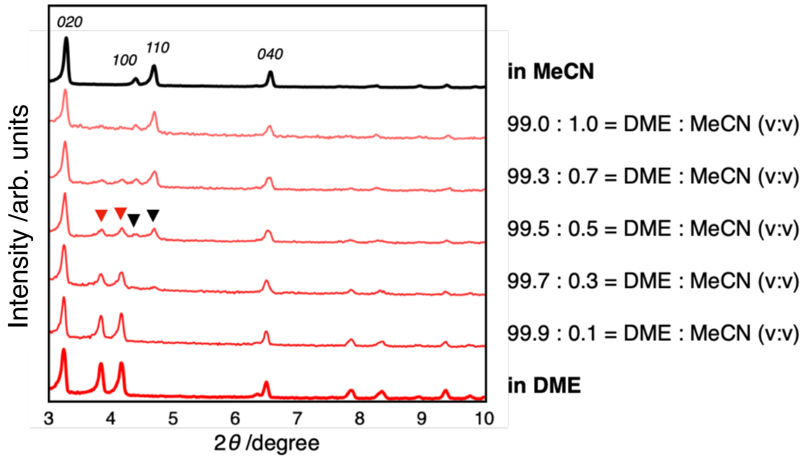

Supplementary Fig. 125 Powder X-ray diffraction patterns (CuKα, rt) of MMF crystals soaked in a mixture of MeCN and DME (different clusters). In the mixture of DME:MeCN = 99.3:0.7–99.7:0.3 (v:v), coexistence of two distinct crystal structures was observed, as indicated by the black and red triangles.

•MeCN and 1,4-dioxane

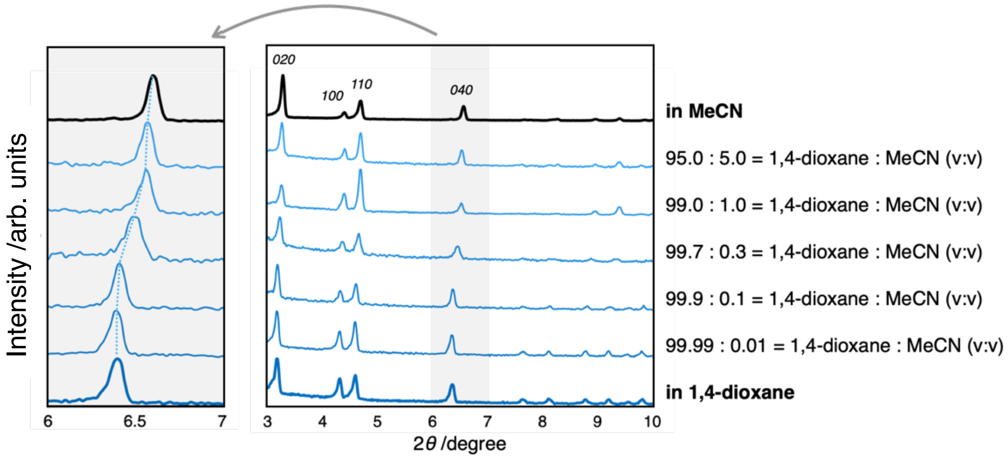

Supplementary Fig. 126 Powder X-ray diffraction patterns (CuKα, rt) of MMF crystals soaked in a mixture of MeCN and 1,4-dioxane (the same clusters). Gradual peak shift is observed, as indicated by blue dotted lines.

•MeCN and BnOH

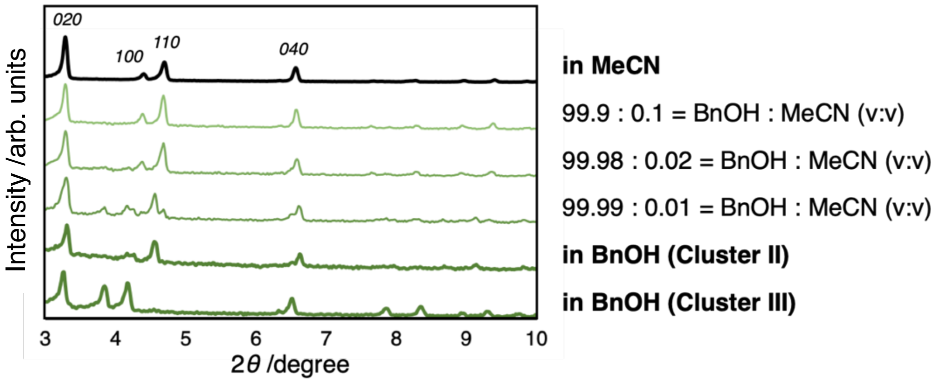

Supplementary Fig. 127 Powder X-ray diffraction patterns (CuKα, rt) of MMF crystals soaked in a mixture of MeCN and BnOH (different clusters). In a mixture of BnOH:MeCN = 99.99:0.01 (v:v), coexistence of three distinct crystal structures was suggested.

•DME and BHC

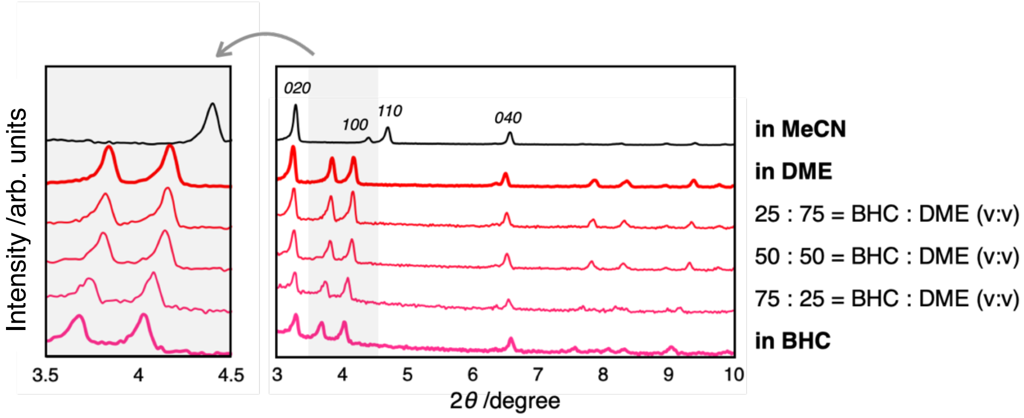

Supplementary Fig. 128 Powder X-ray diffraction patterns (CuKα, rt) of MMF crystals soaked in a mixture of DME and BHC (the same clusters). Gradual peak shift was observed.

6. Allosteric regulation of molecular recognition ability

•*trans*-Azobenzene (AB)

–in DME

Soaking procedure: MMF crystals were soaked in DME solution of ***trans*-azobenzene** (1.0 M) at 20 °C for 1 day, and one of the crystals was picked up and immediately mixed with paratone oil to measure single-crystal XRD. Other crystals were filtered and washed with 500 μL DME to remove the guest non-specifically adsorbed on the crystal surface, and then dissolved in DMSO-d_6/DCl-D_2O to determine the molar ratio of the protonated macrocycle ligand (L) and **AB** by ^{1}H NMR analysis.

Crystal data for $(Pd_3\mathbf{L}Cl_6)_2\cdot(\mathbf{AB})_{0.43}\cdot(DME)_{2.85}\cdot(H_2O)_3$.: $C_{100.48}H_{116.65}Cl_{12}N_{12.85}O_{8.68}Pd_6$, F_w = 2707.02, crystal

dimensions 0.19 × 0.14 × 0.06 mm^3, monoclinic, space group $P2_1/c$, a = 22.02650(15), b = 51.5831(5), c = 14.60220(7) Å, β = 97.5355(6)°, V = 16447.7(2) Å^3, Z = 4, ρ_{calcd} = 1.093 g cm^{-3}, μ = 7.297 mm^{-1}, T = 93 K, λ(CuKα) = 1.54187 Å, $2\theta_{max}$ = 136.5°, 173810/ 30051 reflections collected/unique (R_{int} = 0.0543), R_1 = 0.1136 ($I > 2\sigma(I)$), wR_2 = 0.3701 (for all data), GOF = 1.577, largest diff. peak and hole 6.663/–4.329 eÅ^{-3}. CCDC deposit number 2223950

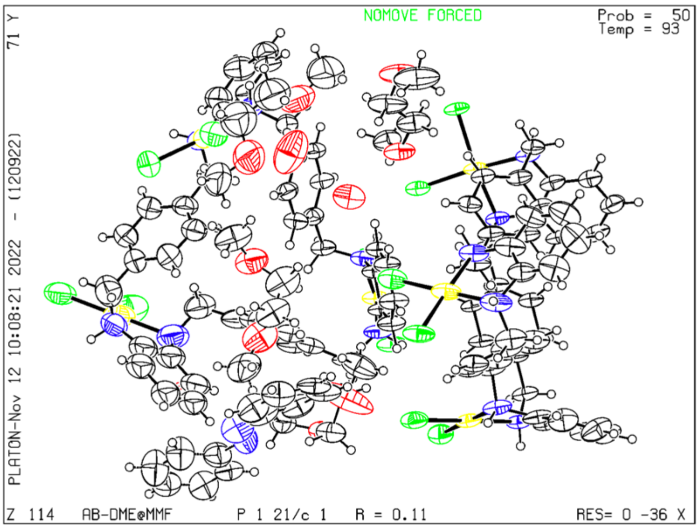

Supplementary Fig. 129 ORTEP drawing of the structurally-extended MMF soaked in 1.0 M **DME solution of AB** at the 50% probability level. Color: C black, N blue, O red, Cl green and Pd yellow. This figure was produced by the checkCIF report of the International Union of Crystallography.

–in DME:MeCN = 95:5(v:v)

Soaking procedure: MMF crystals were soaked in a DME:MeCN = 95:5(v:v) solution of ***trans*-azobenzene** (1.0 M) at 20 °C for 1 day, and one of the crystals was picked up and immediately mixed with paratone oil to measure single-crystal XRD. Other crystals were filtered and washed with 500 μL DME:MeCN = 95:5(v:v) to remove the guest non-specifically adsorbed on the crystal surface, and then dissolved in DMSO-d_6/DCl-D_2O to determine the molar ratio of the protonated macrocycle ligand (L) and **AB** by ^{1}H NMR analysis.

Crystal data for $(Pd_3\mathbf{L}Cl_6)_4\cdot(MeCN)_2\cdot(H_2O)_2$.: $C_{172}H_{174}Cl_{24}N_{26}O_2Pd_{12}$, F_w = 4764.96, crystal dimensions 0.66 × 0.38 × 0.07 mm^3, monoclinic, space group $P2_1$, a = 14.3751(1), b = 51.7769(10), c = 19.5386(3) Å, β = 91.1316(10)°, V = 14539.7(4) Å^3, Z = 2, ρ_{calcd} = 1.088 g cm^{-3}, μ = 8.155 mm^{-1}, T = 93 K, λ(CuKα) = 1.54187 Å, $2\theta_{max}$ = 136.5°, 154326/ 52516 reflections collected/unique (R_{int} = 0.0777), R_1 = 0.1333 ($I > 2\sigma(I)$), wR_2 = 0.4030 (for all data), GOF = 1.588, largest diff. peak and hole 4.673/–3.559 eÅ^{-3}, Flack parameter = 0.44(2). CCDC deposit number 2223951

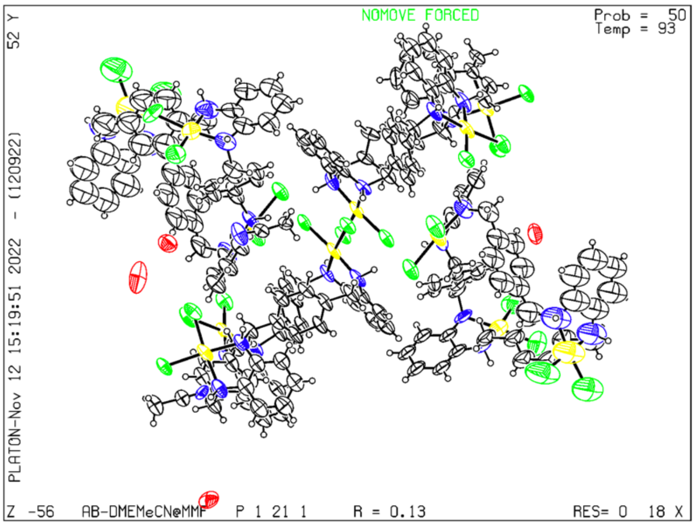

Supplementary Fig. 130 ORTEP drawing of not-extended MMF soaked in 1.0 M **DME:MeCN = 95:5 (v:v) solution of AB** at the 50% probability level. Color: C black, N blue, O red, Cl green and Pd yellow. This figure was produced by the checkCIF report of the International Union of Crystallography.

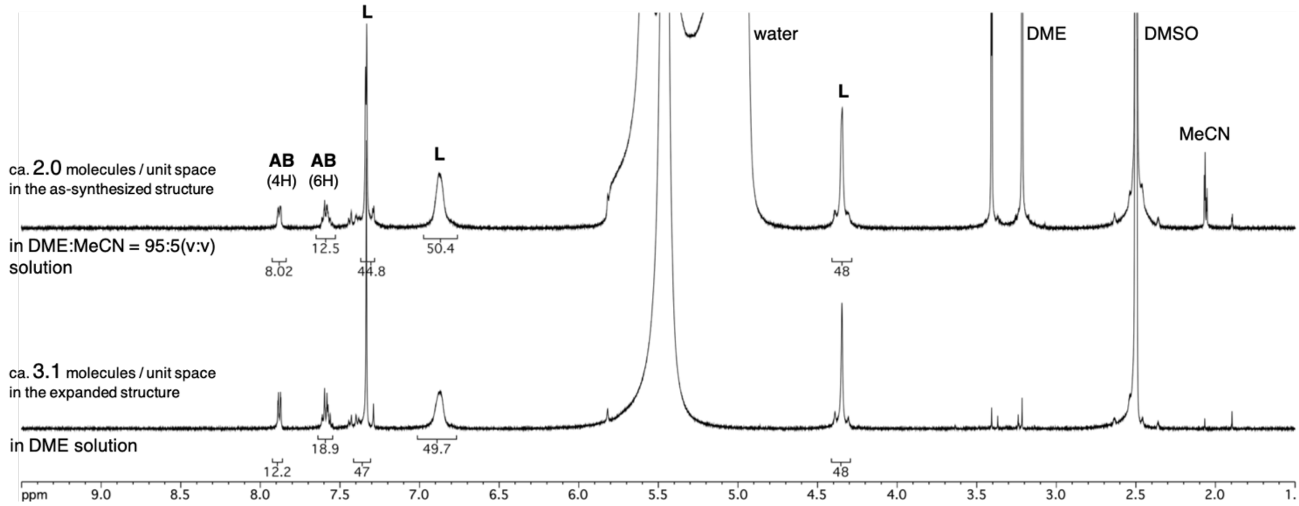

Supplementary Fig. 131 ^{1}H NMR spectra (500 MHz, DMSO-d_6, 300 K) after dissolving MMF crystals uptaking **AB** (upper) in DME:MeCN = 95:5 (v:v) and (bottom) in a DME solution, confirming that **AB** was sufficiently introduced in both nanochannels.

•*N*-(Benzyloxycarbonyl)-L-serine (**Z-Ser**)

–in 1,4-dioxane

Soaking procedure: MMF crystals were soaked in a 1,4-dioxane solution of ***N*-(benzyloxycarbonyl)-L-serine** (1.0 M) at 20 °C for 1 day, and one of the crystals was picked up and immediately mixed with paratone oil to measure single-crystal XRD. Other crystals were filtered and washed with 500 µL MeCN to remove the guest non-specifically adsorbed on the crystal surface, and then dissolved in DMSO-d_6/DCl-D_2O to determine the molar ratio of the protonated macrocycle ligand (L) and **Z-Ser** by ^{1}H NMR analysis.

Crystal data for $(Pd_3\mathbf{L}Cl_6)_4{\cdot}(1,4\text{-dioxane})_{7.38}{\cdot}(H_2O)_{1.77}$.: $C_{197.52}H_{227.04}Cl_{24}N_{24}O_{16.53}Pd_6$, F_w = 5329.38, crystal dimensions 0.38 × 0.26 × 0.06 mm^3, monoclinic, space group $P2_1$, a = 14.3805(1), b = 54.3527(3), c = 20.0307(1) Å, β = 90.0700°, V = 15656.36(16) Å^3, Z = 2, ρ_{calcd} = 1.130 g cm^{-3}, μ = 7.655 mm^{-1}, T = 93 K, λ(CuKα) = 1.54187 Å, $2\theta_{max}$ = 136.5°, 422681/ 56999 reflections collected/unique (R_{int} = 0.0550), R_1 = 0.0924 ($I > 2\sigma(I)$), wR_2 = 0.2802 (for all data), GOF = 1.253, largest diff. peak and hole 4.389/–2.033 eÅ^{-3}, Flack parameter = 0.265(12). CCDC deposit number 2223952

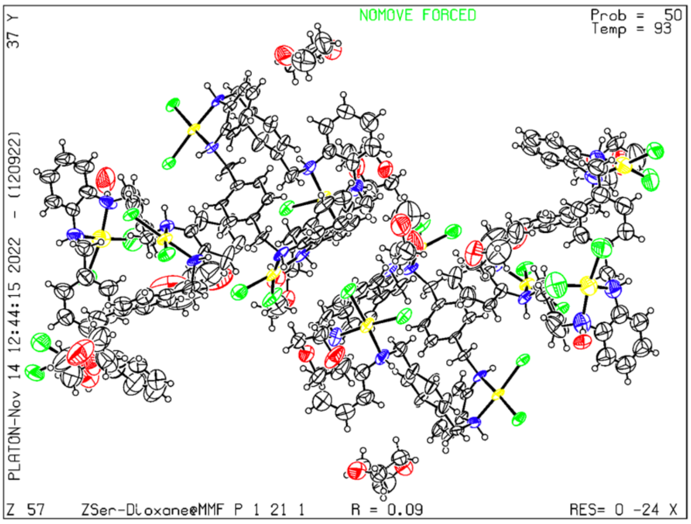

Supplementary Fig. 132 ORTEP drawing of the structurally-extended MMF soaked in 1.0 M **1,4-dioxane solution of Z-Ser** at the 50% probability level. Color: C black, N blue, O red, Cl green and Pd yellow. This figure was produced by the checkCIF report of the International Union of Crystallography.

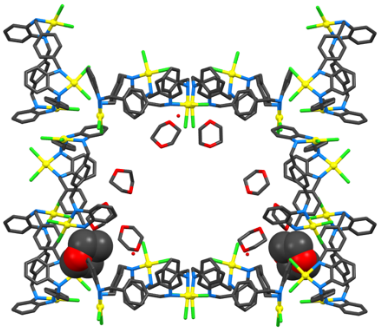

Supplementary Fig. 133 The unit-space structure of the structurally extended MMF (effector: 1,4-dioxane) soaked in a 1,4-dioxane solution of **Z-Ser**.

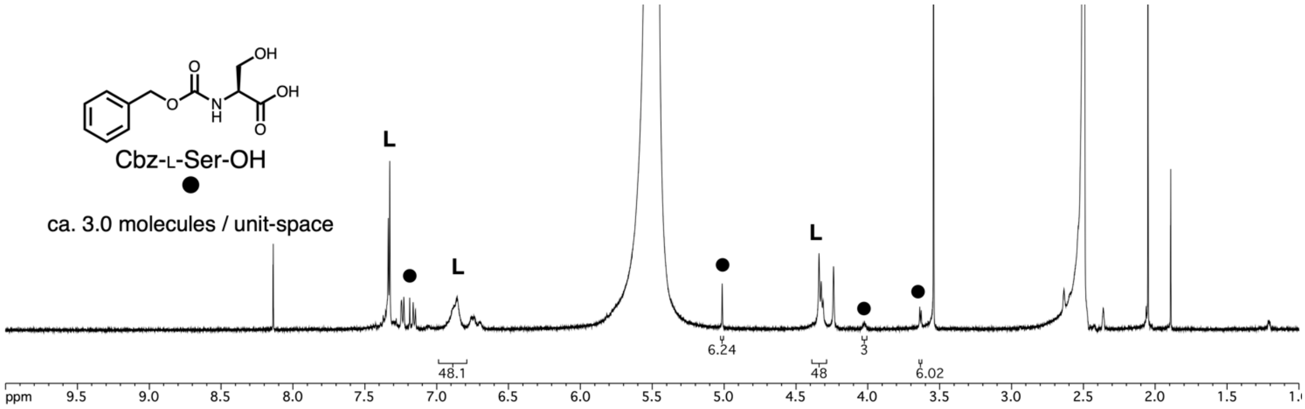

Supplementary Fig. 134 ^{1}H NMR spectrum (500 MHz, DMSO-d_6, 300 K) after dissolving MMF crystals that adsorbed **Z-Ser** in a 1,4-dioxane solution, showing that **Z-Ser** was sufficiently introduced into the nanochannel.

−1,4-dioxane:MeCN = 50:50(v:v)

Soaking procedure: MMF crystals were soaked in a 1,4-dioxane:MeCN = 50:50 (v:v) solution of ***N*-(benzyloxycarbonyl)-L-serine** (500 mM) at 20 °C for 1 day, and one of the crystals was picked up and immediately mixed with paratone oil to measure single-crystal XRD.

Crystal data for $(Pd_3\mathbf{L}Cl_6)_4\cdot(\mathbf{Z\text{-}Ser})_{1.39}\cdot(MeCN)_5\cdot(1,4\text{-dioxane})_1\cdot(H_2O)_{2.}$: $C_{187.57}H_{195.17}Cl_{24}N_{30.39}O_{8.17}Pd_{12}$, F_w = 5133.60, crystal dimensions 0.34 × 0.11 × 0.08 mm^3, monoclinic, space group $P2_1$, a = 14.2975(1), b = 52.3236(2), c = 19.6886(1) Å, β = 91.1900°, V = 14725.82(14) Å^3, Z = 2, ρ_{calcd} = 1.158 g cm^{-3}, μ = 8.107 mm^{-1}, T = 93 K, λ(CuKα) = 1.54187 Å, $2\theta_{max}$ = 136.5°, 385676/ 53685 reflections collected/unique (R_{int} = 0.0639), R_1 = 0.1297 ($I > 2\sigma(I)$), wR_2 = 0.3520 (for all data), GOF = 1.652, largest diff. peak and hole 4.598/–2.026 eÅ^{-3}, Flack parameter = 0.173(15). CCDC deposit number 2223953

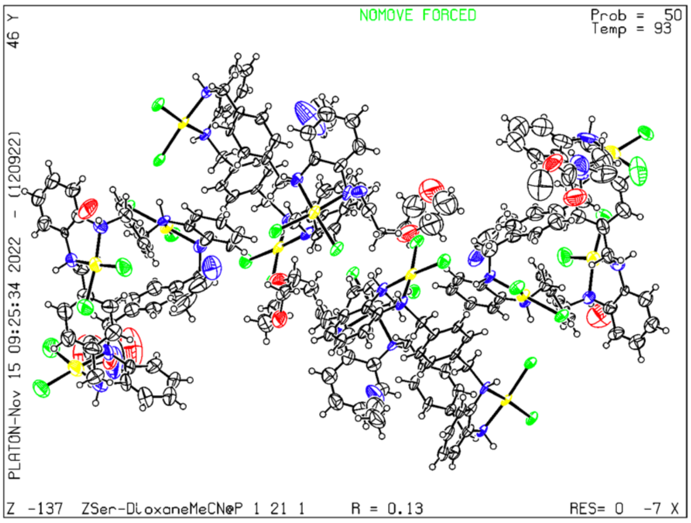

Supplementary Fig. 135 ORTEP drawing of not-extended MMF soaked in a 500 mM **1,4-dioxane:MeCN = 50:50 (v:v) solution of Z-Ser** at the 50% probability level. Color: C black, N blue, O red, Cl green and Pd yellow. This figure was produced by the checkCIF report of the International Union of Crystallography.

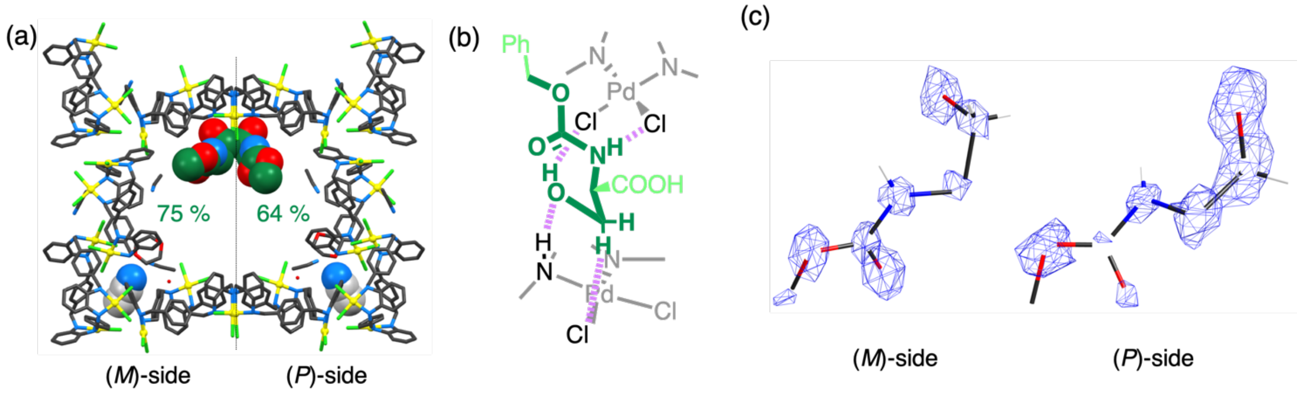

Supplementary Fig. 136 (a) The unit-space structure and (b) host-guest interaction patterns of the structurally as-crystalised MMF (effector: MeCN) that adsorbed **Z-Ser** in 1,4-dioxane:MeCN = 50:50 (v:v), which are similar to the previous paper[5]. Dark green indicates the assigned parts of **Z-Ser**, and light green indicates the disordering parts. (c) Electron density maps of the adsorbed **Z-Ser** (contour level; 2.0 eÅ^{-3} for (*M*)-side, 1.2 eÅ^{-3} for (*P*)-side).

•*p*-Dibromobenzene (DBB)

−in DME

Soaking procedure: MMF crystals were soaked in a DME solution of ***p*-dibromobenzene** (500 mM) at 20 °C for 1 day, and one of the crystals was picked up and immediately mixed with paratone oil to measure single-crystal XRD.

Crystal data for $(Pd_3\mathbf{L}Cl_6)_2\cdot(\mathbf{DBB})_{0.42}\cdot(DME)_{1.5}\cdot(H_2O)_{3.58}$.: $C_{92.47}H_{100.65}Br_{0.82}Cl_{12}N_{12}O_{6.59}Pd_6$, F_w = 2615.29, crystal dimensions 0.14 × 0.08 × 0.05 mm^3, monoclinic, space group $P2_1/c$, a = 22.1728(9), b = 51.542(3), c = 14.4940(3) Å, β = 97.301(3)°, V = 16429.9(12) Å^3, Z = 4, ρ_{calcd} = 1.057 g cm^{-3}, μ = 7.505 mm^{-1}, T = 93 K, λ(CuKα) = 1.54187 Å, $2\theta_{max}$ = 136.5°, 70600/ 29557 reflections collected/unique (R_{int} = 0.0894), R_1 = 0.1423 ($I > 2\sigma(I)$), wR_2 = 0.4401 (for all data), GOF = 1.295, largest diff. peak and hole 2.908/–2.864 eÅ^{-3}. CCDC deposit number 2223954

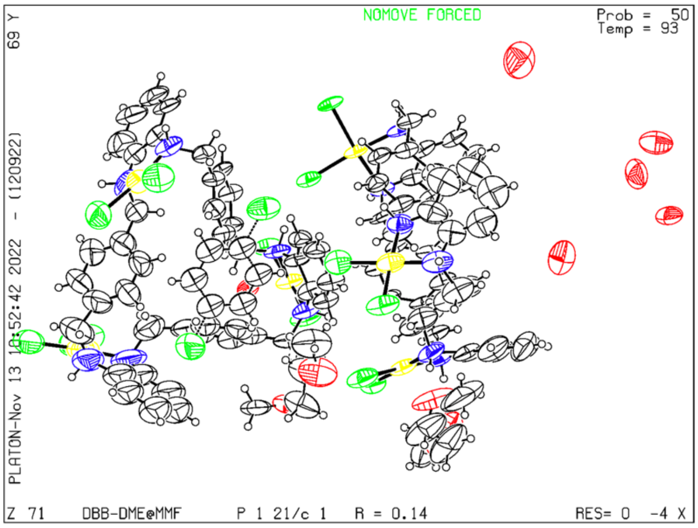

Supplementary Fig. 137 ORTEP drawing of the structurally-extended MMF soaked in a 500 mM **DME solution of DBB** at the 50% probability level. Color: C black, N blue, O red, Cl/Br green and Pd yellow. This figure was produced by the checkCIF report of the International Union of Crystallography.

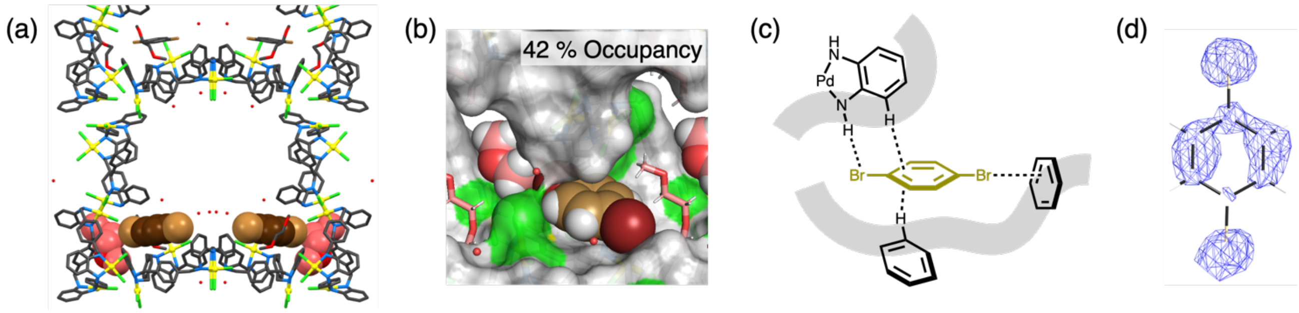

Supplementary Fig. 138 (a) The unit-space structure, (b) channel surface, and (c) host-guest interaction patterns of the structurally extended MMF (effector: DME) adsorbing **DBB** in DME. (d) Electron density maps of the adsorbed **DBB** (contour level; 1.3 eÅ^{-3}).

–DME:MeCN = 95:5(v:v)

Soaking procedure: MMF crystals were soaked in a DME:MeCN = 95:5(v:v) solution of ***p*-dibromobenzene** (500 mM) at 20 °C for 1 day, and one of the crystals was picked up and immediately mixed with paratone oil to measure single-crystal XRD.

Crystal data for $(Pd_3LCl_6)_2\cdot$(**DBB**)$_{0.39}\cdot(MeCN)_{2.66}\cdot(H_2O)_{3.73}$.: $C_{91.65}H_{93.55}Br_{0.77}Cl_{12}N_{14.67}O_{3.76}Pd_6$, F_w = 2586.02, crystal dimensions 0.42 × 0.25 × 0.07 mm^3, monoclinic, space group $P2_1/c$, a = 19.5606(3), b = 52.0514(9), c = 14.30740(13) Å, β = 91.139(1)°, V = 14564.3(4) Å^3, Z = 4, ρ_{calcd} = 1.179 g cm^{-3}, μ = 8.436 mm^{-1}, T = 93 K, λ(CuKα) = 1.54187 Å, $2\theta_{max}$ = 136.5°, 146558/ 26669 reflections collected/unique (R_{int} = 0.1088), R_1 = 0.1169 ($I > 2\sigma(I)$), wR_2 = 0.3341 (for all data), GOF = 1.098, largest diff. peak and hole 3.355/–3.007 eÅ^{-3}, Flack parameter = 0.423(19). CCDC deposit number 2223955

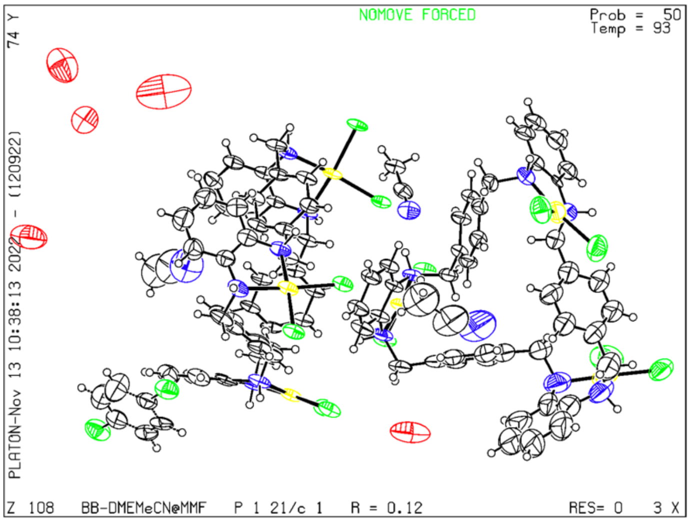

Supplementary Fig. 139 ORTEP drawing of not-extended MMF soaked in 500 mM **DME:MeCN = 95:5 (v:v) solution of DBB** at the 50% probability level. Color: C black, N blue, O red, Cl/Br green and Pd yellow. This figure was produced by the checkCIF report of the International Union of Crystallography.

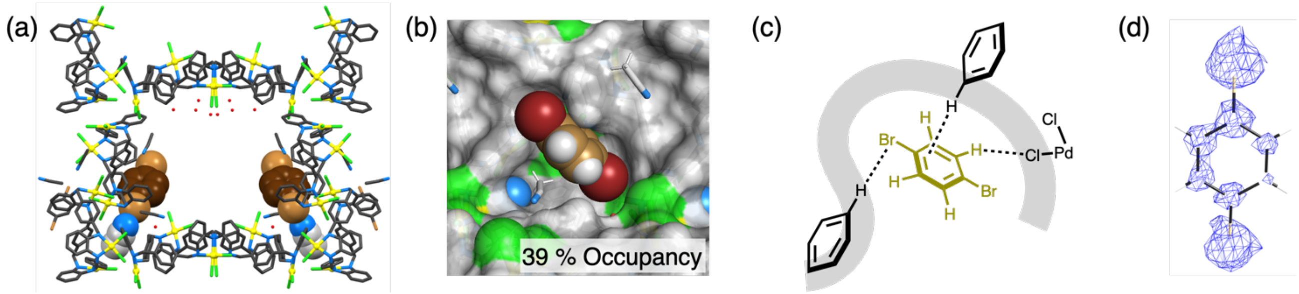

Supplementary Fig. 140 (a) The unit-space structure, (b) channel surface and (c) host-guest interaction patterns of the structurally as-crystalised MMF (effector: MeCN) adsorbing **DBB** in DME:MeCN = 95:5 (v:v). (d) Electron density maps of the adsorbed **DBB** (contour level; 1.4 eÅ^{-3}).

7. Organic synthesis

Synthesis of pentaethyleneglycol dimethyl ether (**pentaglyme**)[6]

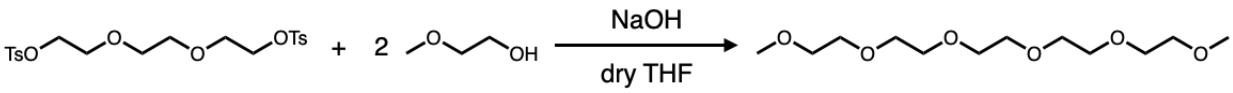

Sodium hydroxide pellets (3.02 g, 75.5 μmol) was put in a three-necked flask and dry THF (150 mL) was introduced under Ar atmosphere. To the stirred suspension at 66 °C was added 2-methoxyethanol (2.75 mL, 35.1 μmol) in dry THF (50 mL), and stirring was continued at 66 °C for 3 h with bubbling of Ar. Triethyleneglycol bis(*p*-toluenesulfonate) (6.89 g, 15.0 μmol) in dry THF (75 mL) was then added dropwise to the stirred mixture over 30 min, and stirring was continued at 66 °C for 21 h under Ar atmosphere. The solvent was evaporated and the residue was extracted with chloroform. The extract was dried over $MgSO_4$ and the solvent was evaporated to give pale-yellow liquid. The subsequent Kugel-Rohr distillation under a reduced

pressure yielded pentaglyme as a colorless liquid (0.802 g, 3.01 μmol, 20%). The obtained NMR spectrum was in good agreement with the published data[7].

^{1}H NMR (500 MHz, $CDCl_3$, 300 K): δ = 3.68–3.63 (m, 16H), 3.57–3.53 (m, 4H), 3.38 ppm (s, 6H)

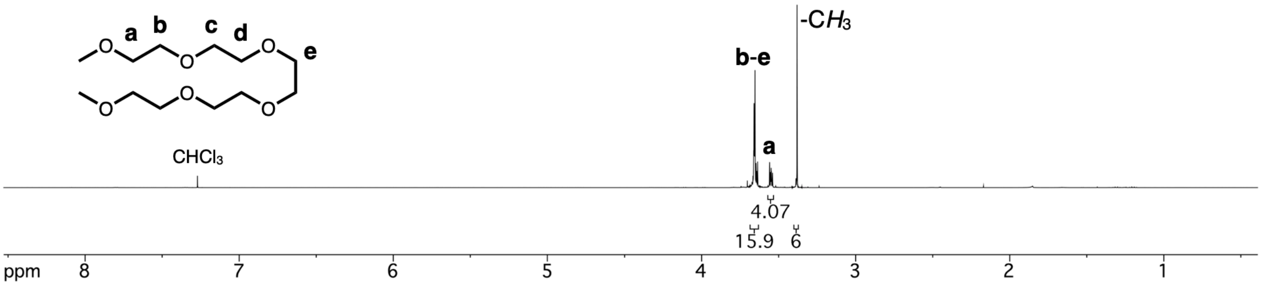

Supplementary Fig. 141 ^{1}H NMR spectrum of **pentaglyme** (500 MHz, $CDCl_3$, 300 K)

8. References

[1] S. Tashiro, R. Kubota, M. Shionoya, *J. Am. Chem. Soc.* **2012**, *134*, 2461.

[2] O. V. Dolomanov, L. J. Bourhis, R. J. Gildea, J. A. K. Howard, H. Puschmann, *J. Appl. Cryst.* **2009**, *42*, 339.

[3] G. M. Sheldrick, *Acta Cryst.* **2015**, *C71*, 3.

[4] C. B. Hübschle, G. M. Sheldrick, B. Dittrich, *J. Appl. Cryst.* **2011**, *44*, 1281.

[5] S. Tashiro, K. Nakata, R. Hayashi, M. Shionoya, *Small.* **2021**, *17*, 2005803.

[6] M. Ouchi, Y. Inoue, T. Kanzaki, T. Hakushi, *J. Org. Chem.* **1984**, *49*, 1408.

[7] M. Jozwaik, M. A. Kosiorowska, A. Jozwaik, *J. Chem. Eng. Data* **2010**, *55*, 5941.
